# Distinct gut microbiota and metabolomic profiles in HBV-related liver cirrhosis: insights into disease progression

**DOI:** 10.3389/fcimb.2025.1560564

**Published:** 2025-05-19

**Authors:** Ke Shi, Lina Sun, Ying Feng, Xianbo Wang

**Affiliations:** Center of Integrative Medicine, Beijing Ditan Hospital, Capital Medical University, Beijing, China

**Keywords:** gut microbiota, cirrhosis, hepatitis B virus, 16S rRNA sequencing, metabolomics

## Abstract

**Background:**

Hepatitis B virus (HBV)-related liver cirrhosis (HBV-LC) is a significant global health issue, affecting gut microbiota (GM) composition and metabolic processes. This study aimed to explore the associations between intestinal microbiota, metabolic profiles, and disease progression in patients with HBV-LC.

**Methods:**

Fecal samples were collected prospectively from 40 healthy controls (HC) and 83 HBV-LC patients between December 2022 and August 2023. Gut microbiota alterations at various stages of liver function were analyzed using 16S rRNA gene sequencing. Untargeted metabolomics was employed to identify potential biomarkers and metabolic pathways associated with early cirrhosis. Additionally, correlations between bacterial genera, inflammatory markers, and metabolites were investigated.

**Results:**

HBV-LC patients demonstrated a significant reduction in bacterial diversity and relative abundance compared to the HC group. Genera such as *Alistipes* and *Lachnospira* were notably depleted, while *Fusobacterium* and *Enterococcus* were enriched in patients with Model for End-Stage Liver Disease (MELD) scores ≥ 21 or Child-Turcotte-Pugh C grade. Correlation analyses revealed strong associations between intestinal flora, clinical indicators of disease severity, and inflammatory factors. Metabolic analysis showed decreased levels of tocopherol and 21-hydroxypregnenolone, which were strongly linked to the reduced abundance of *Alistipes* and *Lachnospira*. Biosynthesis of unsaturated fatty acids and linoleic acid metabolism emerged as critical enrichment pathways.

**Conclusions:**

HBV-LC patients displayed significant alterations in gut microbiota and fecal metabolites, which correlated closely with disease severity and inflammatory status. These findings provide new insights into cirrhosis pathogenesis and suggest potential biomarkers for early diagnosis and disease monitoring.

## Introduction

Hepatitis B virus (HBV) infection remains a global health concern, with over 250 million individuals affected worldwide (Hsu et al., 2023). HBV-related liver cirrhosis (LC) serving as an advanced pathological stage of liver disease (Lim et al., 2023). Patients with cirrhosis face elevated risks of liver failure and cancer, significantly affecting their quality of life (Ginès et al., 2021). Moreover, complications such as hepatic encephalopathy and infections substantially contribute to higher rates of disability and mortality (Huang et al., 2023). Nucleoside/nucleotide analogs, the first‐line anti‐viral medications, have slowed down chronic hepatitis B progression, but they cannot completely stop the development of liver-related events (Kozuka et al., 2024). Consequently, the vigilant monitoring of disease progression is imperative for patients with HBV-associated cirrhosis.

With the deepening understanding of the gut-liver axis interaction, the connection between gut microbiota (GM) and cirrhosis has attracted growing attention (Zhang et al., 2024; Ali et al., 2023). GM play a vital role in the pathogenesis and progression of liver cirrhosis, and early detection and intervention of intestinal disorders may help slow the disease’s advancement (Shen et al., 2023; Trebicka et al., 2021a). Disruptions in the GM can increase intestinal permeability, allowing microbial products, such as lipopolysaccharides (LPS), to enter the liver and trigger systemic inflammation and liver damage (Di Vincenzo et al., 2024). These microbial-derived signals have been shown to impact liver inflammation, fibrosis progression, and the overall severity of cirrhosis. In patients with decompensated cirrhosis, GM dysbiosis often leads to pro-inflammatory events such as infections, bacterial translocation, or both (Trebicka et al., 2021b; Baffy, 2019). However, the relationships between GM, clinical indicators, and inflammatory factors remain poorly understood. Previous studies have shown that in patients with decompensated cirrhosis, the abundance of *Ruminococcaceae* is significantly reduced, while the richness of *Enterobacteriaceae* is relatively higher when compared to the compensated group (Shu et al., 2022). While these studies have focused primarily on microbiome composition changes in HBV-associated cirrhosis, little research has explored the connections between GM changes, inflammatory status, and disease severity.

In cirrhotic patients, dysbiosis leads to significant changes in the host’s metabolic profile, contributing to complications such as hepatic encephalopathy (Zhu et al., 2023). Increasing evidence suggests that metabolites derived from gut microbes, including short-chain fatty acids, tryptophan, and bile acids, play a critical role in the onset and progression of liver disease (Xu et al., 2022; Xue et al., 2021). Therefore, a comprehensive analysis of both the microbiome and metabolome may provide insights into the disease severity in cirrhosis. Given the rising prevalence of HBV-related cirrhosis and its severe complications, understanding the microbial and metabolic shifts during disease progression is crucial. There is an urgent need for research that integrates microbiological and metabolic approaches to better elucidate these interactions and their clinical implications.

In this study, we aim to investigate the microbiome profile and metabolic changes throughout the progression of HBV-related cirrhosis using 16S rRNA gene amplicon sequencing and liquid chromatography-mass spectrometry (LC-MS).

## Materials and methods

### Participant recruitment and sample collection

All 109 fecal samples from patients with HBV-associated cirrhosis who were treated with entecavir or tenofovir were prospectively collected from Beijing Ditan Hospital between December 2022 and August 2023. Chronic hepatitis B is defined as hepatitis B surface antigen positivity for more than 6 months (Sarin et al., 2016). Cirrhosis diagnosis is based on liver biopsy, ultrasound, elastography, imaging, or endoscopy showing signs of portal hypertension (Xu et al., 2020). Exclusion criteria included: (a) age less than 18 or over 70 years; (b) antibiotics, probiotics, proton pump inhibitors, and lactulose treatment within the last three months; hypertension, diabetes, or metabolic syndrome; (d) liver tumors or other malignancies; (e) other viral hepatitis, alcoholic hepatitis, autoimmune liver disease, or human immunodeficiency virus infection; and (f) alcoholism, pregnancy, or incomplete information. After exclusion, 83 patients were included in the analysis. Demographic and laboratory data were recorded within 48 hours of hospitalization. The Child-Turcotte-Pugh (CTP) and Model for End-Stage Liver Disease (MELD) scores were calculated to assess liver disease severity. Forty healthy control (HC) individuals, matched in terms of age, sex, and body mass index (BMI) (aged 18–70 years), were included in this analysis. The HC group did not have liver or renal diseases, diabetes, hypertension, gastrointestinal diseases, or other conditions that could impact gut bacteria.

Fresh stool samples were collected with informed consent and stored at –80 °C until further analysis. Blood samples were obtained from all participants at enrollment for liver function, renal function, routine blood tests, and plasma cytokines. This study adhered to the ethical guidelines outlined in the 1975 Declaration of Helsinki and received approval from the Ethics Committee of Beijing Ditan Hospital (approval number: 2021-089). This study was registered in the Chinese Clinical Trial Registry, International Clinical Trials Registry Platform of the World Health Organization (ChiCTR2000040465). Written informed consent was obtained from each patient for using their data in the study.

### DNA extraction and sequencing

The genomic DNA from the intestinal flora was extracted using a Stool DNA Kit (Magen, #D6356-02). The taxonomic composition of the intestinal bacteria was assessed by targeting the V3-V4 highly variable region of fecal 16S rRNA. Amplification of the V3-V4 variable regions of 16S rRNA genes was carried out using universal primers 343F (5’-TACGGRAGGCAGCAG-3’) and 798R (5’-AGGGTATCTAATCCT-3’). The quality of the amplicons was verified through agarose gel electrophoresis and subsequent purification. Sequencing was conducted using the Illumina NovaSeq 6000 sequencing platform (Illumina, San Diego, CA, USA).

### Sample preparation and LC-MS analysis

A 50 mg sample was weighed, and 400 μL of a methanol-aqueous solution containing L-2-chlorophenylalanine was added. The mixture was pre-cooled for 2 minutes, ground, and then subjected to ultrasound extraction in an ice water bath for 10 minutes. After extraction, the mixture was allowed to sit for 30 minutes before centrifugation. The supernatant was collected, dried, and re-dissolved in the methanol-aqueous solution. Ultrasonic treatment, followed by static and centrifugal processes, was performed again. The resulting supernatant was filtered using a 0.22 μm filter and transferred to an LC-MS injection vial, where it was stored at -80°C. Quality control samples were prepared by combining extracts from all the samples.

For analysis, an ACQUITY UPLC I-Class plus liquid chromatography system and QE plus mass spectrometry system were employed. The metabolic spectrum was analyzed using an ACQUITY UPLC HSS T3 column in both positive and negative ion modes. The elution gradient consisted of water and acetonitrile, with a flow rate of 0.35 mL/min and a column temperature of 45°C. The mass spectrometry scan range was 100–1200 m/z, with a resolution of 70,000 and collision energies set at 10, 20, and 40 eV.

### Bioinformatic analyses

The raw reads were filtered to remove low-quality sequences. The remaining high-quality reads were clustered into operational taxonomic units (OTUs) with 97% similarity using VSearch software. Representative reads for each OTU were selected and annotated using QIIME (version 1.9.1) and the Silva database (version 138). Several indices, including Chao 1, abundance-based coverage estimator (ACE), Shannon, and Simpson, as well as beta diversity, were analyzed with QIIME. Principal coordinate analysis (PCoA) was performed based on the unweighted UniFrac distance matrix to visualize the microbiome differences across groups. The linear discriminant analysis effect size (LEfSe) method was used to identify genus-level abundance differences.

To differentiate metabolite profiles between groups, orthogonal partial least squares discriminant analysis (OPLS-DA) and partial least squares discriminant analysis (PLS-DA) were employed. To prevent overfitting, model performance was assessed using 7-fold cross-validation and a 200-time response alignment test. The variable importance in projection (VIP) values was derived from the OPLS-DA model, ranking the contribution of each variable. The significance of differential metabolites was further validated by a two-tailed Student’s t-test. Metabolites with a VIP value greater than 1.0 and a p-value less than 0.05 were considered significant and selected as differential metabolites.

### Analysis of 8 cytokines/chemokines using Luminex technology

The levels of Chemokine (C-X-C motif) Ligand 1 Protein (CXCL1), CXCL2, tumor necrosis factor-α (TNF-α), interferon γ (IFN-γ), granulocyte-macrophage colony stimulating factor (GM-CSF), interleukin-1β (IL-1β), IL-6, and IL-10 were measured with the Human Chemokine Panel 8-plex kit (PPX-08-MXCE6ZC) on the Luminex 200 platform (Luminex, USA). Subsequently, correlation analyses were conducted between different genera and these cytokines/chemokines.

### Statistical analysis

Statistical analyses were conducted using SPSS software (version 25.0) and R software (version 4.2.3). Continuous variables were presented as median and interquartile range or mean ± standard deviation, while categorical variables were expressed as percentages. The Student’s t-test or Mann–Whitney U test was employed for comparing continuous variables, and chi-square tests were used for categorical variables. Spearman correlation analysis was performed to assess the association among GM, metabolites and cytokines/chemokines levels. Statistical significance was set at *P* < 0.05 for a two-tailed test.

## Results

### Baseline characteristics

**Figure 1:**
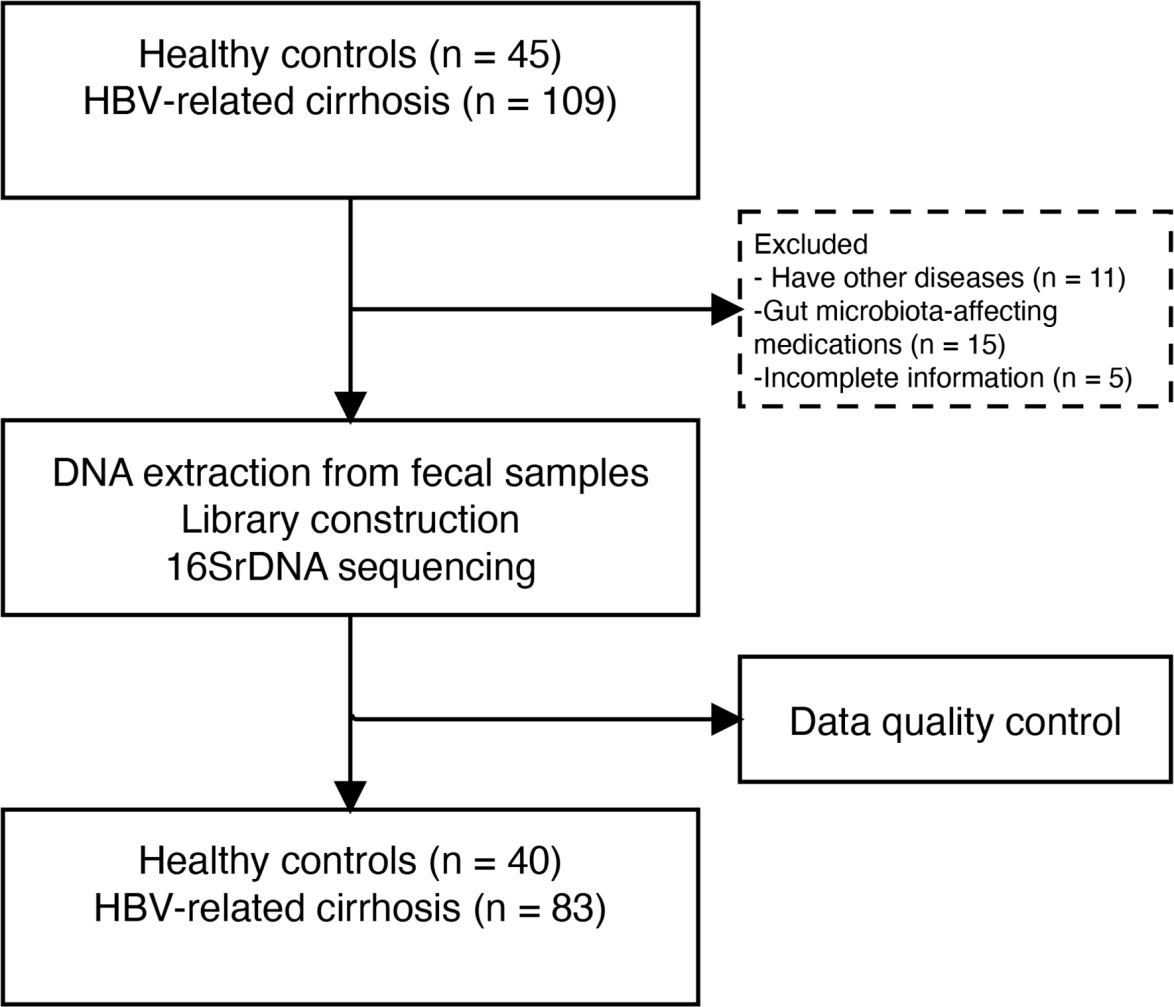
Research design and flow chart.

In this study, 154 samples were screened, excluding 11 patients with comorbidities, 15 individuals on GM-affecting medications, and 5 HC with incomplete information. Finally, we analyzed fecal samples from 83 patients with HBV-LC and 40 HC matched for age, sex, and BMI (**Figure 1**).

The characteristics of the healthy controls and patients with HBV-LC are summarized in **Table 1**. The median age of patients was 49.0 (43.0, 57.0) years, with 61 (73.4%) males in the LC group. Among the patients, 42 (50.6%) had ascites, and 14 (16.8%) had hepatic encephalopathy. The median total bilirubin (TBIL) level was 18.8 µmol/L, and the MELD score was 10.9. The distribution of CTP grades was as follows: 31 (37.3%) in grade A, 30 (36.2%) in grade B, and 22 (26.5%) in grade C. Most patients had HBV DNA levels ≤20 IU/mL, with 67 being HBeAg-negative. Furthermore, all patients had received antiviral treatment for more than one year.

**Table 1:** Clinical baseline characteristics of patients with liver cirrhosis and overt hepatic encephalopathy in the training and validation sets.

Variables	Healthy controls (n = 40)	Cirrhosis (n = 83)	*P* value
Age (years)	49.0 (46.0,53.0)	49.0 (43.0,57.0)	0.505
Male (%)	24 (60.0)	61 (73.4)	0.129
Body mass index	24.2 (21.8,27.6)	22.8 (20.9,26.0)	0.238
Alcohol consumption (%)	4 (10)	10 (12)	0.217
Ascites (%)	–	42 (50.6)	–
HE(%)	–	14 (16.8)	–
ALT (IU/L)	16.5 (11.2,28.0)	26.1 (15.9,44.7)	0.034
AST (IU/L)	17.2 (15.5,21.4)	36.1 (22.5,62.0)	0.002
TBIL (µmol/L)	11.0 (9.0,12.4)	18.8 (13.0,72.9)	< 0.001
Albumin (g/L)	48.3 ± 2.4	33.9 ± 6.2	< 0.001
NLR	1.8 (1.2,2.4)	2.1 (1.4,3.1)	0.037
PLT (×10^9^/L)	210.0 (187.0,258.0)	97.0 (55.2,133.0)	0.001
INR	–	1.2 (1.2,1.7)	–
Cr (µmol/L)	64.5 (59.2,76.0)	64.7 (55.6,76.4)	0.483
HBV DNA (IU/mL)			–
≤ 20	–	56 (67.5)	
102 -10^5^	–	16 (19.3)	
≥ 10^5^	–	11 (13.2)	
HBeAg			–
Positive	–	16 (19.3)	
Negative	–	67 (80.7)	
Antiviral therapy			–
MELD score	–	10.9 (8.3,15.9)	–
CTP (A/B/C)	–	31/30/22	–

Data are presented as n (%), mean ± SD, or median (interquartile range).

HE, hepatic encephalopathy; ALT, alanine aminotransferase; AST, aspartate aminotransferase; TBIL, total bilirubin; MELD, Model for End-Stage Liver Disease; CTP, Child-Turcotte-Pugh; NLR, neutrophil–lymphocyte ratio; INR, international normalized ratio; Cr, serum creatinine; PTA, prothrombin activity.

### Changes in gut microbiome in the LC group

**Figure 2:**
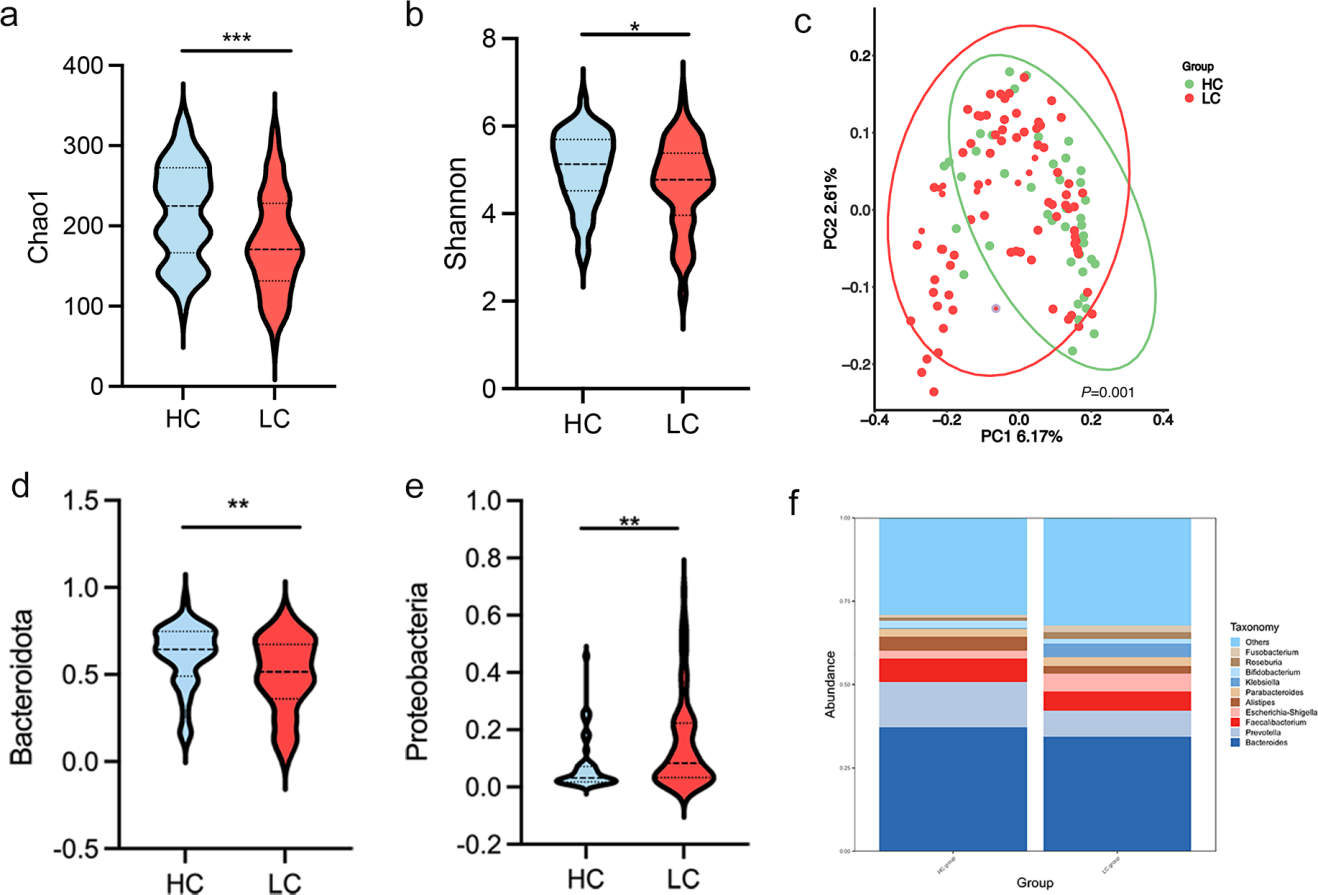
Gut microbiota diversity and composition in healthy controls (HC) and patients with HBV-related liver cirrhosis (HBV-LC). **(a, b)** Chao 1 and Shannon diversity indices comparing HC and HBV-LC; **(c)** Principal Coordinate Analysis (PCoA) plot based on weighted UniFrac distances, showing significant clustering differences between HC and HBV-LC groups;(permutation test, *P* = 0.001). **(d, e)** Relative abundance of bacterial taxa at the phylum level (*P < 0.05, **P < 0.01, ***P < 0.001). **(f)** Differential abundance of gut microbiota at the genus level.

**Figure 3:**
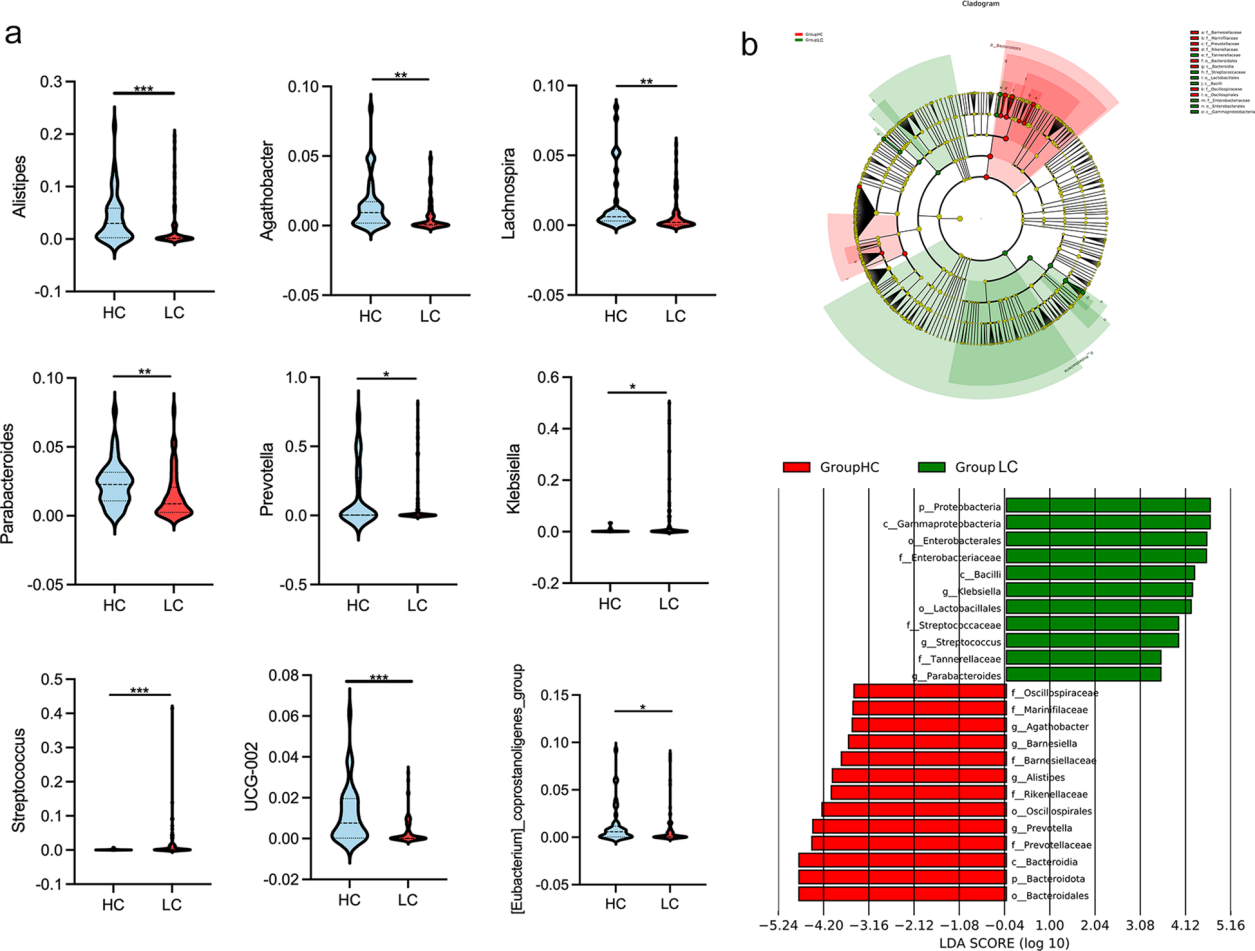
Genus-level differences in gut microbiota between healthy controls (HC) and patients with HBV-related liver cirrhosis (HBV-LC). **(a)** Comparison of significantly altered bacterial genera between HC and HBV-LC groups (*P < 0.05, **P < 0.01, ***P < 0.001). **(b)** Linear discriminant analysis (LDA) highlighting bacterial communities with LDA scores > 4.0.

Differences in gut microbial diversity were observed between HC and HBV-LC patients (both *P* < 0.05; **Figures 2a, b**). Alpha diversity, as measured by the Chao1 and Shannon indices, was significantly lower in the LC group compared to HC group. Beta diversity analysis using PCoA also showed differences in microbial composition between the two groups (**Figure 2c**). At the phylum level, the LC group had an increased abundance of *Proteobacteria* and a decreased abundance of *Bacteroidota* (both *P* < 0.05; **Figures 2d–f**). Differentially abundant genera are shown in **Figure 2a**. The LC group exhibited higher levels of the harmful genera *Klebsiella* and *Streptococcus*, while HC had higher proportions of *Prevotella, Alistipes, Parabacteroides, Lachnospira, Agathobacter, UCG-002, and [Eubacterium]_coprostanoligenes_group* (all *P* < 0.05; **Figure 3a**). The LEfSe analysis confirmed the enrichment of *p:Proteobacteria, o:Enterobacterales, g:Klebsiella*, *f:Streptococcaceae* in the LC group, suggesting a disrupted balance of gut flora in patients with HBV-related cirrhosis (**Figure 3b**).

**Figure 4:**
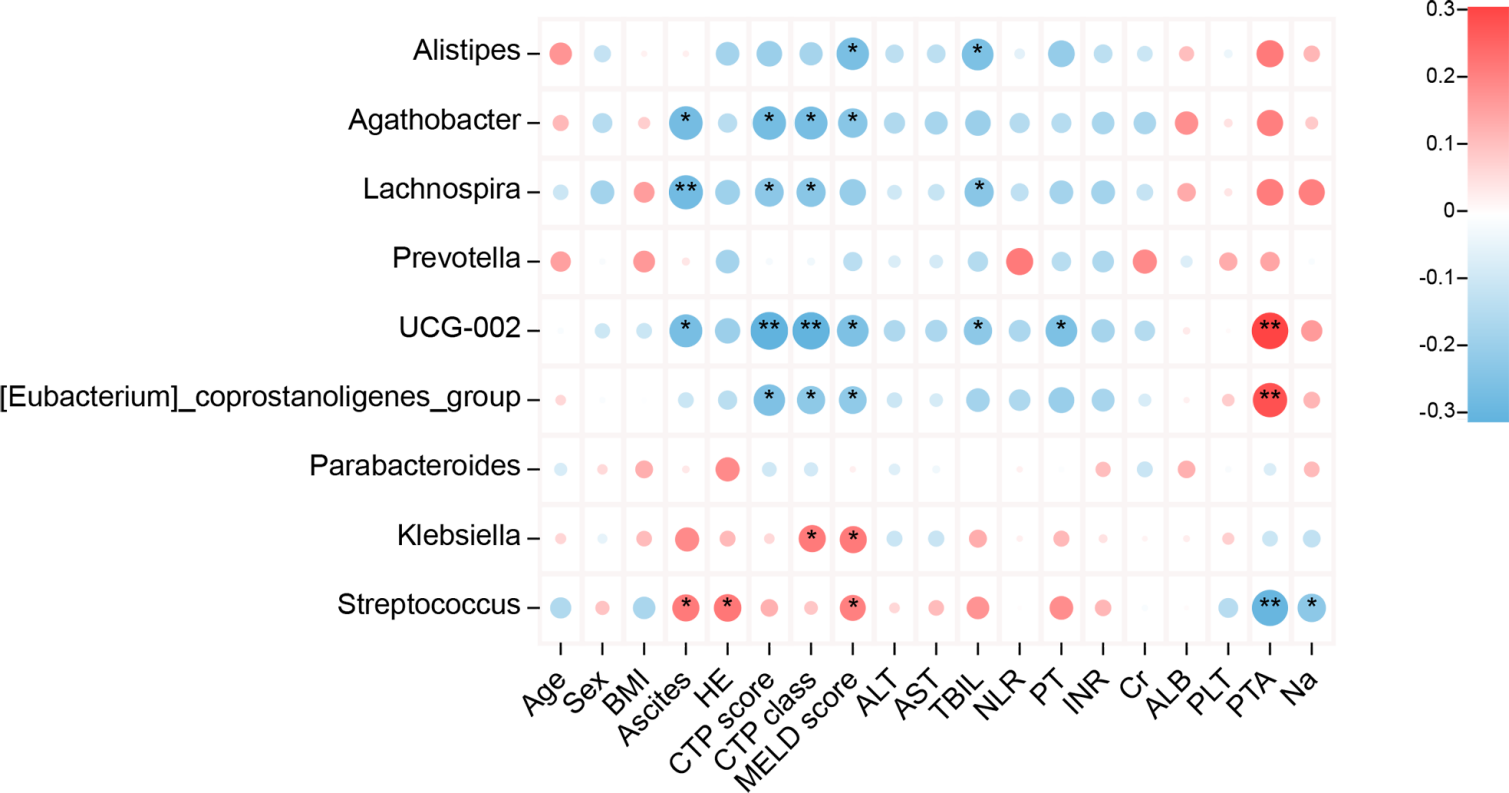
Correlation between bacterial genera abundance and clinical parameters in patients with HBV-related liver cirrhosis. Heatmaps display Spearman’s correlations between bacterial genera and clinical parameters. Red indicates positive correlations, while blue represents negative correlations (*P < 0.05, **P < 0.01).

Heatmap analysis was conducted to explore the relationship between GM and clinical indicators in patients with HBV-related cirrhosis (**Figure 4**). The most significant correlations were observed with disease severity indicators, including CTP grade, MELD score, TBIL, albumin, and prothrombin time (INR). The abundance of *Alistipes* and *Lachnospira* showed negative correlations with MELD score, CTP grade, TBIL, and INR, while *Streptococcus* and *Klebsiella* showed positive correlations with these parameters.

### Dysbiosis in cirrhosis by MELD score and CTP grades

**Figure 5:**
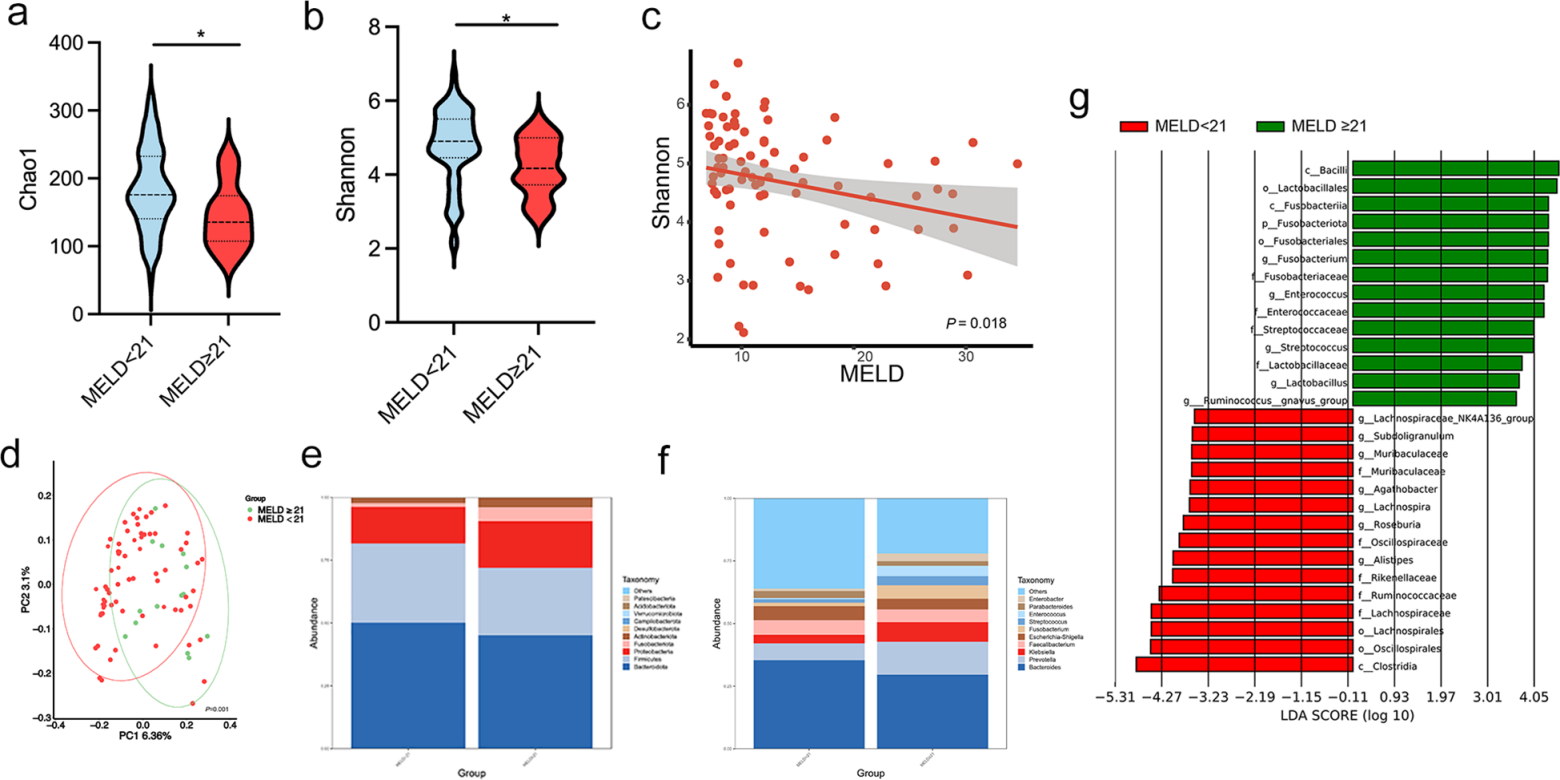
Patients with cirrhosis were grouped according to a MELD score lower or higher/equal to 21. **(a, b)** Chao 1 and Shannon indices in patients with MELD < 21 and ≥ 21; **(c)** Negative correlation between the Shannon index and MELD scores using Spearman’s analysis; **(d)** PCoA plot based on weighted UniFrac distances, demonstrating significant clustering differences between the two groups (permutation test, *P* = 0.001); **(e, f)** Relative abundance of gut microbiota at the phylum and genus levels; **(g)** LDA identifying microbial communities with scores > 4.0. LDA, linear discriminant analysis; MELD: Model for End-Stage Liver Disease. *P < 0.05.

To further investigate the association between microbiota changes and disease severity, we grouped patients based on MELD score (< 21, n = 68; ≥ 21, n = 15), a common cutoff for cirrhosis severity. Significant differences in microbial diversity were found between the two groups, as indicated by the Chao1 and Shannon indices (both *P* < 0.05; **Figures 5a, b**). Additionally, a negative correlation was observed between the Shannon index and MELD score (*P* = 0.018; **Figure 5c**). PCoA based on weighted UniFrac distances revealed a significant separation of gut microbiota profiles between the two experimental groups (*P* = 0.001, **Figure 5d**). As shown in **Figures 5e, f** the relative abundance of the gut microbiota community is presented at the genus and species levels. Beta diversity analysis revealed distinct clustering of patients based on MELD score. At the genus level, *Fusobacterium, Streptococcus*, and *Enterococcus* had significantly higher abundances in patients with MELD ≥ 21 (all *P* < 0.05). LEfSe analysis with an LDA score > 4 showed an enrichment of *c:Fusobacteriia, g:Enterococcus, and f:Streptococcaceae* in these patients. In contrast, *c:Clostridia, o:Oscillospirales, o:Lachnospirales, f:Lachnospiraceae*, and *f:Ruminococcaceae* were more abundant in patients with MELD < 21 (*P* < 0.05; LDA > 4; **Figure 5g**).

**Figure 6:**
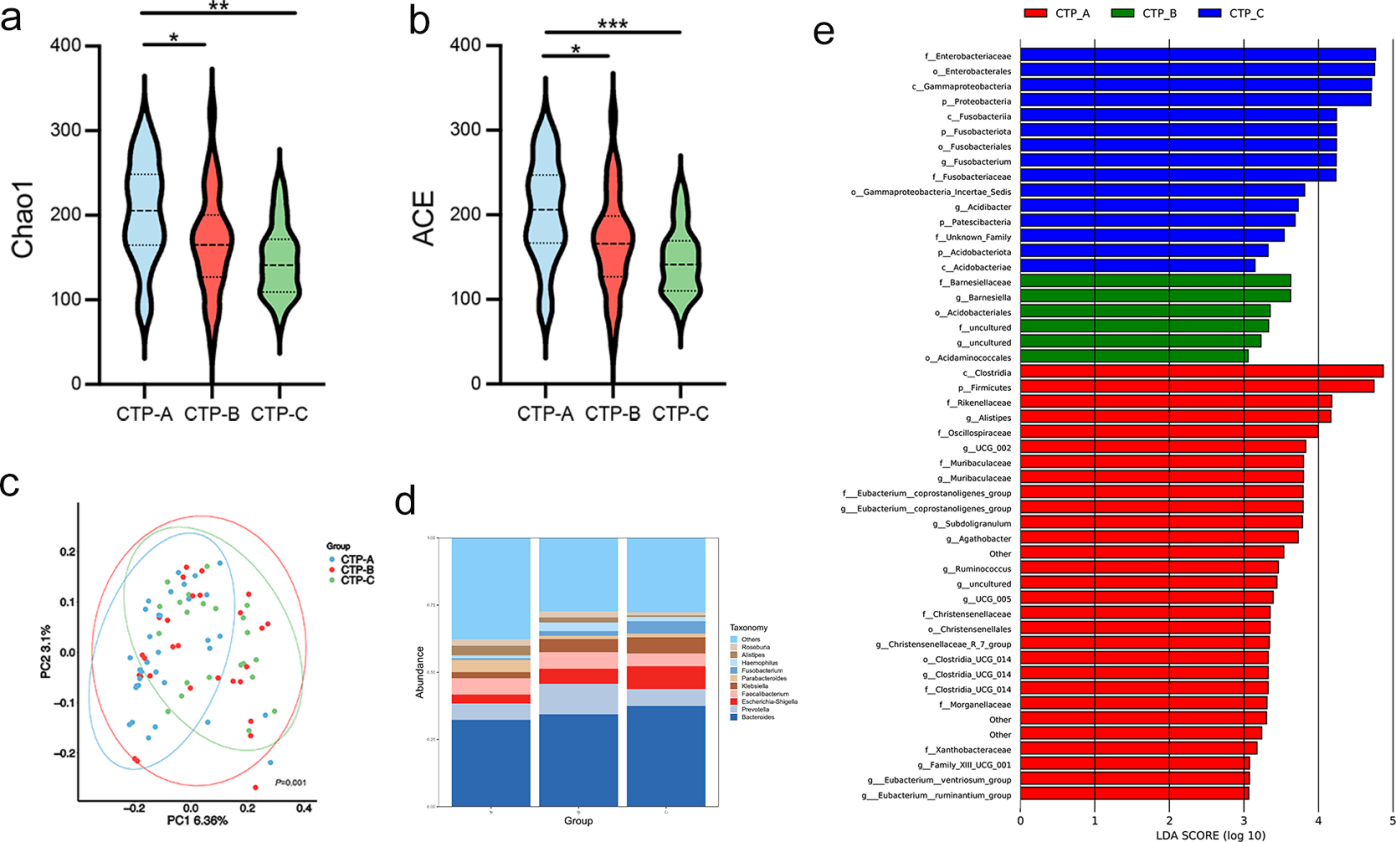
Association of CTP grades with gut microbiota. **(a, b)** Chao 1 and ACE diversity indices across CTP grades A, B, and C. **(c)** PCoA plot based on weighted UniFrac distances showing clustering differences among the three grades (permutation test, *P* = 0.002); **(d)** Genus-level differences in microbial abundance; **(e)** LDA identifying microbial communities with scores > 3.0. ACE, abundance-based coverage estimator; CTP: Child-Turcotte-Pugh; LDA, linear discriminant analysis. *P < 0.05, **P < 0.01, ***P < 0.001.

We also categorized LC patients into CTP grades A (n = 31), B (n = 30), and C (n = 22) to examine microbiota alterations across different stages of cirrhosis. The Chao1 and ACE indices indicated reduced bacterial diversity in the more advanced CTP grades (both *P* < 0.05; **Figures 6a, b**). Beta diversity analysis revealed distinct separations between these three groups (**Figure 6c**). The relative abundance of the gut microbiota community is presented at the genus level (**Figure 6d**). Notably, patients with CTP grade C had an increased abundance of *Escherichia-Shigella* and *Fusobacterium*, along with a decreased abundance of *Alistipes* and *Roseburia*. LEfSe analysis further highlighted the higher abundance of *f:Enterobacteriaceae, p:Proteobacteria*, and *c:Fusobacteriia* in CTP grade C patients (**Figure 6e**).

### Relationship between GM and cytokines/chemokines levels

**Figure 7:**
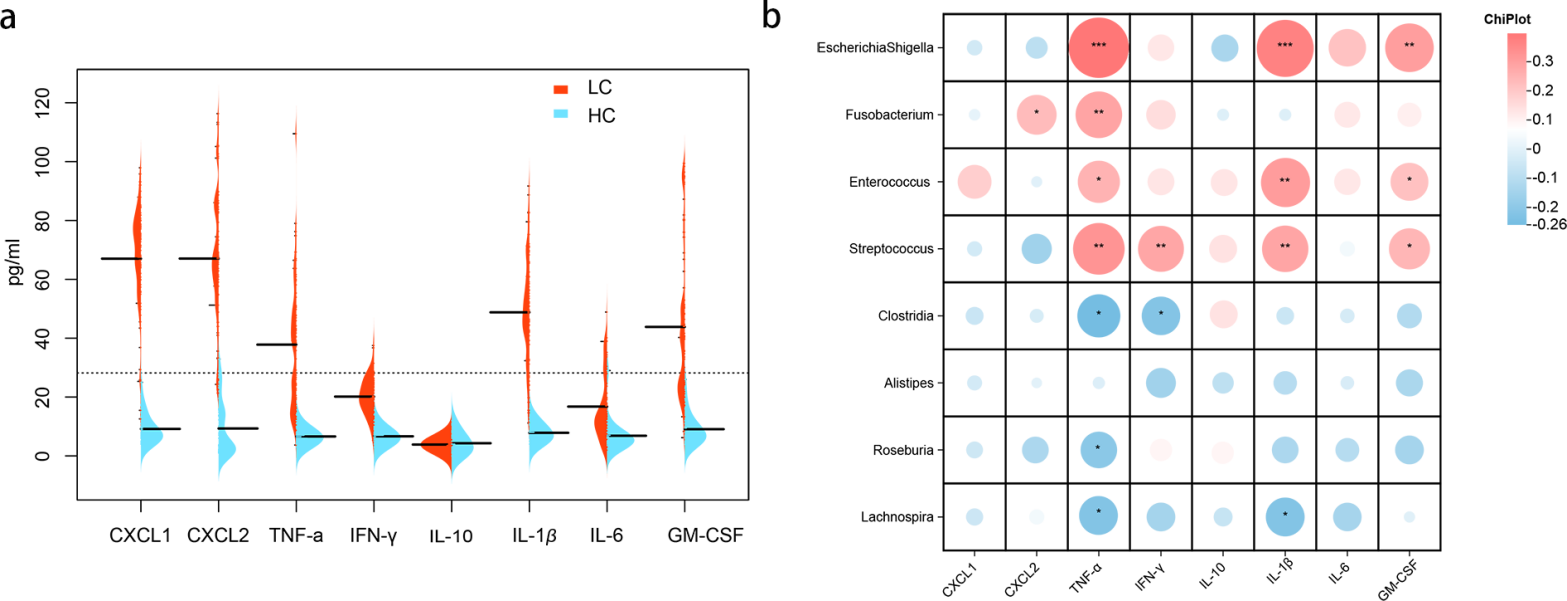
Cytokine/Chemokine expression and correlation with gut microbiota. **(a)** Cytokine/chemokine expression levels in healthy controls and patients with HBV-related cirrhosis. **(b)** Correlation analysis between intestinal bacteria and cytokine/chemokine levels in patients with cirrhosis. Heatmaps depict Spearman’s correlations, with red indicating positive correlations and blue negative correlations (*P < 0.05; **P < 0.01; ***P < 0.001).

To explore the correlation between GM abundance and the inflammatory state, we measured plasma levels of cytokines and chemokines in both HC and LC groups. Higher levels of CXCL1, CXCL2, TNF-α, IFN-γ, IL-1β, IL-6, IL-10, and GM-CSF were observed in the LC group (**Figure 7a**). Several correlations were found between GM and cytokine/chemokine levels. *Escherichia-Shigella, Fusobacterium, Enterococcus*, and *Streptococcus* were positively correlated with TNF-α, while *Clostridia, Roseburia*, and *Lachnospira* were negatively correlated with TNF-α. Additionally, *Escherichia-Shigella*, and *Enterococcus* were positively correlated with IL-1β and GM-CSF, and *Streptococcus* showed positive correlations with IFN-γ, IL-1β, and GM-CSF. Conversely, *Clostridia* exhibited negative correlations with IFN-γ, and *Lachnospira* was negatively correlated with IL-1β (all *P* < 0.05; **Figure 7b**).

### Characterization of metabolites from stool of patients with HBV-LC

**Figure 8:**
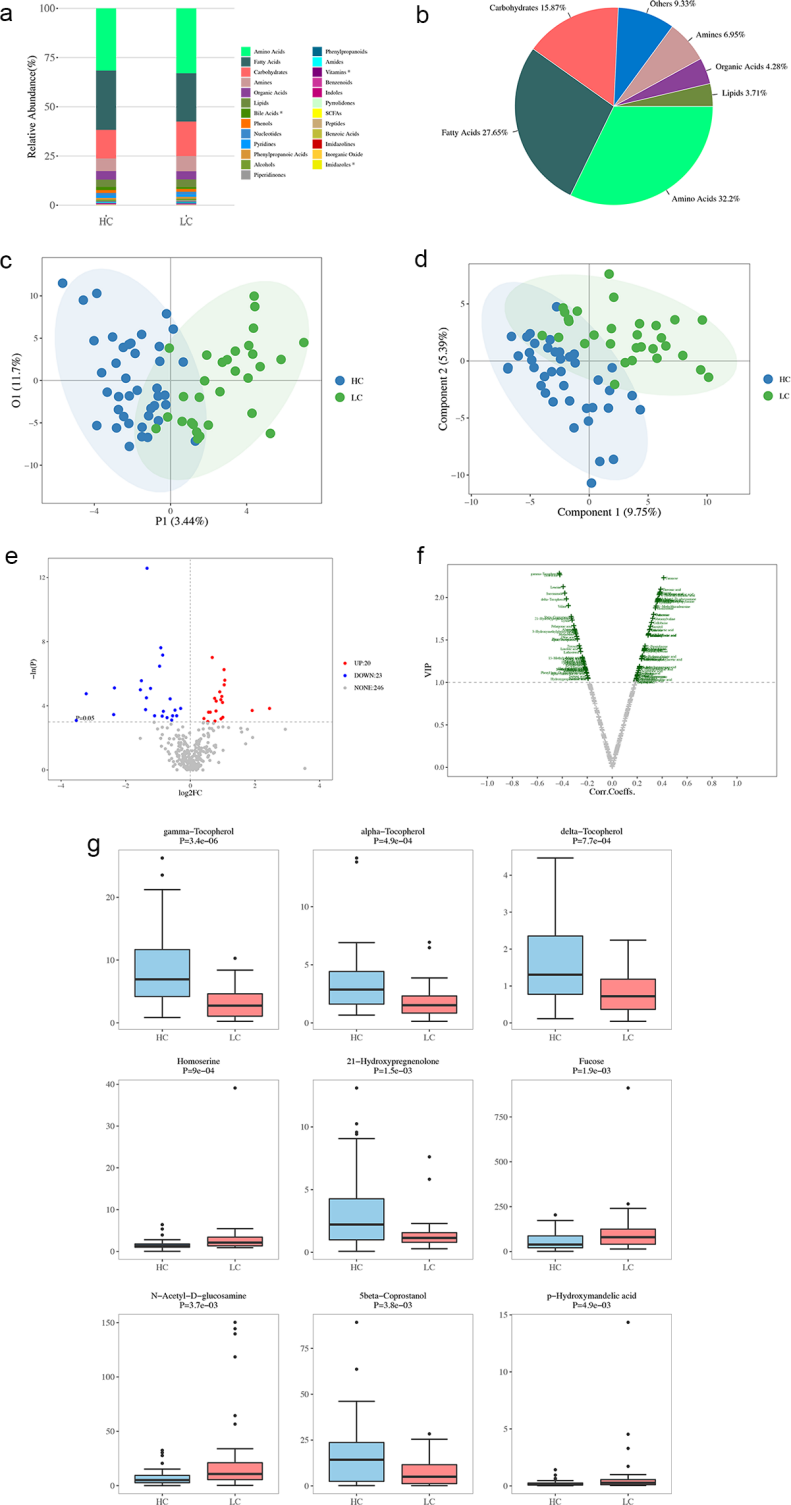
Metabolite Profiling in healthy controls (HC) and patients with HBV-related cirrhosis (LC). **(a)** Distribution of metabolites in LC and HC groups. **(b)** Categorical and quantitative metabolite distributions across samples. **(c)** Individual plot of PCA between HC and LC groups. **(d)** OPLS-DA scores plot of metabolites from HC and LC groups. **(e)** Univariate statistical analysis of differential metabolites: red dots indicate upregulated metabolites in the LC group, and blue dots represent downregulated ones. **(f)** Multivariate statistical analysis of differential metabolites. The x-axis shows Pearson’s correlation coefficients, and the y-axis represents metabolite contributions to model discrimination. Green dots indicate metabolites with VIP scores > 1. **(g)** Boxplots of the top nine differential metabolites.

The influence of the GM on disease is often mediated by its metabolites. Understanding the characteristics and risk factors of patients with CTP grade A cirrhosis can aid in the development of effective prevention strategies to reduce the occurrence and progression of cirrhosis. Therefore, a metabolomic analysis of 71 samples (40 healthy controls and 31 CTP grade A patients) was performed. Significant differences in metabolites related to bile acids, vitamins, and imidazoles were observed between healthy individuals and patients with CTP grade A cirrhosis (*P* < 0.05; **Figure 8a**). The identified metabolites were mainly classified as follows: amino acids (32.2%), fatty acids (27.6%), carbohydrates (15.8%), amines (6.9%), organic acids (4.2%), lipids (3.7%), and other classes (9.3%) (**Figure 8b**).

The PLS-DA analysis (**Figure 8c**) illustrates the distributional differences in metabolic characteristics between healthy controls and cirrhosis patients. The OPLS-DA analysis (**Figure 8d**) further refines group differentiation by eliminating intra-group variability, providing a clearer distinction in metabolic patterns between the two groups. The volcano plot presents the differential metabolites identified through one-dimensional statistical analysis, with the threshold set at an absolute log2Fold Change value of ≥ 0 and a *P*-value of < 0.05. In the LC group, 20 metabolites located in the upper right corner were found to be increased, while 22 metabolites in the upper left corner were reduced, when compared to the HC group (**Figure 8e**). This analysis, based on the OPLS-DA model results, utilized the volcano plot to identify reliable metabolic markers. It provided a comprehensive evaluation of the metabolites’ contributions to the model grouping (VIP) and their overall reliability. A total of 86 metabolites with significant differences were detected (**Figure 8f**). Our analysis of the top nine most significant differential metabolites revealed that the LC group had higher intestinal concentrations of certain amino acids (homoserine), fucose, N-acetyl-D-glucosamine, and p-hydroxymandelic acid, as well as lower concentrations of certain amino acids (gamma-tocopherol, alpha-tocopherol, delta-tocopherol), 21-hydroxypregnenolone, and 5-beta coprostanol compared to the HC group (**Figure 8g**).

### Metabolic pathway and gut microbiota-metabolite correlation

**Figure 9:**
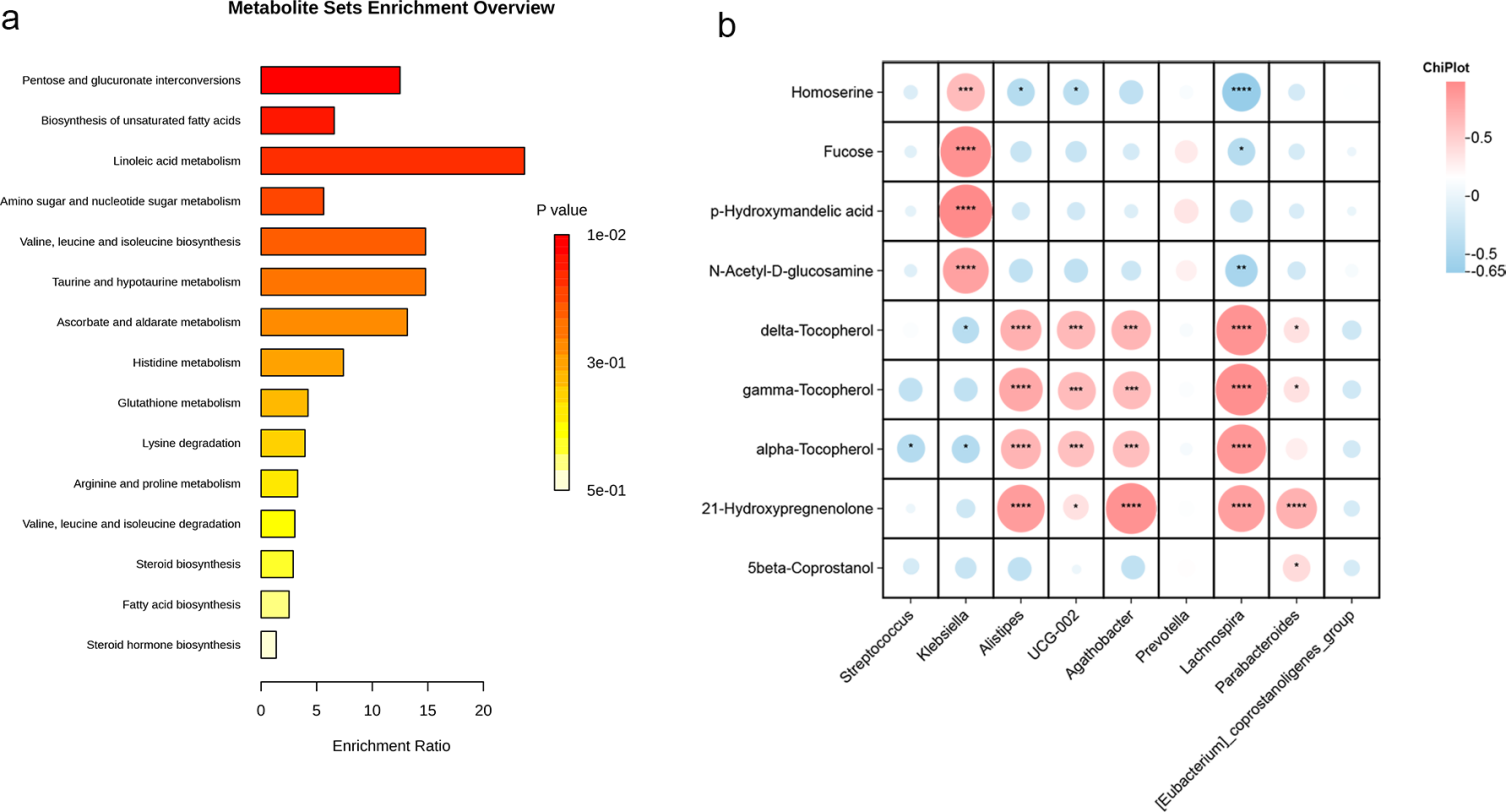
Metabolic pathway enrichment and correlations between gut microbiota and metabolites. **(a)** Pathway enrichment analysis of predicted metabolite sets, with bar lengths indicating fold enrichment and color representing p-values. **(b)** Correlation analysis between differential bacterial genera and metabolites, where red indicates positive correlations and blue negative correlations (*P < 0.05; **P < 0.01; ***P < 0.001; ****P < 0.0001,).

We conducted an enrichment pathway analysis for the differential metabolites, which revealed significant enrichment in pathways such as pentose and glucuronate interconversions, biosynthesis of unsaturated fatty acids, linoleic acid metabolism, and amino sugar and nucleotide sugar metabolism. Among these, linoleic acid metabolism emerged as the most influential metabolic pathway (**Figure 9a**).

To gain a deeper understanding of the interaction between GM and metabolites in early cirrhosis, we performed a correlation analysis between the major differential microorganisms and metabolites (**Figure 9b**). The results showed a significant positive correlation between the abundances of *Alistipes, Agathobacter, Lachnospira*, and *Parabacteroides* with Tocopherol and 21-hydroxypregnenolone. Additionally, *Klebsiella* showed a positive correlation with N-Acetyl-D-glucosamine, p-Hydroxymandelic acid, fucose, and homoserine, while *Lachnospira* exhibited a negative correlation with these metabolites.

## Discussion

The prognosis of patients with HBV-related cirrhosis varies significantly, with severe cirrhosis being particularly associated with high mortality rates (Lan et al., 2024). Microorganisms and their metabolites play a crucial role in cirrhosis progression, as decompensation events are linked to the GM and their interaction through the gut–liver axis (Acharya and Bajaj, 2019). Identifying specific GM and metabolites could serve as early warning biomarkers for cirrhosis and help in early identification and management of patients with cirrhosis, thereby improving patient outcomes. In our study, we identified significant dysbiosis in the GM of patients with HBV-related cirrhosis, with these alterations correlating with clinical features, inflammatory factors, and metabolites. These findings offer new insights into the role of GM and metabolites in the pathogenesis of HBV-related cirrhosis, highlighting their potential as targets for novel diagnostic and therapeutic strategies.

Significant differences in the intestinal flora were observed between patients with HBV-related cirrhosis and those with heathly controls. In the LC group, the relative abundance of *Klebsiella* and *Streptococcus* was elevated, while the abundance of butyrate-producing bacteria such as *Alistipes*, *Parabacteroides*, and *Lachnospira* was significantly reduced (all *P* < 0.05). Our study found significant correlations between GM composition and clinical indicators in cirrhotic patients, including CTP grade, MELD score, and levels of TBIL, ALB, and INR. These results suggest that GM alterations are closely linked to the severity of HBV-related cirrhosis.

Cirrhosis-related dysbiosis can lead to serious complications, such as small intestinal bacterial overgrowth and increased intestinal permeability (Chopyk and Grakoui, 2020). To further explore these changes, we examined the intestinal flora in patients with different MELD and CTP grades. At the genus level, *Fusobacterium* and *Streptococcus* were more abundant in patients with MELD ≥ 21. Additionally, *Fusobacterium* were significantly more prevalent in patients with CTP C grade, while *Alistipes* and *Roseburia* were markedly reduced. Patients with poor liver function and higher MELD scores tend to have increased populations of LPS-producing genera. Overgrowth of these bacteria can increase intestinal permeability, promote bacterial translocation, elevate LPS levels, and trigger systemic inflammation, leading to acute decompensation events (Chen et al., 2018; Liu et al., 2019). Conversely, lower levels of *Alistipes* and *Roseburia*, which produce short-chain fatty acids, have been associated with poorer outcomes. These bacteria play a critical role in reducing inflammation, improving endotoxin levels, enhancing intestinal barrier function, and modulating systemic immunity (Parker et al., 2020; Matar et al., 2024; Wang et al., 2024).

Systemic inflammation is a key factor in cirrhosis progression and is a major driver of immune dysfunction in cirrhosis complications. Microbial imbalance impacts liver health through the enterohepatic axis, further exacerbating inflammation and immune responses (Leuti et al., 2020). In cirrhotic patients, impaired intestinal barrier function leads to the translocation of large amounts of LPS to the liver, where it binds to pattern recognition receptors like toll-like receptors (TLRs) and nucleotide-binding oligomerization domain-like receptors (NLRs), activating liver immune cells, particularly Kupffer cells. This triggers the release of pro-inflammatory cytokines such as TNF-α and IL-6 (Liu and Yang, 2023). Our analysis of plasma cytokine levels and GM changes found that *Streptococcus* was positively correlated with TNF-α, IFN-γ, and IL-1β, while *Clostridia, Roseburia*, and *Lachnospira* showed negative correlations with TNF-α. These findings suggest that the GM and inflammatory factors are closely linked in cirrhotic patients, and that modulating GM composition could provide a novel approach to reducing inflammation and improving liver function.

Although patients with CTP grade A typically have better liver function, they can still progress to advanced cirrhosis. By exploring metabolism-related markers, we can identify early risks of liver failure and guide clinical decision-making. The correlation analysis revealed that tocopherols and 21-hydroxypregnenolone were positively associated with *Alistipes* and *Lachnospira*. Tocopherols (including the gamma, alpha, and delta forms) are antioxidants, and reduced levels may indicate increased oxidative stress, and supplementation with tocopherols promotes cirrhosis regeneration (Andiran et al., 2000). Since the liver is a major organ for vitamin E metabolism, impaired liver function can affect tocopherol levels. 21-hydroxypregnenolone is a steroid involved in liver metabolism and steroid hormone synthesis, and it plays a role in the regulation of iron-induced cell death by controlling oxidative stress. Recent studies suggest that 17 alpha-hydroxypregnenolone, a steroid hormone, can inhibit liver injury caused by iron death (Lin et al., 2024).

Pathway enrichment analysis revealed significant alterations in the biosynthesis of unsaturated fatty acids and linoleic acid metabolism. Disruption of lipid metabolism, particularly in unsaturated fatty acids like linoleic acid, is a hallmark of liver cirrhosis and may be linked to liver fat accumulation and chronic inflammation (López-Vicario et al., 2020; Clària et al., 2021). Impaired liver cell function may further exacerbate these metabolic disturbances. We hypothesize that increased abundance of *Alistipes* positively influences intestinal homeostasis, reduces hepatic accumulation of linolenic acid metabolites, and improves unsaturated fatty acid metabolism, though the exact mechanism remains unclear. Nutritional supplementation, particularly with foods or supplements rich in alpha-linolenic acid, may offer a promising strategy for improving intestinal and metabolic health in cirrhotic patients.

This study provides a comprehensive analysis of GM characteristics in patients with HBV-associated cirrhosis from North China, highlighting bacterial genera, inflammatory factors, and metabolites, and offering potential preventive strategies. However, there are some limitations. First, our study was conducted at a single center with a limited sample size, and further research involving multi-center prospective cohorts is needed to validate our findings and draw more robust conclusions. Additionally, the cross-sectional design limits our ability to infer causality between GM changes and disease progression. Longitudinal studies are needed to establish temporal relationships and clarify whether alterations in GM contribute to disease onset or progression, or if they are merely a consequence of the disease. Third, the study primarily focused on 16S rRNA sequencing for microbiota profiling, which, while informative, does not capture the full functional potential of the microbiota. Future studies incorporating metagenomic and metabolomic profiling could provide deeper insights into the microbial functions and metabolic pathways involved in HBV-related cirrhosis. Finally, while associations between intestinal flora and metabolites were identified, further research using animal models and human studies is needed to elucidate the pathogenic mechanisms and identify effective targets for preventing cirrhosis progression.

## Conclusion

This study revealed significant differences in the GM between patients with HBV-related cirrhosis and healthy controls, with increased *Klebsiella* and *Streptococcus*, and decreased *Alistipes, Lachnospira*, and *Agathobacter.* These changes correlated with disease severity (MELD, CTP scores) and inflammatory markers. Additionally, decreased levels of tocopherols and 21-hydroxypregnenolone were linked to specific gut bacteria. However, the cross-sectional design limits causal inferences. Future research should focus on longitudinal studies, larger cohorts, and integrated metagenomic and metabolomic analyses to confirm and expand these findings.

## Data Availability

The data presented in the study are deposited in the National Center for Biotechnology Information (NCBI) repository, accession number PRJNA1259947.
