# Supplementary material for: Distinct gut microbiota and metabolomic profiles in HBV-related liver cirrhosis: insights into disease progression
Source: Front Cell Infect Microbiol. 2025 May 19;15:1560564. doi: 10.3389/fcimb.2025.1560564 (PMC12127420; doi:10.3389/fcimb.2025.1560564)
Supplement: Supplementary file 1 [file DataSheet1.pdf]

Taxonomy	HC20	HC21	HC22	HC23	HC24	HC25	HC26	HC27
Bacteroides	16734	10900	35127	25656	30807	27446	7847	2563
Prevotella	211	1670	122	132	162	200	28963	38222
Faecalibact	7730	1370	1749	1516	1097	4399	551	81
Escherichia	332	3028	987	900	308	462	969	265
Klebsiella	37	76	779	89	0	19	0	59
Alistipes	393	3042	654	2180	6966	0	3178	27
Parabacter	173	1878	2147	1227	1595	1197	1875	41
Other	1537	2053	1674	902	428	476	462	127
Roseburia	451	1633	588	212	436	71	278	16
Bifidobacte	173	769	101	102	51	1322	118	17
Fusobacteri	15	21	40	11	21	223	13	4930
Haemophil	798	59	68	77	94	44	19	35
Streptococc	20	18	207	15	38	12	19	14
Lachnoclos	559	77	1364	271	92	144	395	972
[Ruminococ	29	39	36	438	16	51	100	108
Blautia	122	246	1035	158	256	830	488	9
Enterobact	19	9556	119	18	34	0	0	0
Agathobact	674	550	84	975	396	1970	79	23
Lachnospir	2096	177	349	1170	135	154	185	106
UCG-002	157	2139	450	247	776	0	1208	6
[Eubacteriu	352	5401	362	283	2108	388	1315	6
Subdoligrar	441	86	4552	269	529	193	659	8
Parasuttere	465	54	32	489	104	10	200	7
Enterococc	46	28	10	42	21	0	0	0
Lachnospir	681	162	270	33	1254	265	42	0
Barnesiella	316	311	6	0	3555	0	654	0
Muribaculo	22	1409	68	22	62	17	20	7
Sutterella	37	123	102	10	0	9	7	4170
Dialister	13	7	0	57	0	678	0	568
Ruminococ	273	313	599	27	37	2492	1545	5
[Ruminococ	139	168	106	0	82	221	648	52
Collinsella	68	118	161	0	11	0	47	56
[Eubacteriu	15	439	0	8	219	0	0	0
CAG-352	57	115	4	0	78	8	0	9
Lactobacill	0	2	14	5	18	0	31	0
uncultured	168	1197	245	92	689	180	181	0
Coprococcu	202	321	62	69	419	47	101	9
Christensen	50	1881	217	186	65	83	52	0
Fusicatenib	412	139	51	262	62	529	30	0
Anaerostipe	17	28	114	19	32	212	29	0
Odoribacte	44	995	4	432	1177	229	197	0
Clostridia_	31	1926	6	12	1426	0	0	0

UCG-005	38	786	50	6	391	189	0	0
Paraprevot	306	0	0	201	2004	0	376	0
Dorea	33	22	347	55	67	126	161	63
[Eubacteriu	35	43	211	67	0	203	315	3
[Eubacteriu	145	0	220	39	27	29	343	0
Ralstonia	20	29	42	23	142	0	15	0
Alloprevote	7	0	0	0	0	0	3	0
Coprobacte	87	0	3	0	1013	2	30	0
Erysipelotri	203	14	110	0	76	63	258	10
Butyricimo	65	486	0	0	551	0	188	0
Lachnospir	133	33	124	49	6	59	0	16
Erysipelato	0	0	0	57	0	0	0	0
Butyricicoc	23	26	259	0	24	61	0	0
Clostridium	9	50	5	7	18	9	0	4
Monoglobu	86	61	205	18	132	0	259	0
[Eubacteriu	365	102	0	0	0	0	492	0
Lachnospir	50	12	1507	6	8	112	0	7
Romboutsic	26	12	110	0	28	0	0	18
Bilophila	54	34	3	62	15	0	88	0
Lachnospir	125	141	28	30	5	45	48	0
Flavonifrac	0	0	16	10	0	73	0	0
NK4A214_	15	106	0	0	207	0	157	0
Hungatella	0	10	15	0	0	0	0	0
Lachnospir	39	70	15	0	58	412	0	0
Proteus	0	0	0	11	0	0	0	0
[Clostridiur	0	4	0	10	0	7	0	0
Tyzzerella	8	4	40	0	0	265	0	0
UBA1819	4	10	95	8	9	44	22	0
CAG-56	0	0	64	0	29	0	18	0
Staphylococ	0	0	0	4	0	0	0	0
UCG-003	70	64	30	321	53	13	125	0
Cetobacteri	0	0	0	0	2	0	0	0
Eggerthella	0	0	0	23	0	65	3	0
Rothia	4	8	0	10	13	5	6	0
Clostridioid	0	0	0	0	0	0	0	0
Citrobacter	0	0	96	0	0	0	0	0
Incertae_Se	18	3	44	11	8	20	28	0
Oscillibact	14	22	59	15	13	58	14	0
Sphingomo	0	16	22	0	20	0	10	0
Turicibacte	0	7	0	0	14	0	0	0
Colidextrib	8	0	264	54	8	282	0	0
Aggregatib	12	0	9	13	9	0	0	0
Actinomyce	21	20	12	15	27	16	9	23

Mitsuokella	0	0	50	0	0	0	0	0
Desulfovibı	114	110	0	0	0	0	0	0
[Eubacteriu	0	92	162	0	88	0	0	0
[Eubacteriu	82	42	0	0	0	0	0	0
Rikenellace	0	0	0	0	0	0	0	0
Intestinibac	0	19	0	34	17	23	7	0
Holdemane	0	0	0	0	0	0	14	0
Neisseria	76	35	46	50	66	28	34	16
UCG-010	0	491	0	0	26	0	0	0
Sediminiba	0	0	7	0	19	3	0	0
Akkermansi	0	0	0	0	0	0	0	0
Lautropia	22	13	27	0	21	11	6	13
Megamonas	9	0	0	0	0	0	16	79
Family_XII	0	47	42	5	0	0	20	0
Adlercreutz	0	16	0	0	0	0	0	0
Clostridia_	0	639	0	0	0	0	11	0
Allisonella	0	0	3	0	0	0	0	29
Pediococcus	0	0	0	0	0	0	0	0
Granulicat	0	0	12	0	15	0	0	0
Morganella	0	0	0	0	0	0	0	0
Veillonella	0	0	0	0	0	0	0	0
Catenibact	0	0	0	0	0	0	0	0
RF39	0	30	94	0	66	0	0	0
Anaerotrun	0	10	17	0	0	0	0	0
Weissella	0	0	0	0	0	0	0	0
[Ruminococ	33	0	0	22	0	0	40	0
Capnocytop	31	32	29	43	22	9	18	0
Faecalitale	0	0	7	0	0	0	0	0
Leptotrichi	0	0	0	8	7	0	0	0
Rikenella	0	0	4	0	0	0	0	0
Senegalima	8	0	0	0	0	0	0	0
Campyloba	0	0	0	0	4	0	0	0
Terrisporob	0	0	0	0	22	4	0	0
Prevotellac	0	0	0	0	0	0	0	0
Prevotellac	0	0	0	0	0	6	4	6
GCA-90006	27	0	0	26	9	0	24	0
Sellimonas	0	0	0	0	0	0	0	0
Megasphae	0	0	0	0	0	0	0	3
Erysipelotri	0	0	0	0	0	0	0	0
Coprobacil	0	0	0	0	0	0	0	0
Stenotroph	0	0	0	0	0	0	0	0
Lachnospir	0	0	13	0	0	0	37	0
Raoultella	14	0	11	0	0	0	0	0

Slackia	0	0	0	0	0	53	7	0
Marvinbryo	0	0	0	0	0	6	20	0
Intestinimo	0	0	20	0	0	0	6	0
Gemella	0	0	0	0	0	0	0	0
Olsenella	0	0	0	0	0	0	5	0
Abiotrophi	0	0	0	0	0	0	0	0
Negativiba	0	5	0	0	0	0	0	0
Lachnospir	0	0	0	0	0	0	0	0
Corynebact	0	9	0	0	12	0	0	18
Eisenbergie	0	0	0	0	0	0	0	0
Fournierell	0	71	0	0	0	0	0	0
Phascolarct	0	0	7	0	0	0	9	0
Howardella	0	0	0	0	0	0	0	0
Atopobium	0	0	0	0	0	0	0	0
[Bacteroide	0	0	0	0	0	0	0	0
Peptococcu	12	18	0	0	17	0	0	0
Peptostrept	0	0	0	0	0	0	0	0
Lactococcu	0	0	24	0	0	0	0	0
Family_XII	3	0	0	4	0	7	7	0
Holdemani	0	4	15	0	0	3	0	0
Porphyrom	11	4	4	3	8	6	0	0
Prevotellac	0	0	0	0	0	0	0	0
Helicobacte	0	0	4	0	0	0	0	0
Bradyrhizol	0	0	0	0	0	0	0	0
Robinsonie	0	0	0	0	0	0	0	0
Cardiobact	3	5	3	0	7	2	0	8
Gordoniba	0	0	0	0	6	0	0	0
[Eubacteriu	0	0	0	0	0	0	0	0
Oxalobacte	0	13	0	0	0	0	0	0
Dysgonomo	0	0	0	0	0	0	0	0
Dielma	0	0	0	0	0	0	0	0
Eikenella	0	0	0	0	0	0	0	7
UCG-009	0	17	0	2	0	0	0	0
Shuttlewort	0	0	0	0	0	0	0	0
Ruminiclos	0	14	0	0	0	0	0	0
Paenibacill	0	0	0	0	0	0	0	0
Arenibacte	0	0	0	0	0	0	0	0
[Eubacteriu	0	0	23	0	0	0	0	0
Oscillospir	0	0	5	0	0	0	0	0
[Eubacteriu	0	0	0	0	0	0	5	0
Sarcina	0	0	0	0	0	0	0	0
Anaerofilu	0	0	0	0	0	0	15	0
Frisingicoc	0	0	0	0	0	0	0	0

Comamona	21	20	17	22	20	0	0	0
Izemoplasm	0	16	0	0	0	0	0	0
Solobacteri	0	0	0	0	0	0	0	0
Tuzzerella	0	13	0	0	0	0	0	0
Mogibacter	0	0	0	0	0	0	0	0
Kluyvera	0	0	0	0	0	0	0	0
Allorhizobi	0	0	0	0	0	0	5	0
Oribacteriu	0	0	0	0	0	0	0	0
Methylobac	0	0	0	0	0	0	0	0
Christensen	0	0	0	0	0	0	0	0
Enterorhab	0	0	0	0	5	0	0	0
Salmonella	0	0	0	0	0	0	0	0
Phocea	0	0	11	0	0	5	0	0
Aeromonas	0	0	0	0	0	0	0	0
Gastranaer	0	0	0	0	26	0	0	0
Acidovorax	0	0	0	0	0	0	0	0
Raoultibact	0	0	0	0	0	0	0	0
Anaerostigr	0	0	0	0	0	0	0	0
UCG-004	0	88	0	0	0	0	0	0
Mucispirill	0	0	0	0	0	0	2	0
Cronobacte	0	0	0	0	0	0	0	0
Lactonifact	0	0	0	0	0	0	0	0
Sanguibact	0	47	0	0	0	0	0	0
Paludicola	0	0	0	0	0	0	0	0
Alloscardov	0	0	0	0	0	0	0	0
Parvimonas	0	0	0	0	0	0	0	0
Limnohabit	0	0	0	0	0	0	0	0
Candidatus	0	0	0	0	0	0	0	0
Hafnia-Ob	0	0	0	0	0	0	0	0
F0058	8	0	0	7	0	0	0	5
Actinobacil	0	0	0	0	0	0	0	0
Fenollaria	0	0	0	0	0	0	0	0
Succinivibr	0	0	0	0	0	0	0	0
Serratia	0	0	0	0	0	0	0	0
CL500-29_r	0	0	0	0	0	0	0	0
Acidaminoc	0	0	0	0	0	0	0	0
[Clostridiur	0	0	0	0	0	0	0	0
Lachnospir	0	0	0	0	0	0	0	0
Lachnospir	0	0	0	0	0	30	0	0
Aquabacter	0	0	0	0	0	0	0	0
Catabacter	0	0	0	0	0	0	0	0
Herbaspiril	0	0	0	0	14	0	0	0
DTU089	0	0	0	0	0	0	3	0

Flavobacte[illegible]	0	0	0	0	0	0	0	0
Muribaculu	0	0	0	0	0	0	0	0
Hydrogenoc	0	14	0	0	0	0	0	0
Eubacteriu	0	0	0	0	0	0	0	0
Acinetobac	0	0	0	0	0	0	0	0
Bacillus	0	0	0	0	0	0	0	0
Sporichthyo	0	0	0	0	0	0	0	0
Moryella	0	0	0	0	0	0	13	0
Sneathia	0	0	0	0	0	0	0	0
Faecalicocc	0	0	0	0	0	0	0	0
Rodentibac	0	0	0	0	0	0	0	0
Aureimona	0	0	0	0	0	0	0	0
Candidatus	0	0	0	0	0	0	0	0
F0332	0	0	0	4	0	0	0	0
Providencio	0	0	0	0	0	0	0	0
Anaeroplas	0	0	0	0	0	0	0	0
Muribacter	0	0	0	0	0	0	0	0
Scardovia	0	0	0	0	0	0	0	0
Dongia	0	0	0	0	4	0	0	0
Dubosiella	0	0	0	0	0	0	0	0
Pseudarthro	0	0	0	0	0	0	0	0
Pseudomon	0	0	0	0	0	0	0	0
Acetanaero	0	0	0	0	0	0	0	0
Johnsonella	0	0	0	0	0	0	0	0
GCA-90006	0	0	13	5	0	0	0	0
Paraeggertl	0	0	0	0	0	0	0	0
hgcI_clade	0	0	0	0	0	0	0	0
Lachnospir	0	18	0	0	0	0	0	0
Lysobacter	0	0	0	0	0	0	0	0
Defluviitale	0	0	0	0	0	0	0	0
Burkholderi	0	0	0	0	0	0	0	0
Kribbella	0	0	0	0	0	0	0	0
Victivallis	0	15	0	0	0	0	0	0
Gaiella	0	0	0	0	0	0	0	0
Angelakisel	0	0	0	0	0	0	0	0
Subgroup_2	0	0	0	0	0	0	0	0
Nitrospira	0	0	0	0	0	0	0	2
Streptomyce	0	0	0	0	0	0	0	0
Phreatobac	0	0	0	0	0	0	0	0
Glutamicib	0	0	0	0	0	0	0	0
Phocaeicolo	0	0	0	0	0	0	0	0
Butyrivibrio	0	12	0	0	0	0	0	0
Brachymon	0	0	0	0	0	0	0	0

SC-I-84	0	0	0	0	0	0	0	0
Parascardo	0	0	0	0	0	0	0	0
Kingella	0	6	0	0	0	0	0	0
Delftia	0	0	0	0	0	0	0	0
Abscondita	0	0	0	0	0	0	0	0
Xanthomor	0	0	0	0	0	0	0	0
Amycolato	0	0	0	0	0	0	0	0
Epulopisciu	0	0	0	0	0	0	0	0
Acidibacter	0	0	0	0	0	0	0	0
67-14	0	0	0	0	0	0	0	0
Candidatus	0	0	0	0	0	0	0	0
Clade_Ia	0	0	0	0	0	0	0	0
Ellin6055	0	0	0	0	0	0	0	0
Luedemann	0	0	0	0	0	0	0	0
Lechevalier	0	0	0	0	0	0	0	0
ASF356	0	0	0	0	0	0	0	0
Treponema	0	0	0	0	0	0	0	0
Paeniclostr	0	0	0	0	0	0	0	0
Ellin6067	0	0	0	0	0	0	0	0
Anaerovora	0	0	0	0	0	0	0	0
Cryptobact	0	0	0	0	0	0	0	0
Lachnospir	0	0	0	0	0	0	0	0
Lachnospir	0	0	0	0	0	0	0	0
CHKCI002	0	0	0	0	0	0	0	0
Sphingobac	0	0	0	0	0	0	0	0
Clostridium	0	0	0	0	0	0	0	0
Enorma	0	0	0	0	0	0	0	0
Harryflintic	0	0	0	0	0	0	0	0
CHKCI001	0	0	0	0	0	0	0	0
Thiobacillu	0	0	0	0	0	0	0	0
Hirschia	0	0	0	0	0	0	0	0
Bryobacter	0	0	0	0	0	0	0	0
Psychrobac	0	0	0	0	0	0	0	0
Stomatobac	0	0	0	0	0	0	0	0
mle1-7	0	0	0	0	0	0	0	0
Pseudoflavo	0	0	0	0	0	0	0	0
Ochrobactr	0	0	0	0	0	0	0	0
Papillibact	0	0	0	0	0	0	0	0
Reyranella	0	0	0	0	0	0	0	0
Rhodanoba	0	0	0	0	0	0	0	0
PeM15	0	0	0	0	0	0	0	0
Clostridium	0	0	0	0	0	0	0	0
Luteimonas	5	0	0	0	0	0	0	0

Shewanella	0	0	0	0	0	0	0	0
S5-A14a	0	0	0	0	0	0	0	0
Aquicella	0	0	0	0	0	0	0	0
Polynucleol	0	0	0	0	0	0	0	0
Catenulispo	0	0	0	0	0	0	0	0
Candidatus	0	0	0	0	0	0	0	0
Rhodoplan	0	0	0	0	0	0	0	0
Acidotherm	0	0	0	0	0	0	0	0
Dechlorom	0	0	0	0	0	0	0	0
Phenylobac	0	0	0	0	0	0	0	0
Denitrobac	0	0	0	0	0	0	0	0
Aeromicrob	0	0	0	0	0	0	0	0
Gramella	0	0	0	0	0	0	0	0
Terrimonas	0	0	0	0	4	0	0	0
TM7x	0	0	0	0	0	0	0	0
Succinatim	0	0	0	0	0	0	0	0
Saccharosp	0	0	0	0	0	0	0	0
Arthrobact	0	0	0	0	0	0	0	0
Micromono	0	0	0	0	0	0	0	0
Lysinibacill	0	0	0	0	0	0	0	0
Selenomon	0	0	0	0	0	0	0	0
Noviherbas	0	0	0	0	0	0	0	0
Merdibacte	0	0	0	0	0	0	0	0
JG36-GS-52	0	0	0	0	0	0	0	0
PLTA13	0	0	0	0	0	0	0	0
MND1	0	0	0	0	0	0	0	0
Shinella	0	0	0	0	0	0	0	0
Coriobacte	0	0	0	0	0	0	0	0
Arenimona	0	0	0	0	0	0	0	0
A21b	0	0	0	0	0	0	0	0
[Eubacteriu	0	0	0	0	0	0	0	0
UCG-007	0	0	0	0	0	0	0	0
[Eubacteriu	0	0	0	0	0	0	0	0
Saccharimo	0	0	0	0	0	0	0	0
Ruminococ	0	0	0	0	0	0	0	0
Chitinopha	0	0	0	0	0	0	0	0
Sphingobiu	0	0	0	0	0	0	0	0
Haliscomer	0	0	0	0	0	0	0	0
NS3a_mari	0	0	0	0	0	0	0	0
Oryzihumu	0	0	0	0	0	0	0	0
Leuconosto	0	0	0	0	0	0	0	0
Bergeyella	0	0	0	0	0	0	0	0
NS11-12_m	0	0	0	0	0	0	0	0

bacteriap25	0	0	0	0	0	0	0	0
Polycyclovo	0	0	0	0	0	0	0	0
Candidatus_	0	0	0	0	0	0	0	0
Microtetras	0	0	0	0	0	0	0	0
Mesorhizob	0	0	0	0	0	0	0	0
Puia	0	0	0	0	0	0	0	0
Pedosphaer	0	0	0	0	0	0	0	0
MB-A2-108	0	0	0	0	0	0	0	0
Chryseobac	0	0	0	0	0	0	0	0
PHOS-HE3	0	0	0	0	0	0	0	0
KF-JG30-B	0	0	0	0	0	0	0	0
Subgroup_2	0	0	0	0	0	0	0	0
IMCC26256	0	0	0	0	0	0	0	0
Nordella	0	0	0	0	0	0	0	0
Tannerella	0	0	0	0	0	0	0	0
Lentimicrol	0	0	0	0	0	0	0	0
Finegoldia	0	0	0	0	0	0	0	0
Rhizobacte	0	0	0	0	0	0	0	2
SWB02	0	0	0	0	0	0	0	0
Dyella	0	0	0	0	0	0	0	0
Devosia	0	0	0	0	0	0	0	0
Solitalea	0	0	0	0	0	0	0	0
Massilia	0	0	0	0	0	2	0	0
Clavibacter	0	0	0	0	0	0	0	0
Roseobacte	0	0	0	0	0	0	0	0

HC28	HC29	HC30	HC31	HC32	HC33	HC34	HC35	HC36
13890	8355	21549	4296	21177	7070	4299	29950	13253
235	19250	160	21685	166	23864	26924	141	14256
18551	6466	939	1751	2771	10593	1195	9121	2709
328	268	7043	558	721	129	101	599	261
105	35	41	42	292	49	28	471	18
815	1593	105	526	3343	100	2159	1837	4116
488	1166	227	695	2180	112	1732	1169	861
643	107	95	273	556	641	864	198	515
690	813	757	261	1783	782	682	730	190
168	20	10	103	76	22	48	249	998
25	18	38	16	23	0	0	12	13
273	190	44	79	47	59	39	845	30
0	17	15	10	49	86	30	94	0
1047	211	2734	86	437	1042	229	304	233
16	27	388	0	0	14	127	14	14
396	107	261	160	384	387	287	359	362
0	25	0	71	13	0	0	0	31
698	355	287	196	0	146	695	1697	1861
461	233	0	234	2522	197	468	527	303
289	1451	0	893	3950	60	830	1396	1031
22	101	13	285	2113	54	825	845	3452
331	1430	0	101	1519	43	291	799	368
688	563	2726	0	395	8	9	298	47
16	0	0	18	0	0	0	0	18
186	444	0	254	687	116	1878	857	133
286	142	135	199	1768	11	0	404	1548
7	14	17	7	17	55	3360	949	63
1027	0	8	675	321	207	716	942	362
7	313	6	0	376	9	215	131	1292
269	334	3	226	212	10	210	508	245
0	67	121	62	234	425	155	45	266
0	116	0	82	114	243	90	93	333
0	432	62	171	1474	432	209	128	234
7	134	12	588	3335	0	0	0	0
0	0	0	0	0	0	7	3	13
60	488	23	1040	1939	176	1537	693	691
255	67	14	350	103	161	531	197	442
7	938	6	294	3072	3	255	791	273
175	21	65	60	328	105	18	204	77
215	9	65	18	29	58	18	69	89
144	417	11	306	665	12	1019	126	614
0	580	0	595	164	11	88	152	118

0	121	0	220	483	3	799	158	95
0	0	0	163	0	449	107	0	365
186	62	302	65	88	103	101	41	232
97	11	23	34	95	78	76	47	103
500	49	11	90	95	9	197	184	271
3	19	4	8	18	0	16	33	17
0	0	5	3686	0	0	3	0	0
0	902	2	88	282	0	330	882	2451
90	20	0	8	53	104	32	89	154
24	185	0	172	107	0	101	63	599
202	45	283	22	66	171	48	74	47
0	0	17	0	0	0	0	0	0
121	18	20	11	102	66	11	11	118
0	6	4	0	6	0	4	0	10
6	60	0	30	309	15	22	354	36
0	2567	0	0	854	95	0	0	459
218	92	68	12	0	118	28	45	16
10	48	0	15	26	426	63	22	69
211	96	0	16	252	25	38	135	7
7	16	14	45	204	58	14	49	43
26	16	44	0	0	0	28	18	4
7	54	0	104	136	0	129	7	257
0	0	0	0	0	0	0	0	0
0	53	0	42	85	0	360	41	27
0	7	0	0	0	0	0	0	0
0	0	0	0	0	0	0	0	7
7	0	19	119	0	0	0	0	0
0	18	0	0	17	0	6	7	49
42	31	0	0	53	0	0	47	19
0	0	0	0	0	0	0	0	0
128	337	0	64	314	91	71	281	136
0	0	0	0	0	0	0	0	0
13	0	0	0	0	0	0	0	0
6	0	0	0	7	13	8	8	5
0	0	0	0	0	0	0	0	0
0	0	0	0	0	0	0	0	0
0	8	12	8	52	7	6	32	5
9	49	6	10	61	0	37	14	39
0	15	0	0	16	15	5	26	15
0	69	57	0	0	0	0	4	18
121	20	10	19	9	27	15	35	12
9	13	0	6	5	0	0	11	0
0	11	23	0	0	30	23	33	20

0	0	0	36	0	0	154	0	0
0	0	0	35	480	0	19	191	0
0	48	0	0	0	0	0	15	0
0	417	0	4	214	4	8	7	3
0	0	0	2599	0	0	0	0	0
0	0	4	0	12	0	0	0	0
0	0	0	81	0	0	52	0	0
42	13	37	0	31	37	46	25	21
0	68	0	36	102	0	49	116	24
0	0	0	0	0	0	6	14	0
0	0	3	0	0	0	0	0	0
8	12	13	0	0	22	10	15	9
0	0	0	0	0	0	0	0	0
0	27	0	24	29	0	16	8	9
0	0	0	0	16	5	0	4	0
0	4	0	76	30	0	8	0	11
26	0	0	13	0	14	6	0	28
0	0	0	0	3	0	0	0	0
0	4	0	0	0	4	0	0	0
0	0	0	0	0	0	0	0	0
0	0	0	0	0	0	0	0	0
0	0	0	204	46	0	0	0	0
0	23	0	45	54	0	38	5	46
0	0	0	0	0	0	8	0	12
0	0	0	0	0	0	0	0	5
19	0	0	18	25	53	0	0	18
15	19	21	23	25	14	15	22	37
6	0	2	0	0	0	0	0	0
0	0	0	0	0	0	3	0	0
0	0	0	0	0	0	5	5	0
0	0	0	7	0	0	0	0	21
0	0	0	0	0	0	0	8	2
0	0	0	0	0	0	0	0	0
0	0	0	0	0	0	0	0	0
0	0	0	744	4	0	0	0	0
38	30	0	0	24	14	10	33	7
0	0	0	0	0	0	0	0	0
0	0	0	0	0	0	7	0	0
0	0	0	0	0	0	0	0	0
0	0	4	0	0	0	31	0	0
0	0	0	0	0	0	0	0	0
0	0	0	0	22	0	0	9	0
0	0	0	0	0	0	0	0	10

0	0	0	0	0	0	0	0	0
0	0	0	0	23	0	0	0	0
0	0	0	15	0	0	0	0	17
0	0	0	0	0	0	0	0	0
0	0	0	0	0	0	0	0	408
0	0	0	0	0	0	0	0	0
0	0	0	0	28	4	0	0	0
0	265	0	0	0	0	0	0	0
0	0	5	0	0	0	19	18	0
0	0	0	0	0	0	0	0	0
0	0	0	90	0	0	0	37	0
11	0	0	0	4	3	0	8	0
0	0	0	0	0	0	0	0	0
0	0	0	0	0	0	0	0	0
0	0	0	0	0	0	0	0	0
0	51	0	18	8	0	11	35	17
0	0	0	0	0	0	0	0	0
0	0	0	0	0	0	0	0	0
0	8	0	0	21	0	0	5	0
0	5	0	0	8	0	0	6	0
7	0	6	0	0	0	3	4	0
0	0	0	0	0	0	0	0	0
0	0	0	0	0	0	0	6	0
0	0	0	0	0	0	0	0	0
0	0	0	0	0	0	0	0	0
6	6	0	0	0	0	0	8	6
0	0	0	0	0	0	0	0	0
0	0	0	0	0	0	0	0	0
0	0	0	9	15	0	6	8	0
0	0	0	0	0	0	0	0	0
0	0	0	0	0	0	0	0	0
0	0	0	0	0	0	0	5	0
0	4	0	0	11	0	0	0	23
0	0	0	0	0	0	0	0	0
0	0	0	0	0	0	0	0	3
0	0	0	0	0	0	0	0	0
0	0	0	0	0	0	0	0	0
0	0	0	0	0	0	0	0	0
0	0	0	0	14	0	0	0	0
0	0	0	0	0	0	0	0	0
0	0	0	0	0	0	0	0	0
0	0	0	0	0	0	0	0	0
0	0	0	0	0	0	0	0	0

0	0	0	0	0	1	14	0	7
0	0	0	0	0	0	0	0	0
0	0	0	0	0	0	0	0	0
0	0	0	0	0	0	0	0	0
0	0	0	0	0	0	0	0	0
0	0	0	0	0	0	0	0	0
0	0	0	0	0	0	0	0	0
0	0	0	0	0	0	0	0	0
0	0	0	0	0	0	0	0	0
0	0	0	0	0	0	0	0	0
0	0	0	0	0	0	0	0	0
0	0	0	0	0	0	0	0	0
0	0	0	0	0	0	0	0	0
0	0	0	0	0	0	0	0	0
0	0	0	0	0	0	66	0	0
0	0	0	0	0	0	0	0	0
0	0	0	0	0	0	0	0	0
0	0	0	0	0	0	0	0	0
0	0	0	13	0	0	0	0	0
0	2	0	0	0	3	3	6	3
0	0	0	0	0	0	0	0	0
0	0	0	0	0	0	0	0	0
0	0	0	0	0	0	0	0	0
0	0	0	0	0	0	0	0	0
0	0	0	0	0	0	0	0	0
0	0	0	0	0	0	0	0	0
0	0	0	0	0	0	0	0	0
0	0	0	0	0	0	0	0	0
0	0	0	0	0	0	0	5	0
3	0	0	0	0	0	0	0	0
0	0	0	0	0	0	0	0	0
5	0	0	0	0	0	0	0	0
0	0	0	0	6	0	0	0	0
0	0	0	0	0	0	0	0	0
0	0	0	0	0	0	0	0	3
0	0	0	0	7	0	0	0	0
0	0	0	0	0	0	0	0	0
0	0	0	0	0	0	0	0	0
0	0	0	0	0	0	0	0	0
0	0	0	0	0	0	0	0	0
0	0	0	0	0	0	0	0	0
0	0	0	0	0	0	0	7	0
0	0	0	0	6	0	0	0	7

0	0	0	0	0	0	0	0	0
0	0	0	0	0	0	0	4	0
0	0	0	4	0	0	0	0	0
0	0	0	0	0	0	0	0	0
0	0	0	0	0	0	0	0	0
0	0	0	0	0	0	0	0	0
0	0	0	0	0	0	0	0	0
0	0	0	0	0	0	0	0	0
0	0	0	0	0	0	0	0	0
0	0	0	0	0	0	0	0	0
0	0	0	0	0	0	0	0	0
0	0	0	0	0	0	0	0	0
0	0	0	0	0	0	0	0	0
0	0	0	0	0	0	0	0	0
0	0	0	0	0	0	0	0	0
0	0	0	0	0	0	0	0	0
0	0	0	0	0	0	0	0	0
0	0	0	0	0	0	0	0	0
0	0	0	0	0	0	0	3	0
0	0	0	0	0	0	0	0	0
0	0	0	0	0	0	0	0	0
0	0	0	0	0	25	0	0	0
0	0	0	0	3	0	0	0	2
0	0	0	0	0	0	0	0	0
0	0	0	0	0	0	0	0	0
0	0	0	0	0	0	0	0	0
0	0	0	0	0	0	3	0	0
0	0	0	0	0	0	0	0	0
0	0	0	0	0	2	0	0	6
0	0	0	0	0	0	0	0	0
0	0	0	0	0	0	0	0	0
0	0	0	0	0	0	0	0	0
0	0	0	0	0	0	0	0	0
0	0	0	0	0	0	0	0	0
0	0	0	0	0	0	0	0	0
0	0	0	0	0	0	0	0	0
0	0	0	0	0	0	0	0	0
0	0	0	0	0	0	0	0	0
0	0	0	0	0	0	0	0	0
0	0	0	0	0	0	0	0	0
0	0	0	0	0	0	0	0	0
0	0	0	0	0	0	0	0	0
0	0	0	0	0	0	0	0	0

0	0	0	0	0	0	0	0	0
0	0	0	0	0	0	0	0	0
0	0	0	0	0	0	0	0	0
0	0	0	0	0	0	0	0	0
0	0	0	0	0	0	0	0	0
0	0	0	0	0	0	0	0	0
0	0	0	0	0	0	0	0	0
0	0	0	0	0	0	0	0	0
0	0	0	0	0	0	0	0	0
0	0	0	0	0	0	0	0	0
0	0	0	0	0	0	0	0	0
0	0	0	0	0	0	0	0	0
0	0	0	0	0	0	0	0	0
0	0	0	0	0	0	0	0	0
0	0	0	0	0	0	0	0	0
0	0	0	0	0	0	0	0	0
0	0	0	0	0	0	0	0	0
0	0	0	0	0	0	0	0	0
0	0	0	0	0	0	0	0	0
0	0	0	0	0	0	0	0	0
0	0	0	0	0	0	0	0	0
0	0	0	0	0	0	0	0	0
4	0	0	0	0	0	0	0	0
0	0	0	0	0	0	0	0	0
0	0	0	0	0	0	0	0	0
0	0	0	6	0	0	0	0	0
0	0	0	0	0	0	0	0	6
0	0	0	0	0	0	0	0	0
0	0	0	0	0	0	0	0	0
0	0	0	0	0	0	0	0	0
0	0	0	0	0	0	3	0	3
0	0	0	0	0	0	0	0	0
0	0	0	0	0	0	0	0	0
0	0	0	0	0	0	0	0	0
0	0	0	0	0	0	0	2	0
0	0	0	0	0	0	0	0	6
0	0	0	0	0	0	0	0	0
0	0	0	0	0	0	0	0	0
0	0	0	0	0	2	0	0	0
0	0	0	0	0	0	0	0	0
0	0	0	0	0	0	0	0	0
0	0	0	0	0	0	0	0	0
0	0	0	0	0	0	0	0	0

0	0	0	0	0	0	0	0	0
0	0	0	0	0	0	0	0	0
0	0	0	0	0	0	0	0	0
0	0	0	0	0	0	0	0	0
0	0	0	0	0	0	0	0	0
0	0	0	0	0	0	0	0	0
0	4	0	0	0	0	0	0	0
0	0	0	0	0	0	0	0	0
0	0	0	0	0	0	0	0	0
0	0	0	0	0	4	0	0	0
0	0	0	0	0	0	0	0	0
0	0	0	0	0	0	0	0	0
0	0	0	0	0	0	0	0	0
0	0	0	0	0	0	0	0	0
0	0	0	0	0	0	0	0	0
0	0	0	0	0	0	0	0	0
0	0	0	0	0	0	0	0	0
0	0	0	0	0	0	0	0	0
0	0	0	0	0	0	0	0	0
0	0	0	0	0	0	0	0	0
0	0	0	0	0	0	0	0	0
0	4	0	0	0	0	0	0	0
0	0	0	0	0	0	0	0	0
0	0	0	0	0	0	0	0	0
0	0	0	0	0	0	0	0	0
0	0	0	0	0	0	0	0	0
0	0	0	0	0	0	0	0	0
0	0	0	0	0	0	0	0	0
0	0	0	0	3	0	0	0	0
0	0	0	0	0	0	0	0	0
0	0	0	0	0	0	0	0	0
0	0	0	0	0	0	0	0	0
0	0	0	0	0	0	0	0	0
0	0	0	0	0	0	0	0	0
0	0	0	0	0	0	0	0	0
0	0	0	0	0	0	0	0	0
0	0	0	0	0	0	0	0	0
0	0	0	0	0	0	3	0	0
0	0	0	0	0	0	0	0	0
0	0	0	0	0	0	0	0	0
0	0	0	0	0	0	0	0	0
0	0	0	0	0	0	0	0	0
0	0	0	0	0	0	0	0	0

0	0	0	0	0	0	0	0	0
0	0	0	0	0	0	0	0	0
0	0	0	0	0	0	0	0	0
0	0	0	0	0	0	0	0	0
0	0	0	0	0	0	0	0	0
0	0	0	0	0	0	0	0	0
0	0	0	0	0	0	0	0	0
0	0	0	0	0	0	0	0	0
0	0	0	0	0	0	0	0	0
0	0	0	0	0	0	0	0	0
0	0	0	0	0	0	0	0	0
0	0	0	0	0	0	0	0	0
0	0	0	0	0	0	0	0	0
0	0	0	0	0	0	0	0	0
0	0	0	0	0	0	0	0	0
0	0	0	0	0	0	0	0	0
0	0	0	0	0	0	0	0	0
0	0	0	0	0	0	0	0	0
0	0	0	0	0	0	0	0	0
0	0	0	0	0	0	0	0	0
0	0	0	0	0	2	0	0	0
0	0	0	0	0	0	0	0	0
0	0	0	0	0	0	0	0	0
0	0	0	0	0	0	0	0	0
0	0	0	0	0	0	0	0	0

HC37	HC38	HC39	HC40	HC41	HC42	HC43	HC44	LC71
19278	26241	8835	9019	23378	11657	29429	9023	713
245	110	32727	7333	55	15885	70	37364	38
9149	1326	2056	1362	4376	1985	3601	719	137
2328	91	53	125	70	66	141	56	147
1567	12	0	0	20	0	31	0	102
39	11764	489	4926	5509	3239	3234	1342	9
2094	745	1292	2237	4241	1164	635	871	43
448	430	218	308	486	418	2707	96	122
0	1101	113	285	597	271	1341	0	0
215	906	369	57	52	136	87	7	11055
2573	19	16	0	0	11	9	0	0
81	35	306	53	58	103	50	370	18
0	73	76	100	23	33	29	0	421
574	312	616	471	993	240	224	115	0
477	7	0	13	0	0	0	10	15
461	2254	372	735	518	254	982	74	14
30	0	213	24	43	0	0	0	10
31	847	2845	911	796	115	2870	23	0
284	177	0	361	435	483	408	0	8
5	198	599	880	2066	1084	168	46	0
35	181	45	2679	165	1987	249	5	10
10	252	798	546	665	168	619	122	0
4558	1115	433	15	108	348	296	0	0
0	0	27	0	0	0	0	13	29012
0	172	899	709	561	172	2198	733	0
7	930	0	2435	1155	2175	1775	0	0
63	73	46	236	92	366	60	50	64
97	3	582	339	273	310	0	946	0
103	6	97	558	76	999	0	0	0
68	696	6	274	311	215	1312	5	0
50	81	320	117	1188	43	331	89	0
5	139	142	269	290	80	131	45	11
351	288	929	55	1114	50	203	57	0
0	0	0	4	0	164	0	0	0
0	4	0	0	0	3	0	0	5643
62	159	33	108	568	664	57	27	15
8	194	61	374	52	1157	280	7	0
8	31	51	144	179	107	43	6	2
0	357	264	90	217	66	258	33	0
0	151	58	61	27	39	117	50	0
0	211	25	222	774	678	605	517	0
9	0	12	157	0	943	7	0	5

0	52	0	100	192	162	0	0	0
0	0	334	0	0	721	3	0	0
0	382	117	149	113	80	128	34	10
0	233	126	154	100	47	348	21	10
106	85	260	217	220	18	227	0	0
0	29	14	0	221	286	175	35	17
0	0	0	0	6	0	10	0	2
2	695	0	1084	527	235	0	0	0
3	50	163	159	35	39	32	9	6
0	298	794	405	220	192	709	183	0
0	27	108	25	74	39	24	14	0
18	0	0	7	0	0	0	0	0
0	78	37	99	77	16	42	18	4
15	7	0	0	0	28	0	0	0
0	159	107	88	72	29	90	3	0
0	0	0	220	0	296	21	0	0
0	62	0	22	845	7	205	5	0
581	29	15	15	14	76	20	0	0
0	131	91	23	377	61	34	16	0
0	40	93	138	42	24	79	14	0
17	0	10	10	23	0	11	13	6
0	203	0	41	20	72	295	0	0
6	0	21	7	18	0	0	0	20
0	113	0	44	11	0	136	0	0
0	0	0	0	0	0	0	0	0
35	0	6	0	0	0	0	0	0
0	10	0	0	0	0	13	0	0
0	22	8	81	11	0	15	11	0
0	38	0	1497	11	13	0	28	0
0	0	0	0	0	0	5	0	5858
0	46	16	138	267	153	0	28	0
0	4	0	0	0	0	0	0	5
0	0	0	0	39	0	12	0	0
0	5	6	10	0	11	0	0	3173
0	0	0	0	0	0	0	0	0
0	0	0	0	0	0	0	0	0
43	4	0	17	58	16	13	0	0
15	9	0	43	196	29	15	6	6
18	15	0	0	24	13	14	0	31
93	0	0	0	0	27	2	0	0
0	0	7	12	37	22	41	13	0
5	0	7	12	0	0	0	0	0
7	17	9	37	23	0	22	0	589

0	0	0	11	0	0	0	0	0
0	0	29	133	13	47	0	0	0
0	0	0	0	0	20	337	0	0
0	0	0	0	0	127	0	0	0
0	0	0	0	0	0	0	0	0
0	0	0	6	0	36	0	0	0
0	0	0	0	0	0	0	0	0
48	38	16	56	22	30	16	17	10
0	0	0	0	0	171	10	0	0
5	5	0	3	6	0	3	0	15
0	0	0	0	0	0	0	0	0
21	25	0	19	8	0	7	8	1605
0	0	8	20	0	0	0	0	0
0	9	8	33	8	22	23	0	0
0	22	42	7	29	10	16	0	0
0	0	2	0	0	0	0	0	0
0	19	9	0	0	0	15	0	0
0	0	0	0	0	0	0	0	0
6	0	0	0	0	0	0	0	417
0	0	0	0	0	0	0	0	0
0	0	0	0	0	0	0	0	139
0	0	0	0	0	0	578	0	0
0	0	0	0	0	0	0	0	0
0	2	0	11	19	0	5	0	0
0	0	0	0	0	0	0	0	0
0	30	0	26	26	0	44	0	0
26	26	28	60	10	5	0	19	0
0	0	0	0	5	0	0	0	0
0	0	0	7	0	0	0	0	31
0	6	4	0	0	4	8	0	0
0	0	0	15	0	11	0	0	0
5	0	0	0	0	4	0	0	0
0	0	0	0	0	15	0	0	0
0	0	0	0	0	0	0	0	0
0	0	0	0	0	0	0	0	0
0	0	0	0	13	12	4	0	0
0	0	0	0	0	0	0	0	0
0	0	0	0	0	0	0	0	0
0	0	0	0	0	0	0	0	0
0	0	0	0	0	0	0	0	0
0	0	0	0	0	0	0	0	0
0	0	20	22	6	10	12	0	0
0	0	0	0	0	0	0	0	0

0	0	0	0	54	0	0	0	0
0	0	7	18	5	7	6	0	0
0	0	28	0	0	9	0	0	0
0	0	0	0	0	0	0	0	0
0	0	0	0	0	0	0	0	0
0	0	0	0	0	0	0	0	576
0	0	0	0	3	0	11	0	0
0	0	0	0	0	0	0	0	0
16	8	0	11	5	17	16	0	7
0	0	0	0	0	0	0	0	0
0	0	0	12	0	0	0	0	0
0	16	0	5	17	0	0	6	0
0	0	12	0	0	0	0	0	0
0	0	0	4	0	0	0	0	0
0	0	0	0	0	0	0	0	0
0	0	0	4	0	10	0	0	0
0	0	0	0	0	0	0	0	0
0	0	0	0	0	0	0	0	0
0	22	0	17	0	0	0	0	0
0	0	0	3	15	0	0	0	0
8	0	0	0	0	0	4	5	0
0	0	0	0	5	0	8	4	0
0	6	0	0	0	4	0	5	0
0	0	0	0	0	0	0	0	0
0	0	0	0	0	0	0	0	0
0	0	0	7	0	2	0	0	0
0	0	0	0	0	0	0	0	0
0	0	0	0	0	0	0	0	0
0	0	0	16	0	0	0	0	0
0	0	0	0	0	0	0	0	0
0	0	0	0	0	0	0	17	0
0	0	0	0	0	0	0	0	0
0	0	0	3	0	6	0	0	0
0	0	0	0	0	0	0	0	0
0	0	0	0	0	0	0	0	0
0	0	0	0	0	0	0	0	0
0	0	0	0	0	0	0	0	0
0	0	0	7	19	0	0	0	0
0	0	0	0	0	0	25	0	0
0	0	0	0	0	0	3	0	0
0	0	0	0	0	0	0	0	0
0	0	0	0	4	0	0	0	0
0	0	0	0	0	0	0	0	0

0	0	0	0	0	0	0	0	0
0	0	0	0	0	0	0	0	0
0	0	0	0	0	0	0	0	0
0	0	0	0	0	0	0	0	0
0	0	0	0	0	0	0	0	0
0	0	0	0	0	0	0	0	0
0	0	0	0	0	0	0	0	129
0	0	0	0	0	0	0	0	0
0	0	0	0	0	0	0	0	119
0	10	0	0	0	0	0	0	0
0	0	0	0	59	0	0	0	0
0	0	0	0	0	0	0	0	0
0	0	0	0	22	0	0	0	0
0	0	0	0	0	0	0	0	0
0	11	0	0	0	8	0	0	0
0	0	0	0	0	0	0	0	0
0	0	0	0	0	0	0	0	0
0	0	0	0	0	0	0	0	0
0	0	0	0	0	0	0	0	0
9	6	5	0	0	0	0	0	0
0	0	0	0	0	0	0	0	0
0	0	0	0	0	0	0	0	0
0	0	0	0	0	0	0	0	0
0	0	0	0	0	0	0	0	0
0	0	0	0	0	0	0	0	0
0	0	0	0	0	0	0	0	7
0	0	0	5	0	11	13	20	0
0	0	0	0	6	0	0	0	0
0	0	0	0	0	0	0	0	0
4	0	0	0	0	0	0	0	0
0	0	0	0	0	0	0	0	0
0	0	0	0	0	0	0	0	0
0	0	0	5	0	0	0	0	0
0	0	0	0	0	0	0	0	0
0	0	0	5	10	6	11	6	0
0	0	0	0	0	0	0	0	0
0	0	0	0	0	0	0	0	0
0	0	0	0	0	0	0	0	0
0	0	0	0	0	0	0	0	0
0	0	0	0	0	0	0	0	0
0	0	0	0	0	0	0	0	0
0	0	0	0	0	0	0	0	0
0	0	0	0	0	0	6	0	0

0	0	0	0	9	10	10	4	0
0	0	0	0	0	0	0	0	0
0	0	0	0	0	0	0	0	0
0	0	0	0	0	0	0	0	0
0	0	0	0	0	0	0	0	0
0	0	0	0	0	0	0	0	0
0	0	0	0	0	0	0	0	0
0	0	0	8	0	0	0	0	0
0	0	0	14	0	0	0	0	0
0	0	0	0	0	0	0	0	0
0	0	0	0	0	8	0	12	0
0	0	0	0	0	0	0	0	32
0	0	0	0	0	0	0	0	0
0	0	4	0	0	0	0	0	0
0	0	0	0	0	0	0	0	0
0	0	0	0	0	0	0	0	0
0	0	0	0	0	5	7	0	0
0	0	0	0	0	0	0	0	4
0	0	0	0	0	0	0	0	0
0	0	0	0	0	0	0	0	0
0	0	0	0	0	0	0	0	0
0	0	0	0	0	0	0	0	0
0	0	0	4	5	0	0	0	0
0	0	0	0	0	0	0	0	0
0	0	0	0	0	0	0	0	0
0	0	0	0	0	0	0	0	0
0	0	0	0	0	5	7	0	0
0	0	0	0	0	0	0	0	0
0	0	0	0	0	0	0	0	0
0	0	0	0	9	0	0	0	0
0	0	3	0	0	0	0	0	7
0	0	0	0	0	0	0	0	0
0	0	0	0	0	0	0	0	0
0	0	0	0	0	0	0	0	0
0	0	0	0	0	0	0	0	0
0	0	0	0	0	0	0	0	0
0	0	0	0	0	0	0	0	0
0	0	0	0	0	0	0	0	0
0	0	0	0	0	0	0	0	0
0	0	0	0	0	0	0	0	0
0	0	0	0	0	0	0	0	0
0	0	0	0	0	0	0	0	0
0	0	0	0	0	0	0	0	0

0	0	0	0	0	0	0	0	0
0	0	0	0	0	0	0	0	0
0	0	0	0	0	0	0	0	0
0	0	0	0	0	0	0	0	11
0	0	0	0	0	0	0	0	0
0	0	0	0	0	0	0	0	0
0	0	0	0	0	0	0	0	0
0	0	0	0	0	0	0	0	0
0	0	0	0	0	0	0	0	0
0	0	0	0	0	0	0	0	0
0	0	0	0	0	0	0	0	0
0	0	0	0	0	0	0	0	0
0	0	0	0	0	0	0	0	0
0	0	0	0	0	0	0	0	0
0	0	0	0	0	0	0	0	0
0	0	0	0	0	0	0	0	0
0	0	0	0	0	0	0	0	0
0	0	0	0	0	0	0	0	0
0	0	0	0	0	0	0	0	0
0	0	0	0	0	0	0	0	0
0	0	0	0	0	0	0	0	0
0	0	0	0	0	0	0	0	0
0	0	0	0	0	0	0	0	0
0	0	0	0	0	0	0	0	0
0	0	0	0	0	0	0	0	2
0	0	0	0	0	0	0	0	0
0	0	0	0	0	0	0	0	0
0	0	0	6	0	0	0	0	0
0	0	0	0	0	0	0	0	0
0	0	0	6	0	0	0	0	0
0	0	0	0	0	0	0	0	0
0	0	0	0	0	0	0	0	0
0	0	0	0	3	3	0	0	0
0	0	0	0	0	0	0	0	0
0	0	0	0	0	0	0	0	0
0	0	0	0	0	0	0	0	0
0	0	0	0	0	0	0	0	0
0	0	0	0	0	0	0	0	0
0	0	0	0	0	0	0	0	0
0	0	0	0	0	0	0	0	0
0	0	5	0	0	0	0	0	0
0	0	0	0	0	0	0	0	0
0	0	0	0	0	0	0	0	0

0	0	0	0	0	0	0	0	5
0	0	0	0	0	0	0	0	0
0	0	0	0	0	0	0	0	0
0	0	0	0	5	0	0	0	0
0	0	0	0	0	0	0	0	0
0	0	0	0	0	0	0	0	4
0	0	0	0	0	0	0	0	0
0	0	0	0	0	0	0	0	0
0	4	0	0	0	0	0	0	0
0	0	0	0	0	0	0	0	0
4	0	0	0	0	0	0	0	0
0	0	0	0	0	0	0	0	0
0	0	0	0	0	0	0	0	0
0	0	0	0	0	0	0	0	0
0	0	0	0	0	0	0	0	0
0	0	0	0	0	0	0	0	0
0	0	0	0	0	0	0	0	0
0	0	0	0	0	0	0	0	0
0	0	0	0	0	0	0	0	0
0	0	0	0	0	0	0	0	0
0	0	0	0	0	0	0	0	0
0	0	0	0	0	0	0	0	0
0	0	0	0	0	0	0	0	0
3	0	0	0	0	0	0	0	0
0	0	0	0	0	0	0	0	0
0	0	0	0	0	0	0	0	0
0	0	0	0	0	0	0	0	0
0	0	0	0	0	0	0	0	0
0	0	0	0	0	0	0	0	0
0	0	0	0	0	0	0	0	0
0	0	0	0	0	0	0	0	0
0	0	0	0	0	0	0	0	0
0	0	0	0	0	0	0	0	0
0	0	0	0	0	0	0	0	3
0	0	0	0	0	0	0	0	0
0	0	0	0	0	0	0	0	0
0	0	0	0	0	0	0	0	0
0	0	0	0	0	0	0	0	0
0	0	0	0	0	0	0	0	0
0	0	0	0	0	0	0	0	0
0	0	0	0	0	0	0	0	0
0	0	0	0	0	0	0	0	0
0	0	0	0	0	0	0	0	0

0	0	0	0	0	0	0	0	0
0	0	0	0	0	0	0	0	0
0	0	0	0	0	0	0	0	0
0	0	0	0	0	0	0	0	0
0	0	0	0	0	0	0	0	0
0	0	0	0	0	0	0	0	0
0	0	0	2	0	0	0	0	0
0	0	0	0	0	0	0	0	0
0	0	0	0	0	0	0	0	0
0	0	0	2	0	0	0	0	0
0	0	0	0	0	0	0	0	0
0	0	0	0	0	0	0	0	0
0	0	0	0	0	0	2	0	0
0	2	0	0	0	0	0	0	0
0	0	0	0	0	0	0	0	0
0	0	0	0	0	0	0	0	0
0	0	0	0	0	0	0	0	0
0	0	0	0	0	0	0	0	0
0	0	0	0	0	0	0	0	0
0	0	0	0	0	0	0	0	0
0	0	0	0	0	0	0	0	0
0	0	0	0	0	0	0	0	0
0	0	0	0	0	0	0	0	0
0	0	0	0	0	0	0	0	0
0	0	0	0	0	0	0	0	0

LC72	LC73	LC74	LC75	LC76	LC77	LC78	LC79	LC80
44118	24366	11056	29092	33569	44195	2549	26574	41359
169	47	78	70	70	2395	21968	10472	153
1652	6593	7420	1280	23	489	3096	5942	2163
6016	1272	971	213	50	556	103	55	6790
22	6564	4261	0	2697	505	54	90	63
37	61	33	75	17	418	458	361	6
1888	787	608	259	34	2008	152	257	15
1536	558	518	85	1719	84	193	1279	424
5	4630	3392	17	15	15	3123	2444	55
12	760	1829	37	11	484	25	89	1899
0	271	256	436	118	11	9	23	268
24	62	75	17	17	31	158	498	25
1522	243	752	0	48	1138	15	340	121
0	2580	2536	935	21	120	142	605	269
3726	77	98	805	3969	260	10	179	328
9	380	1259	889	10	974	139	284	1139
224	95	38	45	22127	230	0	0	0
0	1170	1655	0	0	0	39	505	492
5	42	67	0	0	0	406	1712	396
0	0	3	5	0	141	480	484	13
7	419	1245	0	0	54	141	75	0
13	414	425	20	10	31	263	247	118
3	0	0	1320	7	13	261	22	0
94	125	197	24	88	0	5	17	9
11	45	30	0	0	178	955	805	200
0	0	0	0	0	114	213	20	0
82	4	0	4	27	47	1475	1195	114
3	2	7	0	6	2325	2	1123	3
8	711	712	9	4	6	3169	5	914
0	0	0	0	0	6	128	2063	4
0	635	319	12	0	1504	98	293	179
5	400	527	0	0	624	50	3	5
0	0	0	0	13	0	1947	476	221
0	0	0	6	0	0	701	1338	0
13	974	5000	0	3	31	4	13	315
0	177	210	0	6	72	164	95	126
0	297	237	0	0	364	1472	808	6
0	0	0	0	0	8	78	48	0
0	186	259	15	0	141	24	32	849
0	310	608	131	0	81	8	51	663
16	16	0	161	0	99	22	38	0
0	3	0	0	2	0	903	30	0

0	0	0	0	0	0	130	0	0
12	0	0	0	0	0	68	275	0
0	313	407	138	0	167	132	185	206
0	96	1031	22	0	0	17	114	114
0	0	0	0	0	0	8	44	1221
23	14	5	0	6	32	0	26	249
0	0	0	0	0	0	2262	0	3
0	0	0	0	0	0	55	0	0
0	213	348	60	0	68	7	117	578
0	0	0	0	0	25	19	10	0
0	885	351	58	0	0	106	295	154
31	0	0	5	301	18	0	3	51
0	265	542	341	0	0	48	119	146
889	99	57	0	5	0	43	185	10
0	0	0	18	0	0	78	104	131
0	0	0	0	0	0	0	0	0
10	0	0	0	0	120	40	198	0
0	0	0	0	0	145	47	92	27
13	262	127	29	0	76	15	101	45
0	41	101	17	0	0	23	37	0
8	144	53	5	384	60	0	22	611
0	0	0	0	0	35	38	57	0
373	17	7	87	15	63	0	0	177
0	0	0	0	0	0	86	118	0
0	0	0	0	0	0	0	0	0
237	3	0	0	220	38	0	0	20
0	617	1003	0	4	0	0	20	192
13	0	0	0	0	171	18	0	0
0	175	811	0	0	0	0	0	0
11	0	8	0	0	0	0	0	0
0	0	0	0	0	17	39	195	0
0	0	0	0	0	0	0	0	0
13	0	0	18	2	0	0	6	53
53	0	0	0	3	3	4	0	8
0	0	0	0	0	0	0	0	0
0	0	0	0	0	0	0	0	0
18	10	15	0	0	21	34	34	24
0	36	42	0	0	74	9	0	0
19	18	0	0	0	28	0	6	23
0	0	0	0	0	0	6	0	10
0	0	0	0	0	17	14	16	0
8	0	0	0	0	0	0	5	0
341	9	38	0	0	0	0	10	31

0	0	0	0	0	0	0	0	0
10	0	0	0	0	16	0	10	8
0	0	0	0	0	0	0	185	6
0	0	0	0	0	0	0	335	0
0	0	0	0	0	10	0	0	0
0	0	0	13	0	0	67	0	17
0	0	0	0	231	0	101	0	0
8	15	9	13	20	19	26	12	14
0	0	0	0	0	3	74	0	0
9	3	0	0	2	20	0	3	22
0	0	0	0	0	0	0	0	0
0	0	6	5	4	5	5	0	0
0	0	0	0	0	0	0	0	0
0	0	0	0	0	45	5	2	0
0	9	171	0	0	0	5	32	0
0	0	0	0	0	0	4	0	0
0	0	0	3	0	0	7	0	0
0	0	0	0	0	0	0	0	0
207	0	0	0	0	0	0	11	0
0	0	0	0	0	0	0	0	0
0	312	228	0	0	0	0	4	0
0	0	0	0	0	0	19	0	0
0	0	0	0	0	0	4	0	0
0	7	0	0	0	0	0	6	0
3	59	201	0	0	0	0	0	0
0	0	0	0	0	85	0	0	0
11	4	0	0	14	0	12	0	0
0	0	2	0	0	9	0	0	0
28	14	3	0	0	0	0	0	0
0	0	0	0	0	0	0	0	8
0	0	0	0	0	0	10	0	0
0	43	11	0	0	0	0	0	5
0	0	0	0	0	0	30	0	0
0	0	0	0	0	0	0	0	0
0	0	0	0	0	0	128	0	0
0	0	0	0	0	30	19	58	0
0	0	0	0	0	0	0	0	0
0	0	17	0	0	0	0	0	0
0	0	0	0	0	0	0	0	0
0	0	0	0	0	0	0	0	100
0	0	0	0	0	0	0	0	0
0	0	8	0	0	16	0	0	72
0	0	0	0	0	0	0	0	0

0	0	0	0	0	0	0	0	0
0	0	17	0	0	12	0	8	0
0	0	0	0	0	0	0	0	0
51	0	2	0	0	0	0	0	0
0	0	0	0	0	0	0	0	0
0	0	0	0	0	0	0	0	0
0	0	0	0	0	0	0	0	0
0	0	0	0	0	0	0	0	0
12	0	8	0	0	0	0	0	0
0	0	0	0	0	0	0	0	0
0	0	0	0	0	0	18	0	0
12	0	0	8	0	4	0	10	0
0	38	54	0	0	0	28	0	0
7	0	0	0	0	0	0	0	7
0	0	0	0	0	4	0	0	0
0	0	0	0	0	0	10	12	0
18	0	0	0	0	0	0	0	0
0	0	0	0	0	0	0	0	0
0	0	0	0	0	11	6	0	0
0	0	0	0	0	5	0	0	0
4	0	0	0	0	0	0	4	11
0	0	0	0	0	0	0	0	0
0	0	0	0	0	0	0	0	0
0	0	0	0	0	6	6	6	0
0	0	0	0	0	0	0	0	0
110	0	3	0	3	0	0	0	0
0	0	0	0	0	0	0	0	0
0	0	0	0	0	14	0	0	0
0	0	0	0	0	0	0	20	0
0	0	0	0	0	0	0	0	0
0	0	0	0	0	3	0	0	0
14	0	0	0	0	0	0	0	0
0	0	0	0	0	3	0	0	0
0	0	0	0	0	0	0	0	0
0	0	0	0	0	0	0	0	0
0	0	0	0	0	0	0	0	0
0	0	0	0	0	0	0	0	0
0	0	0	4	0	0	0	0	0
0	0	0	0	0	0	0	0	0
0	0	0	0	0	0	0	0	0
0	0	0	0	0	0	0	0	0
0	0	0	0	0	6	0	0	0
0	0	0	0	0	0	0	0	0

0	0	0	6	0	0	11	0	0
0	0	0	0	0	0	0	0	0
0	0	0	0	0	0	0	0	0
0	0	0	0	0	0	0	0	0
0	0	2	0	0	0	0	0	0
0	0	0	0	0	0	0	0	0
0	0	0	0	0	0	0	0	0
7	0	0	0	0	0	0	0	0
0	0	0	0	0	0	0	0	0
0	0	0	0	0	0	0	0	0
0	0	0	0	0	0	0	0	0
0	0	0	0	0	0	0	0	0
0	0	0	0	0	0	0	0	0
0	0	0	0	0	0	0	0	0
0	0	0	0	0	0	0	0	0
0	0	0	0	0	2	0	0	0
0	0	0	0	0	0	0	0	0
0	32	6	0	0	0	0	0	0
0	0	0	0	0	0	0	0	0
0	0	0	0	0	0	0	0	6
93	0	0	0	0	0	0	0	0
0	0	0	0	0	0	0	0	0
0	0	0	0	0	0	0	0	0
0	0	0	0	0	0	0	0	0
0	0	20	0	0	0	0	0	0
0	0	0	0	0	0	0	0	0
0	0	0	0	0	0	0	0	0
2	0	0	0	0	0	0	0	0
0	0	0	0	0	0	0	0	0
0	0	0	0	0	0	0	0	0
0	0	0	0	0	0	0	0	0
0	0	0	0	0	0	0	0	13
0	0	0	0	0	0	0	0	0
0	0	0	0	0	0	0	0	0
0	0	0	0	0	0	0	0	0
0	0	0	0	0	0	0	0	0
0	0	0	0	0	0	0	0	0
0	0	0	0	0	0	0	0	0
0	0	0	0	0	0	0	0	0
0	0	0	0	0	0	0	0	0
0	0	0	0	0	0	0	0	0
0	0	0	0	0	0	0	0	0
0	0	0	0	0	0	0	0	0

0	0	0	0	0	0	0	0	0
0	0	0	0	0	0	0	0	0
0	0	0	0	0	0	0	0	0
0	0	0	0	0	0	0	0	0
11	0	0	0	0	0	0	0	0
0	0	0	0	6	0	0	0	0
0	0	0	0	0	0	0	0	0
0	0	0	0	0	8	0	0	0
0	0	0	0	0	0	0	0	0
0	0	0	0	0	0	0	0	0
0	0	0	0	0	0	0	0	0
0	0	0	0	0	0	0	0	0
0	8	0	0	0	0	0	0	0
0	0	0	0	0	0	0	2	0
0	0	0	0	0	0	0	0	0
0	0	0	0	0	0	0	0	0
0	0	0	0	0	0	0	0	0
0	0	0	0	0	0	0	0	0
0	0	0	0	0	0	0	0	0
0	0	0	0	0	0	0	0	0
0	0	0	0	0	0	0	0	0
0	0	0	0	0	0	0	0	0
0	0	0	0	0	0	0	0	0
4	0	0	0	0	0	0	0	0
0	0	0	0	0	0	0	0	0
0	0	0	0	0	0	0	0	0
0	0	0	0	0	0	0	0	0
0	0	0	0	0	0	0	0	0
0	0	0	0	0	0	0	0	0
0	0	0	0	0	0	0	0	0
0	0	0	0	0	0	0	0	0
0	0	0	0	0	0	0	0	0
0	0	0	0	0	0	0	0	0
0	0	0	0	0	0	0	0	6
0	0	0	0	0	0	0	0	0
0	0	0	0	0	0	0	0	0
0	0	0	0	0	0	0	0	0
0	0	0	0	0	0	0	0	0
0	0	0	0	0	0	0	0	0
0	0	0	0	0	0	0	0	0
0	0	0	0	0	0	0	0	0
0	0	0	0	0	0	0	0	0
0	0	0	0	0	0	0	0	0

0	0	0	0	0	0	0	0	0
0	0	0	0	0	0	0	0	0
0	0	0	0	0	0	0	0	0
0	0	0	0	0	0	0	0	0
0	0	0	0	0	0	0	0	0
0	0	0	0	0	0	0	0	0
0	0	0	0	0	0	0	0	0
0	0	0	0	0	0	0	0	0
0	0	0	3	0	0	0	0	0
0	0	0	0	0	5	0	0	0
0	0	0	0	0	0	0	0	9
0	0	0	0	0	0	0	0	9
0	0	0	0	0	0	0	0	0
0	0	0	0	0	0	0	0	0
0	0	0	0	0	0	0	0	0
0	0	0	0	0	0	0	0	0
0	0	0	0	0	0	0	0	0
8	0	0	0	0	0	0	0	0
0	0	0	0	0	0	0	0	0
0	0	0	0	0	0	0	0	0
0	0	0	0	0	0	0	0	0
0	0	0	0	0	0	0	0	0
0	0	0	0	0	0	0	0	0
0	0	0	0	0	0	0	0	0
0	0	0	0	0	0	0	0	0
0	0	0	0	0	0	0	0	0
0	0	0	0	0	0	0	0	0
0	0	0	0	0	0	0	0	0
0	0	0	0	0	0	0	0	0
0	0	0	0	0	0	0	0	0
0	0	0	0	0	0	0	0	0
0	0	0	0	0	0	0	0	0
0	0	0	0	0	0	0	0	0
0	0	0	0	0	0	0	0	0
0	0	0	0	0	0	0	0	0
0	0	0	0	0	0	0	0	0
0	0	0	0	0	0	0	0	0
0	0	0	0	0	0	0	0	0
0	0	0	0	0	0	0	0	0
0	0	0	0	0	0	0	0	0
0	0	0	0	0	0	0	0	0
0	0	0	0	0	0	0	0	0
0	0	0	0	0	0	0	0	0

0	0	0	0	0	0	0	0	0
0	0	0	0	0	0	0	0	0
0	0	0	0	0	0	0	0	0
0	0	0	0	0	0	0	0	0
0	0	0	0	0	0	0	0	0
0	0	0	0	0	0	0	0	0
0	0	0	0	0	0	0	0	0
0	0	0	0	0	0	0	0	0
0	0	0	0	0	0	0	0	0
0	0	0	0	0	0	0	0	0
0	0	0	0	0	0	0	0	0
0	0	0	0	0	0	0	0	0
0	0	0	0	0	0	0	0	0
0	0	0	0	0	0	0	0	0
0	0	0	0	0	0	0	0	0
0	0	0	0	0	0	0	0	0
0	0	0	0	0	0	0	4	0
0	0	0	0	0	0	0	0	0
0	0	0	0	0	0	0	0	0
0	0	0	0	4	0	0	0	0
0	0	0	0	0	0	0	0	0
0	0	0	0	0	0	0	0	0
0	0	0	0	0	0	0	0	0
0	0	0	0	0	0	0	0	0
0	0	0	0	0	0	0	0	0
0	0	0	0	0	0	0	0	0
0	0	0	0	0	0	0	0	0
0	0	0	0	0	0	0	0	0
0	0	0	0	0	0	0	0	0
0	0	0	0	0	0	0	3	0
0	0	0	0	0	0	0	0	0
0	0	0	0	0	0	0	0	0
0	0	0	0	0	0	0	0	0
0	0	0	0	0	0	0	0	0
0	0	0	0	0	0	0	0	0
0	0	0	0	0	0	0	0	0
0	0	0	0	0	0	0	0	0
0	0	0	0	0	0	0	0	0
0	0	0	0	0	0	0	0	0
0	0	0	0	3	0	0	0	0
0	0	0	0	0	0	0	0	0
0	0	0	0	0	0	0	0	0
0	0	0	0	0	0	0	0	0

0	0	0	0	0	0	0	0	0
0	0	0	0	0	0	0	0	0
0	0	0	0	0	0	0	0	0
0	0	0	0	0	0	2	0	0
0	0	0	0	0	0	0	0	0
0	0	0	0	0	0	0	0	0
0	0	0	0	0	0	0	0	0
0	0	0	0	0	0	0	0	0
0	0	0	0	0	0	0	0	0
0	0	0	0	0	0	0	0	0
0	0	0	0	0	2	0	0	0
0	0	0	0	0	0	0	0	0
0	0	0	0	0	0	0	0	0
0	0	0	0	0	0	0	0	0
0	0	0	0	0	0	0	0	0
0	0	0	0	0	0	0	0	0
0	0	0	0	0	0	0	0	0
0	0	0	0	0	0	0	0	0
0	0	0	0	0	0	0	0	0
0	0	0	0	0	0	0	0	0
0	0	0	0	0	0	0	0	0
0	0	0	0	0	0	0	0	0
0	0	0	0	0	0	0	0	0
0	0	0	0	0	0	0	0	0
0	0	0	0	0	2	0	0	0

LC81	LC82	LC83	LC84	LC85	LC86	LC87	LC88	LC89
1223	67	107	1150	13151	19042	10006	15557	16734
30712	31895	118	41454	153	43	22026	1830	104
696	1435	6758	2531	21122	2124	153	641	2574
48	10645	1968	714	3246	9903	267	2020	13134
37	349	17826	315	0	25	22	90	0
304	7	0	83	9	18	1036	9	9
191	7	0	255	1463	78	1299	133	4763
220	3080	3066	113	146	3	62	179	91
492	2	0	199	8	1005	1204	19	19
105	0	6	1025	70	626	6	365	100
0	269	0	0	880	381	60	14497	1496
23	60	14	19	727	9019	34	4854	50
17	925	71	26	4906	2815	10	2310	8456
67	0	1696	96	703	6	320	172	65
0	240	0	7	1553	7644	23	1050	2371
79	243	0	78	56	111	69	0	0
0	245	5996	0	0	0	0	137	166
20	0	0	54	0	12	36	9	16
43	9	0	206	128	169	0	0	12
246	2	0	5	0	0	14	0	0
18	0	6	374	7	7	3	4	0
11	0	0	48	7	0	0	24	10
295	6	2	13	0	0	3	7	3
16	20	14492	28	13	0	6	0	2536
177	984	0	23	0	0	0	30	0
361	0	0	0	0	0	0	0	0
747	0	0	0	0	0	0	6	2
0	0	17	552	722	5	1416	161	0
6426	301	9	3	514	32	15	401	4
153	343	0	30	0	0	59	0	0
516	0	10	37	0	0	567	708	1372
52	0	0	241	14	0	14	51	23
344	0	0	0	0	0	0	0	0
741	0	6	8	0	0	0	0	0
70	0	10	644	42	0	0	829	454
89	0	0	32	3	13	44	161	6
804	0	0	29	114	6	0	0	0
8	0	0	4	0	0	2	0	0
10	324	0	26	26	0	0	0	0
18	9	0	0	55	0	0	6	0
64	0	0	10	0	0	129	0	0
7	0	0	0	0	0	0	9	0

38	0	0	0	0	0	4	0	0
18	0	0	0	0	0	0	0	0
49	0	0	191	0	0	15	0	7
26	0	0	49	8	3	21	0	0
22	0	0	0	0	10	7	0	0
0	0	0	0	8	0	0	10	23
4331	7	4	3	0	0	0	128	0
5	0	0	0	0	0	0	0	0
17	0	0	4	61	0	13	0	0
15	0	0	10	0	0	6	0	0
50	974	0	132	217	32	104	0	0
0	0	0	0	0	56	0	41	40
385	0	154	14	4	0	4	0	5
39	393	506	0	1425	208	0	0	3
37	0	0	0	0	0	0	0	0
0	0	0	0	0	4	0	0	0
10	21	0	11	0	0	32	0	0
29	0	0	0	0	423	0	0	0
8	0	0	3	0	0	6	0	89
0	2184	0	0	67	0	3	0	0
0	0	11	0	0	0	0	75	556
0	0	0	42	0	0	109	0	0
0	45	3797	6	0	0	24	0	25
0	0	0	0	0	0	0	0	0
0	0	0	0	0	0	0	0	0
0	0	6	0	0	32	0	57	259
0	155	0	0	231	0	0	468	0
4	0	0	0	0	0	0	26	1421
0	0	0	0	0	0	0	0	0
0	0	0	0	0	0	0	0	0
97	0	0	26	0	0	111	4	0
0	0	0	0	0	0	0	0	0
0	0	3	0	0	0	0	0	50
0	0	0	0	36	19	4	0	10
0	398	115	0	556	217	0	0	0
0	0	0	0	0	0	0	0	62
14	0	9	0	0	0	0	0	101
62	0	0	0	0	0	0	0	0
0	2	0	0	0	0	0	0	0
0	0	0	0	0	2545	0	0	0
9	0	0	7	0	0	15	0	0
0	0	0	0	518	0	0	2269	6
0	13	10	14	37	40	9	66	10

0	0	0	43	0	0	0	0	0
0	0	0	3	0	0	0	11	0
0	0	0	0	0	0	0	0	0
0	0	0	0	0	0	0	0	0
0	0	0	0	0	0	0	0	0
30	88	0	0	0	0	0	0	0
29	0	0	58	29	0	5	0	0
7	9	12	17	63	79	15	187	28
108	0	0	0	0	0	0	0	0
0	0	0	0	2	0	0	4	0
0	0	0	0	0	0	10	0	0
0	7	4	4	5	10	7	0	13
0	0	0	0	0	0	0	0	0
6	0	0	0	0	0	13	7	0
0	0	0	0	0	0	0	0	0
7	0	0	0	0	0	0	0	0
147	0	0	7	0	0	0	17	0
0	0	0	0	0	0	0	0	1734
0	0	0	0	51	95	0	117	0
0	0	0	0	231	0	0	1008	0
0	17	0	0	129	42	0	104	0
209	0	0	52	0	0	0	0	0
0	0	0	0	0	0	0	0	0
0	0	0	0	0	0	0	0	0
0	0	0	0	0	0	0	0	1182
14	0	0	0	0	0	0	0	0
8	0	0	10	8	16	17	17	11
0	0	0	0	0	0	0	0	0
0	0	0	0	52	0	0	1167	0
0	0	0	0	0	0	0	0	0
0	0	0	9	0	0	7	28	0
0	34	0	0	487	14	0	140	0
0	0	0	0	0	0	0	0	0
0	0	0	0	0	0	0	0	0
98	0	0	0	0	0	0	0	0
16	0	0	0	0	0	0	0	0
0	0	377	0	0	0	0	0	10
0	4	0	0	10	0	0	50	0
0	0	0	0	0	0	0	0	0
0	0	0	0	0	0	0	0	238
0	0	0	0	0	0	0	0	0
0	0	0	0	0	0	0	0	0
0	0	0	0	0	0	0	0	0

0	0	0	0	0	0	0	0	0
0	0	0	0	0	0	10	0	0
0	0	0	0	0	0	0	0	0
0	0	0	0	14	56	0	68	0
0	0	0	0	6	0	0	18	0
0	0	0	0	0	0	0	0	0
0	0	0	0	0	0	0	0	0
0	0	0	0	0	0	0	0	0
0	0	0	0	11	16	0	60	0
0	0	0	0	0	0	0	0	0
0	0	0	0	0	9	0	0	0
0	0	0	0	0	0	0	0	0
0	0	0	0	0	13	0	22	0
0	7	0	0	16	50	0	207	0
0	0	0	0	0	0	0	0	0
8	0	0	0	0	0	0	0	0
0	0	0	0	7	0	0	132	0
0	0	0	0	0	0	0	0	282
6	0	0	0	0	0	0	0	0
0	0	0	0	0	0	0	0	20
0	0	0	0	12	3	0	19	0
0	0	0	234	0	0	0	0	0
0	0	0	0	0	0	0	0	0
0	4	0	0	0	0	0	0	0
0	0	293	0	0	0	0	0	0
0	0	0	0	0	3	0	0	7
0	0	0	0	0	0	0	0	0
0	0	0	0	0	0	0	0	0
0	0	0	0	0	0	3	0	0
0	0	0	0	0	0	0	0	0
0	0	0	0	0	0	0	0	41
0	0	0	0	9	0	0	72	0
0	0	0	0	0	0	0	0	0
0	0	0	0	0	0	0	48	0
0	0	0	0	0	0	0	0	0
0	0	0	0	0	0	0	0	0
0	0	0	0	0	0	0	0	0
0	0	0	0	5	0	0	0	0
42	0	0	0	0	0	0	0	0
0	0	0	0	0	0	0	0	0
0	0	0	0	0	0	195	0	0
0	0	0	0	0	0	0	0	0
0	0	0	0	0	0	0	0	0

0	0	0	0	4	0	2	7	0
0	0	0	0	0	0	0	0	0
0	0	0	0	3	0	0	21	0
0	0	0	0	0	0	0	0	0
0	0	0	0	0	0	0	36	0
0	0	0	0	0	0	0	65	0
0	0	0	0	0	0	0	0	0
0	0	0	0	28	8	0	0	0
0	0	0	0	0	0	0	0	0
0	0	0	0	0	0	0	0	0
0	0	0	0	0	0	0	0	0
0	0	0	0	0	0	0	0	0
0	0	0	0	0	0	0	0	0
0	0	0	0	0	0	0	0	0
0	0	0	0	0	0	0	0	0
0	0	0	0	0	0	0	0	0
0	0	0	0	0	0	0	0	0
0	0	0	0	5	3	0	0	8
0	0	0	0	0	0	0	0	0
0	0	0	0	0	0	0	0	0
0	0	0	0	0	0	0	0	0
0	0	0	0	0	0	0	0	0
0	0	0	0	0	0	0	0	0
0	0	0	0	0	0	0	0	0
0	0	0	0	7	10	0	0	0
0	0	0	0	0	0	0	48	0
0	0	0	0	0	0	0	0	0
0	0	0	0	0	0	0	0	0
0	0	0	0	0	0	0	0	0
0	0	0	0	0	3	0	6	0
0	0	0	0	11	0	0	0	0
0	0	0	0	0	0	0	0	0
0	0	0	0	0	0	0	0	12
0	56	0	0	0	0	0	0	0
0	0	0	0	0	0	0	0	0
0	0	0	0	0	0	0	0	0
0	0	0	0	0	0	0	0	0
0	0	0	0	0	0	0	0	0
0	0	0	0	0	0	0	0	0
0	0	0	0	0	0	0	0	0
0	0	0	0	0	0	0	0	0
0	0	0	0	3	0	0	0	0
0	0	0	0	0	0	0	0	0

0	0	0	0	0	0	0	0	0
0	0	0	0	0	0	0	0	0
0	0	0	0	0	0	0	0	0
0	0	0	0	0	0	0	0	0
0	0	0	0	0	0	0	0	0
0	0	0	0	0	0	0	0	0
0	0	0	0	0	0	0	0	0
0	0	0	0	0	0	0	0	0
0	0	0	0	0	0	0	0	0
0	0	0	0	0	0	0	0	0
0	0	0	0	0	0	0	0	0
0	0	0	0	0	0	0	0	0
0	0	0	0	10	0	0	0	0
0	0	0	0	0	0	0	0	0
0	0	0	0	0	0	0	0	0
0	0	0	0	0	0	0	0	0
0	0	0	0	0	0	0	0	0
0	0	0	0	0	0	0	0	0
0	0	0	5	0	0	0	0	0
0	0	0	0	0	0	0	0	0
0	0	0	0	0	0	0	0	0
0	0	0	0	0	0	0	0	0
0	0	0	0	0	0	0	0	0
0	0	0	0	0	0	0	5	0
0	0	0	0	0	0	0	0	0
0	0	0	0	0	0	0	0	0
0	0	0	0	0	0	0	0	0
0	0	0	0	0	0	0	0	0
0	0	0	0	0	0	0	0	0
0	0	0	0	0	0	0	0	0
0	0	0	0	0	0	0	0	0
0	0	0	0	0	0	0	0	0
0	0	0	0	0	0	0	0	0
0	0	0	0	0	0	0	0	0
0	0	0	0	0	0	0	0	0
0	0	0	0	0	0	0	0	0
0	0	0	0	0	0	0	0	0
0	0	0	0	0	0	0	0	0
0	0	0	0	0	0	0	0	0
0	0	0	0	0	0	0	0	0
0	0	0	0	0	0	0	12	0
0	0	0	0	0	0	0	0	0
0	0	0	0	0	0	0	11	0

0	0	0	0	0	0	0	0	0
0	0	0	0	0	0	0	5	0
0	0	0	0	0	0	0	0	0
0	0	0	0	0	0	0	0	0
0	0	0	0	10	0	0	0	0
0	0	0	0	0	0	0	0	0
0	0	0	0	0	0	0	0	0
0	0	0	0	0	0	0	0	0
0	0	0	0	4	0	0	0	0
0	0	0	0	0	0	4	0	0
0	0	0	0	0	0	0	0	0
0	0	0	0	0	0	0	0	0
0	0	0	0	0	0	0	0	0
0	0	0	0	0	0	0	0	0
0	0	0	0	0	0	0	0	0
0	0	0	0	0	0	0	0	0
0	0	0	0	0	0	4	0	0
0	0	0	0	0	0	0	0	0
0	0	0	0	0	0	0	0	0
0	0	0	0	0	0	0	0	0
0	0	0	0	0	0	0	8	0
0	0	0	0	0	0	0	0	0
0	0	0	0	0	0	0	0	0
0	0	0	0	0	0	0	0	0
0	0	0	0	0	0	0	0	0
0	0	0	0	0	0	0	0	0
0	0	0	0	0	0	0	0	0
0	0	0	0	0	0	0	0	0
0	0	0	0	0	0	0	0	0
0	0	0	0	0	0	0	0	0
0	0	0	0	0	0	0	0	0
0	0	0	0	0	0	0	0	0
0	0	0	0	0	0	0	0	0
0	0	0	0	0	0	0	6	0
0	2	0	0	0	0	0	0	0
0	0	0	0	0	0	0	0	0
0	0	0	0	0	0	0	0	0
0	0	0	0	0	0	0	0	0
0	0	0	4	0	0	0	0	0
0	0	0	0	0	0	0	0	0
0	0	0	0	0	0	0	0	0
0	0	0	0	0	0	0	0	0
0	0	0	0	0	0	0	0	0

0	0	0	0	0	0	0	0	0
0	0	0	0	0	0	0	0	0
0	0	0	0	0	0	0	0	0
0	0	0	0	0	0	0	0	0
0	0	0	0	0	0	0	0	0
0	0	0	0	0	0	0	0	0
0	0	0	0	0	0	0	0	0
0	0	0	0	0	0	0	0	0
0	0	0	0	0	0	0	0	0
0	0	0	0	0	0	0	0	0
0	0	0	0	0	0	0	0	0
0	0	0	0	0	0	0	0	0
0	0	0	0	0	0	0	0	0
0	0	0	0	0	0	0	0	0
0	0	0	0	0	2	0	0	0
0	0	0	0	0	0	0	0	0
0	0	0	0	0	0	0	0	0
0	0	0	0	0	0	0	0	0
0	0	0	0	0	0	0	0	0
0	0	0	0	0	0	0	0	0
0	0	0	0	0	0	0	4	0
0	0	0	0	0	0	0	0	0
0	0	0	0	0	0	0	0	0
0	0	0	0	0	0	0	0	0
0	0	0	0	0	0	0	0	0
0	0	0	0	0	0	0	0	0
0	0	0	0	0	0	0	0	0
0	0	0	0	0	0	0	0	0
0	0	0	0	0	0	0	0	0
0	0	0	0	0	0	0	0	0
0	0	0	0	0	0	0	0	0
0	0	0	0	0	0	0	0	0
0	0	0	0	0	0	0	0	0
0	0	0	0	0	0	0	0	0
0	0	0	0	0	0	0	0	0
3	0	0	0	0	0	0	0	0
0	0	0	0	0	0	0	0	0
0	0	0	0	0	0	0	0	0
0	0	0	0	0	0	0	0	0
0	0	0	0	0	0	0	0	0
0	0	0	0	0	0	0	0	3
0	0	0	0	0	0	0	0	0
0	0	0	0	0	0	0	0	0

0	2	0	0	0	0	0	0	0
0	0	0	0	0	0	0	0	0
0	0	0	0	0	0	0	0	0
0	0	0	0	0	0	0	0	0
0	0	0	0	0	0	0	0	0
0	0	0	0	0	0	0	0	0
0	0	0	0	0	0	0	0	0
0	0	0	0	2	0	0	0	0
0	0	0	0	0	0	0	0	0
0	0	0	0	0	0	0	0	0
0	0	0	0	0	0	0	0	0
0	0	0	0	0	0	0	0	0
0	0	0	0	0	0	0	0	0
0	0	0	0	0	0	0	0	0
0	0	0	0	0	0	0	2	0
0	0	0	0	0	0	0	0	0
0	0	0	0	0	0	0	0	0
0	0	0	0	0	0	0	0	0
0	0	0	0	0	0	0	0	0
0	0	0	0	2	0	0	0	0
0	0	0	0	0	0	0	0	0
0	0	0	0	0	0	0	0	0
0	0	0	0	0	0	0	0	0
0	0	0	0	0	0	0	0	0
0	0	0	0	0	0	0	0	0

LC90	LC91	LC24	LC26	LC27	LC28	LC29	LC30	LC31
7415	23512	25366	4703	6900	20640	28509	11251	20291
1020	131	71	16956	6530	2507	101	87	108
134	407	2013	1652	479	1298	10840	2925	842
443	36	195	1927	20457	190	225	262	81
36400	20	0	39	106	62	46	171	587
0	9	4870	246	122	111	96	9722	550
378	2648	2882	524	136	306	385	1153	740
615	75	793	215	489	566	562	4838	201
1752	0	2742	1682	120	578	3426	1129	742
141	1337	426	22	935	51	150	84	237
7794	20	15	411	20	2287	201	15	0
2421	23	108	793	33	55	2395	37	5853
5184	2184	528	35	118	184	31	915	379
1370	12	459	708	2105	438	184	202	85
568	35	18	99	2536	53	76	0	14
1557	22	774	108	1152	327	120	2690	164
8	173	0	0	26	0	877	47	24
190	0	360	722	0	27	740	3161	15
123	13	32	1210	1562	2352	3283	67	78
14	6	0	411	7	95	0	6612	20
28	14	256	92	54	18	2	1078	169
0	88	2649	95	38	38	5	362	221
549	0	522	13	6599	18	0	98	101
0	33	0	21	0	0	0	0	14
54	0	159	66	0	33	961	315	1714
0	0	1621	16	0	5	0	3918	0
547	0	130	214	46	68	32	256	107
0	0	3	1248	5	2587	566	4	3
148	0	81	4	6	3	12	3	44
6	0	698	171	18	0	45	654	1001
24	8	26	86	348	896	180	1017	49
0	13	156	222	7	59	17	1288	0
0	0	19	42	0	31	524	266	376
0	0	0	0	17	0	0	0	1118
63	1604	0	0	0	0	0	802	63
110	0	90	165	17	194	143	246	7
0	0	163	40	0	17	0	407	91
0	0	111	17	0	12	0	347	43
425	0	138	13	8	453	71	802	33
190	9	205	15	37	44	60	338	42
0	0	291	23	0	16	0	1244	257
18	0	187	4	0	0	12	2	3

0	0	9	10	0	0	0	116	15
0	24	1483	63	404	547	0	0	0
242	0	170	86	0	87	42	547	31
162	0	101	47	434	22	60	585	40
0	0	205	0	0	0	93	524	164
118	18	119	15	9	0	17	205	0
14	0	0	2810	0	0	0	3	0
0	0	2466	0	0	0	0	3	0
388	0	3	40	337	399	134	128	34
0	0	158	25	0	23	0	476	0
0	0	67	56	0	104	234	23	18
0	15	8	0	10	0	0	16	8
212	0	21	17	35	67	124	74	16
506	0	23	0	317	0	42	23	101
7	0	77	0	0	11	79	378	247
0	0	50	0	0	0	0	0	0
0	0	83	28	0	138	0	159	6
0	0	30	0	1089	0	298	247	0
25	0	64	170	635	183	32	36	0
242	0	69	6	0	52	34	146	33
0	123	49	159	259	20	36	12	13
14	0	63	26	0	0	0	248	10
11	0	0	9	95	12	0	0	0
0	0	106	0	0	0	202	11	15
8	0	0	0	0	0	8	0	0
0	2510	0	0	29	0	9	8	4
0	0	0	0	183	43	0	5	0
0	7	0	11	213	19	32	39	0
93	0	0	0	0	22	36	170	34
0	0	0	0	0	0	0	0	0
0	0	0	52	0	59	0	69	12
0	0	0	0	0	0	0	5286	0
76	13	5	0	112	0	0	0	8
0	7	0	4	6	0	0	9	10
0	0	0	9	0	4	0	0	0
0	0	0	0	200	0	0	0	23
5	0	189	5	32	0	7	32	0
0	0	20	61	106	0	0	0	0
37	0	27	0	11	0	26	77	0
0	0	0	0	7	12	124	48	5
0	0	31	8	0	33	13	71	0
175	0	0	4	0	0	11	9	0
67	71	15	17	21	35	12	36	15

0	0	11	0	0	0	0	0	0
0	5	83	0	0	0	0	0	0
0	0	78	0	0	0	0	9	267
37	0	247	0	0	0	0	320	0
10	0	0	0	0	0	0	5	0
12	0	0	0	538	0	9	41	47
0	0	0	62	0	247	0	0	0
16	15	38	24	23	40	18	13	26
0	0	0	0	0	0	0	0	26
51	0	7	0	0	0	0	47	0
0	0	0	0	13	10	0	0	0
0	0	14	0	0	19	0	15	10
0	0	0	0	0	5	0	0	0
0	0	6	10	0	0	0	60	0
0	0	0	0	0	0	0	93	0
16	0	0	0	0	2	0	310	0
0	0	128	30	0	0	0	13	0
0	0	0	0	0	0	0	0	0
41	17	10	0	12	9	0	22	0
0	0	0	0	0	0	0	0	0
312	0	4	0	0	0	0	0	4
0	0	0	0	0	0	0	0	0
0	0	0	0	0	0	0	13	0
0	124	0	0	0	0	0	18	6
0	5	0	0	0	0	0	10	0
0	0	69	0	0	9	0	60	0
4	10	26	18	8	14	15	30	6
0	0	0	0	0	0	0	19	0
9	0	0	0	0	0	4	6	21
9	0	7	0	0	0	3	0	0
0	0	0	0	0	10	0	0	0
24	0	8	0	0	0	0	0	20
0	0	0	0	0	0	17	0	0
0	0	0	0	0	0	0	0	0
0	0	0	0	0	0	0	0	0
0	0	17	0	0	0	0	27	6
0	213	0	0	23	0	0	0	0
19	0	0	13	0	0	0	0	0
0	0	0	0	0	0	0	0	0
0	0	0	0	18	0	0	0	0
0	3	0	0	0	0	0	0	0
0	0	32	0	0	0	0	19	0
0	0	0	0	0	0	0	0	11

34	0	0	0	0	0	0	0	0
0	0	13	0	0	13	0	39	0
0	0	21	0	10	0	0	4	0
8	0	0	0	8	0	0	0	0
0	0	0	0	0	0	0	0	0
0	0	0	0	0	0	0	0	0
0	0	7	0	0	8	0	94	0
0	0	0	0	0	0	0	0	0
0	10	0	0	0	8	11	0	8
0	0	0	0	0	0	0	0	0
0	0	0	0	38	0	0	6	0
0	0	0	5	24	14	17	5	0
7	0	0	0	0	0	0	0	0
23	0	0	0	0	9	0	0	0
0	0	0	0	0	0	0	0	0
0	0	0	0	0	0	0	0	0
5	0	0	0	0	0	0	0	0
0	0	0	0	0	0	0	33	0
0	0	4	0	0	0	0	0	0
0	0	3	0	0	0	9	2	0
0	0	0	0	0	0	0	9	0
0	0	0	0	0	0	0	0	0
27	0	0	0	0	0	0	22	0
0	0	0	0	0	0	0	0	0
0	0	0	0	0	0	0	0	0
0	0	0	3	7	0	0	7	0
0	0	0	0	0	0	0	6	0
9	0	5	0	38	0	0	0	0
0	0	0	0	0	0	0	0	0
0	0	0	0	0	0	0	4	0
0	0	0	0	0	0	0	0	0
45	0	0	0	0	6	0	0	0
0	0	0	0	0	0	0	3	0
0	0	0	0	0	0	0	6	0
0	0	0	0	0	0	0	0	0
0	0	10	10	6	7	0	0	0
0	0	0	0	0	0	0	0	0
0	0	0	0	0	0	0	0	0
0	0	0	0	0	0	0	0	0
0	0	5	0	0	0	0	0	0
0	0	0	0	0	0	0	0	0
0	0	0	0	0	0	0	0	0
0	0	0	0	0	0	0	0	0

0	0	3	0	0	0	0	0	0
0	0	0	0	0	0	0	0	0
24	0	0	3	2	7	0	0	0
0	0	0	0	0	0	0	0	0
32	0	0	0	0	0	0	11	0
0	0	0	0	0	0	0	0	0
0	0	0	0	0	0	0	0	0
0	0	0	0	17	0	0	0	0
0	0	3	0	0	0	0	9	0
0	0	0	0	0	0	0	20	0
0	0	0	0	0	0	0	0	0
0	0	0	0	0	0	0	0	0
0	0	0	0	4	0	12	0	0
0	0	0	0	0	0	0	0	0
0	0	0	0	0	0	0	0	0
0	0	0	0	0	0	0	0	0
0	0	0	0	0	0	0	0	0
0	0	0	0	0	0	5	0	0
0	0	0	0	0	0	0	0	0
0	0	0	0	0	0	0	0	0
0	0	0	0	0	0	0	0	0
0	0	0	0	0	0	0	0	0
0	0	0	0	0	0	0	0	0
0	0	0	0	0	0	0	0	0
39	0	0	0	0	0	0	0	0
0	0	0	0	0	4	0	0	0
0	0	0	0	0	0	0	0	0
0	0	0	0	0	0	0	0	0
0	0	0	0	0	0	0	0	0
0	0	0	0	0	0	0	0	0
0	0	0	0	0	0	3	0	0
0	0	0	0	0	0	0	0	0
0	0	0	0	0	0	0	6	0
0	0	0	0	0	0	0	0	0
0	0	0	0	0	0	0	0	0
0	0	0	0	0	0	0	0	0
0	0	0	0	0	0	0	0	0
21	0	0	0	0	0	0	9	0
0	0	0	0	0	0	0	0	0
0	0	0	0	0	0	0	0	0
0	0	2	0	0	0	0	0	0
0	0	0	0	0	0	0	0	0
0	0	5	0	0	0	0	7	0

0	0	0	0	0	0	0	0	0
0	0	0	0	0	0	0	0	0
0	0	0	0	0	0	0	0	0
0	0	0	0	0	0	0	5	0
0	0	0	0	0	0	0	22	0
13	0	0	0	8	7	0	6	0
0	0	0	0	0	0	0	0	0
0	0	0	0	0	0	0	0	0
0	0	0	0	0	0	0	0	0
0	0	0	0	0	0	0	0	0
0	0	0	0	0	0	0	0	0
0	0	0	0	0	0	0	0	0
0	0	0	0	0	0	0	0	0
0	0	0	0	0	0	0	0	0
0	0	0	0	0	0	0	0	0
0	0	0	0	0	0	0	0	0
0	0	0	0	0	0	0	0	0
0	8	0	0	0	0	0	0	0
0	0	0	0	0	0	0	0	0
0	0	0	0	0	0	0	0	0
0	0	0	0	0	0	0	0	0
0	0	0	0	0	0	0	0	0
0	0	0	0	0	0	0	0	0
0	0	0	0	0	0	0	0	0
0	0	0	0	0	0	0	0	0
0	0	0	0	0	0	0	0	0
0	0	0	0	0	0	0	0	0
0	0	0	0	0	0	0	0	0
0	0	0	0	0	0	0	0	0
0	0	0	0	0	0	0	0	0
0	0	0	0	0	0	0	0	0
0	0	0	0	0	0	0	0	0
0	0	0	0	0	0	0	0	0
0	0	0	0	0	0	0	0	0
0	0	0	0	0	0	0	0	0
0	0	0	0	0	0	0	0	0
0	0	0	0	0	0	0	0	0
0	0	0	0	0	0	0	0	0
0	0	0	0	0	0	0	0	0
0	0	0	0	0	0	0	0	0
0	0	0	0	0	0	0	0	0
0	0	0	0	0	0	0	0	0
0	0	0	0	0	0	0	0	0

0	0	0	0	0	0	0	0	0
0	0	0	0	0	0	0	0	0
0	0	0	0	0	0	0	0	0
0	0	0	0	0	0	0	0	0
0	0	0	0	0	0	0	0	0
0	0	0	0	0	0	0	0	0
0	0	0	0	0	0	0	0	0
0	0	0	0	0	0	0	0	5
0	0	0	0	0	0	0	0	0
0	0	0	0	0	0	0	0	0
0	0	0	0	0	0	0	0	0
0	0	0	0	0	0	0	0	0
0	0	0	0	0	0	0	0	0
0	0	0	0	0	0	0	0	0
0	0	0	0	0	0	0	0	0
0	0	0	0	0	0	0	0	0
0	0	0	0	0	4	0	0	0
0	0	0	0	0	0	0	0	0
0	0	0	0	0	0	0	0	1
0	0	0	0	0	0	0	0	0
0	0	0	0	0	0	0	0	0
0	0	0	0	0	0	0	0	0
0	0	0	0	0	0	0	0	0
0	0	0	0	0	0	0	0	0
0	0	0	0	0	0	0	0	0
0	0	0	0	0	0	0	0	0
0	0	0	0	0	0	0	0	0
0	0	0	0	0	0	0	0	0
0	0	0	0	0	0	0	0	0
0	0	0	0	0	0	0	0	0
0	0	0	0	0	0	0	0	0
0	0	0	0	0	0	0	0	0
0	0	0	0	0	0	0	0	0
0	0	0	0	0	0	0	0	0
0	0	0	0	0	0	2	0	0
0	0	0	0	0	0	0	0	0
0	0	0	0	0	0	0	0	0
0	0	0	0	0	0	0	0	0
0	0	0	0	0	0	0	0	0
0	0	0	3	0	0	0	3	0
0	0	0	0	0	0	0	0	0
0	0	0	0	0	0	0	0	0
0	0	0	0	0	0	0	0	0

0	0	0	0	0	0	0	0	0
0	0	0	0	0	0	0	0	0
0	0	0	0	0	0	0	0	0
0	0	0	0	0	0	0	0	0
0	0	0	0	0	0	0	0	0
0	0	0	0	0	0	0	0	0
0	0	0	0	0	0	0	0	0
0	0	0	0	0	0	0	0	0
0	0	0	0	0	0	0	0	0
0	0	0	0	0	0	0	0	0
0	0	0	0	0	0	0	0	0
0	0	0	0	0	0	0	0	0
0	0	0	0	0	0	0	0	0
0	0	0	0	0	0	0	0	0
0	0	0	0	0	0	0	0	0
0	0	0	0	0	0	0	0	0
0	0	0	0	0	0	0	0	0
0	0	0	0	0	0	0	0	0
0	0	0	0	0	0	0	0	0
0	0	0	0	0	0	0	0	0
0	0	0	0	0	0	0	0	0
0	0	0	0	0	0	0	0	0
0	0	0	0	0	0	0	0	0
0	0	0	0	0	0	0	0	0
0	0	0	0	0	0	0	0	0
0	0	0	0	0	0	0	0	0
0	0	0	0	0	0	0	0	0
0	0	0	0	0	0	0	0	0
0	0	0	0	0	0	0	0	0
0	0	0	0	0	0	0	0	0
0	0	0	0	0	0	0	0	0
0	0	0	0	0	0	0	0	0
0	0	0	0	0	0	0	0	0
0	0	0	0	0	0	0	0	0
0	0	0	0	0	0	0	0	0
0	0	0	0	0	0	0	0	0
0	0	0	0	0	0	0	0	0
0	0	0	0	0	0	0	0	0
0	0	0	0	0	3	0	0	0
0	0	0	0	0	0	0	0	0
0	0	0	0	0	0	0	0	0
0	0	3	0	0	0	0	0	0
0	0	0	0	0	0	0	0	0

0	0	0	0	0	0	0	0	0
0	0	0	0	0	0	0	0	0
0	0	0	0	0	0	0	0	0
0	0	0	0	0	0	0	0	0
0	0	0	0	0	0	0	0	0
0	0	0	0	0	0	0	0	0
0	0	0	0	0	0	0	0	0
0	0	0	0	0	0	0	0	0
0	0	0	0	0	0	0	0	0
0	0	0	0	0	0	0	0	0
0	0	0	0	0	0	0	0	0
0	0	0	0	0	0	0	0	0
0	0	0	0	0	0	0	0	0
0	0	0	0	0	0	0	0	0
0	0	0	0	0	0	0	0	0
0	0	0	0	0	2	0	0	0
2	0	0	0	0	0	0	0	0
0	0	0	0	0	0	0	0	0
0	0	0	0	0	0	0	0	0
0	0	0	0	0	0	0	0	0
0	0	0	0	0	0	0	0	0
0	0	0	0	0	0	0	0	0
0	0	0	0	0	0	0	0	0
0	0	0	0	0	0	0	0	0
0	0	0	0	0	0	0	0	0

LC32	LC33	LC37	LC38	LC39	LC40	LC41	LC42	LC44
18361	2268	3786	10643	46732	192	23744	19109	9198
84	65	23203	14617	25	24	3199	28	5084
1860	1381	1320	2711	6499	29	1130	1312	3638
6061	139	84	101	1858	21	19	239	29
85	22112	13	295	159	0	1431	0	247
8	13	1348	269	95	10	14002	6480	3465
36	822	1664	492	3107	28552	3815	196	635
392	617	561	1041	917	90	315	261	594
1253	976	598	856	20	0	222	565	4035
2951	3111	295	18	0	11	1238	113	156
10	51	0	15	0	5	8	0	0
353	77	47	57	131	180	194	651	293
128	1596	47	497	81	23599	119	47	670
3292	4418	553	173	2469	0	67	62	69
15	685	0	17	717	7	0	0	0
404	502	291	537	822	11	2223	161	1608
0	520	38	89	14	271	0	0	0
255	247	1517	2139	2425	0	487	618	601
974	1499	47	361	135	0	93	6	22
0	0	1290	175	0	0	1577	289	3547
0	7	680	195	19	4	512	541	4588
1286	405	204	305	0	7	903	418	409
8	250	0	42	0	0	38	399	7
59	16	32	24	0	166	0	0	0
86	0	335	477	78	0	496	350	468
0	0	538	32	0	0	0	0	164
125	142	81	988	1098	0	369	0	5428
4	0	264	328	10	0	0	58	120
152	0	1537	0	0	3	380	18	0
3	3	73	806	0	0	24	1383	413
723	572	139	249	0	0	245	14	465
0	900	319	78	0	0	1087	29	364
542	0	50	809	0	11	145	23	547
0	0	0	0	0	0	1442	392	0
4	3611	67	0	270	862	37	0	795
31	125	210	981	81	0	147	0	371
236	73	425	192	0	0	1459	17	238
0	0	191	44	0	0	1438	63	332
310	329	88	125	0	0	343	14	77
135	439	47	34	512	0	925	0	122
0	6	0	76	0	4	1724	202	306
0	0	196	0	0	0	127	25	503

0	0	199	28	0	0	47	83	167
0	0	1012	109	0	0	0	0	105
121	335	79	118	0	0	353	19	411
206	80	86	129	0	0	365	40	286
1199	62	72	68	0	0	140	47	159
9	7	6	4	65	0	166	0	0
0	0	0	0	40	0	0	0	0
4	0	33	0	0	0	462	0	0
0	325	224	62	1327	0	228	6	59
0	0	30	26	0	0	484	0	418
165	256	60	30	454	0	0	27	0
38	0	6	4	129	477	0	0	0
95	411	25	47	110	0	47	26	73
13	0	0	260	35	0	3	26	7
766	0	28	15	0	2	92	40	21
0	0	95	0	0	0	171	0	969
0	0	76	50	0	0	117	0	285
26	0	0	69	114	154	63	13	8
0	4	39	58	22	0	0	0	11
86	62	35	69	0	0	8	34	17
568	8	9	0	249	0	0	0	0
0	0	47	89	0	0	78	20	368
38	0	0	0	0	0	0	0	0
0	0	0	524	0	0	114	19	119
9	0	0	0	0	0	0	0	0
31	0	0	0	30	86	9	0	0
435	48	0	4	73	0	31	4	0
0	8	9	0	0	0	148	8	40
106	85	58	67	0	0	130	0	43
0	0	0	0	0	0	0	0	0
0	0	331	30	0	0	8	47	172
0	0	0	0	0	0	0	0	0
0	0	0	0	69	0	0	0	0
8	16	7	8	0	1638	0	5	6
0	10	0	0	0	0	0	0	0
64	18	15	13	0	0	0	19	0
0	6	32	18	0	0	80	0	51
0	0	13	21	0	0	0	5	37
10	11	13	0	43	0	47	0	5
0	0	0	0	0	0	0	21	0
0	14	0	10	0	0	9	0	149
0	0	0	0	17	0	0	0	0
30	31	0	26	8	236	4	6	12

0	595	0	0	0	0	0	0	0
0	0	0	463	18	0	64	11	2
0	0	51	82	0	0	37	58	122
0	0	14	0	0	0	0	0	0
0	0	0	461	0	0	0	0	0
60	0	26	36	16	0	32	39	0
0	0	0	10	0	3	1109	0	166
33	16	18	39	6	4	0	6	0
0	0	143	0	0	0	0	51	0
0	0	0	0	35	0	27	0	0
0	0	0	0	0	0	0	14	0
0	6	0	12	0	6	0	0	3
0	0	0	0	0	0	0	0	0
0	0	28	38	0	0	37	28	55
0	0	0	11	0	0	146	0	0
0	0	26	0	0	0	0	9	0
0	85	0	24	0	0	0	0	0
0	0	0	0	0	0	0	0	0
0	0	0	0	0	162	4	0	0
275	0	0	0	0	0	0	0	0
10	7	0	0	0	0	0	0	0
0	0	0	0	0	0	0	0	0
0	0	0	0	0	0	31	0	177
0	0	0	0	0	0	0	0	0
20	8	0	28	0	0	0	0	0
0	53	40	12	0	0	18	0	73
6	10	2	23	0	0	0	0	0
0	0	0	0	0	0	0	0	0
0	0	0	0	0	0	0	0	0
0	0	0	0	0	0	37	0	0
0	0	0	0	0	0	0	0	73
5	0	0	0	0	23	0	0	0
0	0	0	0	0	20	0	25	0
0	0	0	0	0	0	0	0	0
0	0	0	0	0	0	0	0	0
0	0	0	0	0	0	0	0	0
6	0	0	0	0	0	0	0	0
0	247	0	0	0	0	0	0	0
0	0	0	0	0	0	0	0	0
48	0	0	0	0	0	0	0	0
0	0	0	0	0	0	0	0	0
0	16	0	0	0	0	36	7	0
0	410	0	0	0	0	0	0	0

0	0	0	0	0	0	430	0	0
9	0	12	0	0	0	0	0	24
0	0	0	0	0	0	11	17	17
0	0	0	0	0	3	0	0	0
0	0	0	0	0	0	0	0	0
0	0	0	0	0	0	0	0	0
0	0	0	0	0	0	0	0	0
91	0	0	0	0	0	0	0	0
10	14	0	15	0	3	0	0	0
13	0	0	0	0	0	0	0	0
0	0	0	0	0	0	0	7	29
0	18	4	0	0	0	0	5	12
0	0	0	0	0	0	0	0	0
0	11	0	0	0	23	0	0	0
0	0	0	0	0	0	0	0	0
0	0	6	0	0	0	0	0	7
0	0	0	0	0	0	0	0	0
0	0	0	0	0	0	0	0	0
0	0	7	59	0	0	0	0	21
0	0	0	2	0	0	0	0	7
0	0	0	5	0	0	0	0	28
0	0	0	0	0	0	0	0	0
0	0	0	0	0	0	0	0	0
0	0	0	0	0	0	0	0	0
0	0	0	0	0	0	0	0	0
4	0	0	5	0	0	0	0	0
0	0	0	0	0	0	23	0	0
0	0	0	0	15	0	0	0	0
0	0	0	14	0	0	0	23	0
0	0	0	0	0	0	0	0	0
0	0	0	0	0	0	0	0	0
0	6	0	0	0	0	0	0	0
0	0	0	0	0	0	0	0	73
0	0	0	0	0	0	0	0	0
0	0	0	0	0	0	0	0	0
0	0	0	0	0	0	0	0	0
0	0	0	0	0	0	0	0	0
0	0	0	0	0	4	0	0	0
0	0	0	0	0	0	0	0	0
0	0	0	0	0	0	0	0	0
0	0	0	0	0	0	0	0	0
0	0	0	0	0	0	0	0	0
0	0	0	0	0	0	0	0	0

0	0	0	0	0	0	0	0	0
0	0	0	0	0	0	4	0	0
0	0	0	0	0	3	6	0	0
0	0	0	0	0	0	22	0	0
0	0	0	0	0	0	0	0	0
0	0	0	0	0	0	0	0	0
0	0	0	0	0	0	0	0	0
0	1	0	0	0	31	0	0	0
0	0	0	0	0	0	0	0	0
0	0	0	0	0	0	0	0	0
0	0	0	27	0	0	0	0	0
0	0	0	0	0	0	0	0	0
0	0	0	0	0	0	0	0	0
0	0	0	0	0	0	0	0	0
0	0	0	0	0	0	0	0	0
0	0	0	0	0	0	0	0	0
0	0	0	5	0	0	6	0	0
0	0	0	0	0	0	0	0	0
0	0	0	0	0	0	0	0	0
0	0	0	0	0	0	0	0	0
0	0	0	0	0	0	0	0	0
0	0	0	0	0	0	0	0	0
0	0	0	0	0	0	0	0	0
0	0	0	0	0	0	0	0	4
0	0	0	0	0	0	0	0	0
0	0	0	0	0	0	0	0	0
0	0	0	0	0	0	0	0	0
0	0	0	0	6	0	0	0	0
0	0	0	0	0	0	0	0	0
6	0	0	0	0	0	0	0	0
0	0	0	0	0	0	0	0	0
0	0	0	0	0	0	0	0	0
0	0	0	11	0	0	0	0	0
0	0	0	0	0	0	0	0	0
0	0	0	0	0	0	0	0	0
0	0	0	0	0	0	0	0	0
0	0	0	0	0	0	0	0	0
0	0	0	0	0	0	18	0	0
0	0	0	0	0	0	0	0	0
0	0	0	0	0	0	0	0	0
0	0	0	0	0	0	13	0	14
0	0	0	0	0	0	0	0	0
0	0	0	0	0	0	0	0	0

0	0	0	0	0	0	0	0	0
0	0	0	0	0	0	0	0	0
0	0	0	0	0	0	0	0	0
0	0	0	0	0	0	0	0	0
0	0	0	0	0	0	0	0	0
0	0	0	0	0	0	0	0	0
0	0	0	0	0	0	0	0	0
0	0	0	5	0	0	0	0	0
0	0	0	0	0	0	0	0	0
0	0	0	0	0	0	0	0	0
0	0	0	0	0	0	0	0	0
0	0	0	0	0	0	0	0	0
0	0	0	0	0	0	0	0	0
0	0	0	0	0	0	0	0	0
28	0	0	0	0	0	0	0	0
0	0	0	0	28	0	0	0	0
0	0	0	0	0	0	0	0	0
0	0	0	0	0	0	0	0	0
0	0	0	0	0	0	0	0	0
0	0	0	0	0	0	0	0	0
0	0	0	0	0	0	0	0	0
0	0	0	0	0	0	0	0	0
0	0	0	0	0	0	0	0	0
0	0	0	0	0	0	0	0	0
0	0	0	0	0	0	0	0	0
0	0	0	0	0	0	0	0	0
0	0	0	0	0	0	0	0	0
0	0	0	0	0	0	0	0	2
0	8	0	0	0	0	0	0	0
0	0	0	0	0	0	0	0	0
0	0	0	0	0	0	0	0	0
0	0	0	0	0	0	0	0	0
0	0	0	0	0	0	0	0	0
0	0	0	0	0	0	0	0	0
0	0	0	0	0	0	0	0	0
0	0	0	0	0	0	0	0	0
0	0	0	0	0	0	0	0	0
0	0	0	0	0	0	0	0	0
0	0	0	0	0	0	0	0	0
0	0	0	0	0	0	0	0	0
0	0	0	0	0	0	0	0	0
0	0	0	0	0	0	0	0	0
0	0	0	0	0	0	0	0	0

0	0	0	5	0	0	0	0	0
0	0	0	0	0	0	0	0	0
0	0	0	0	0	0	0	0	0
0	0	0	0	0	0	0	0	0
0	0	0	0	0	0	0	0	0
0	0	0	0	0	0	0	0	0
0	0	0	0	0	0	0	0	0
0	0	0	0	0	0	0	0	0
0	0	0	0	0	0	0	0	0
0	0	0	0	0	0	0	0	0
0	0	0	0	0	0	0	0	0
0	0	0	0	0	0	0	0	0
0	0	0	0	0	0	0	0	0
0	0	0	0	0	0	0	0	0
0	0	0	0	0	0	0	0	0
0	0	0	0	0	0	0	0	0
0	0	0	0	0	0	0	0	0
0	0	0	0	0	0	0	0	0
0	0	0	7	0	0	0	0	0
0	0	0	0	0	0	0	0	0
0	0	0	0	0	0	0	0	0
0	0	0	0	0	0	0	0	0
0	0	0	0	0	0	0	0	3
0	0	0	0	0	0	0	0	0
0	0	0	0	0	0	5	0	0
0	0	0	0	0	0	0	0	0
0	0	0	0	0	0	0	0	0
0	0	0	0	0	0	0	0	0
0	0	0	0	0	0	0	0	0
0	0	0	0	0	0	0	0	0
0	0	0	0	0	0	0	0	0
0	0	0	0	0	0	0	0	0
0	0	0	0	0	0	0	0	0
0	0	0	0	0	0	0	0	0
0	0	0	0	0	0	0	0	0
0	0	0	0	0	0	0	0	0
0	0	0	0	0	0	0	0	0
0	0	0	0	0	0	0	0	0
0	0	0	0	0	0	0	0	0
0	0	0	0	0	0	0	0	0
0	0	0	0	0	0	0	0	0
0	0	0	0	0	0	0	0	0
0	0	0	0	0	0	0	0	0

0	0	0	0	0	0	0	0	0
0	0	0	0	0	0	0	0	0
0	0	0	0	0	0	0	0	0
0	0	0	0	0	0	0	0	0
0	0	0	0	0	0	0	0	0
0	0	0	0	0	0	0	0	0
0	0	0	0	0	0	0	0	0
0	0	0	0	0	0	0	0	0
0	0	0	0	0	0	0	0	0
0	0	0	0	0	0	0	0	0
0	0	0	0	0	0	0	0	0
0	0	0	0	0	0	0	0	0
0	0	0	0	0	0	0	0	0
0	0	0	0	0	0	0	0	0
0	0	0	0	0	0	0	0	0
0	0	0	0	0	0	0	0	0
0	0	0	0	0	0	0	0	0
0	0	0	0	0	0	0	0	0
0	0	0	0	0	0	0	0	0
0	0	0	0	0	0	0	0	0
0	0	0	0	0	0	0	0	0
0	0	0	0	0	0	0	0	0
0	0	0	0	0	0	0	0	0
0	0	0	0	0	0	0	0	0
0	0	0	0	0	0	0	0	0
0	0	3	0	0	0	0	0	0
0	0	0	0	0	0	0	0	0
0	0	0	0	0	0	0	0	0
0	0	0	0	0	0	0	0	0
0	0	0	0	0	0	0	0	0
0	0	0	0	0	0	0	0	0
0	0	0	0	0	0	0	0	0
0	0	0	0	0	0	0	0	0
0	0	0	0	0	0	0	0	0
0	0	0	0	0	0	0	0	0
0	0	0	0	0	0	0	0	0
0	0	0	0	0	0	0	0	0
0	0	0	0	0	0	0	0	0
0	0	0	0	0	0	0	0	0
0	0	0	0	0	0	0	0	0
0	0	0	0	0	0	0	0	0
0	0	0	0	0	0	0	0	0
0	0	0	0	0	0	0	0	0

0	0	0	0	0	0	0	0	0
0	0	0	0	0	0	0	0	0
0	0	0	0	0	0	0	0	0
0	0	0	0	0	0	0	0	0
0	0	0	0	0	0	0	0	0
0	0	0	0	0	0	0	0	0
0	0	0	0	0	0	0	0	0
0	0	0	0	0	0	0	0	0
2	0	0	0	0	0	0	0	0
0	0	0	0	0	0	0	0	0
0	0	0	0	0	0	0	0	0
0	0	0	2	0	0	0	0	0
0	0	0	0	0	0	0	0	0
0	0	0	0	0	0	0	0	0
0	0	0	0	0	0	0	0	0
0	0	0	0	0	0	0	0	0
0	0	0	0	0	0	0	0	0
0	0	0	0	0	0	0	0	0
0	0	0	0	0	0	0	0	0
0	0	0	0	0	0	0	0	0
0	0	0	0	0	0	0	0	0
0	0	0	0	0	0	0	0	0
0	0	0	0	0	0	0	0	0
2	0	0	0	0	0	0	0	0
0	0	0	0	0	0	0	0	0

LC46	LC47	LC48	LC49	LC50	LC51	LC52	LC53	LC54
19193	28279	14246	7178	15598	7421	30895	28247	31180
45	78	58	10365	73	393	32	66	2632
12104	112	6441	7505	3860	17012	20972	1333	1264
3841	844	1208	3370	4072	96	383	372	135
238	5906	9	30	0	2803	120	0	0
250	1202	86	259	3230	1131	17	7472	3585
147	809	223	449	349	536	14	887	2310
4501	2057	942	640	734	540	125	328	187
846	0	3494	921	945	928	609	2510	662
904	113	70	19	278	11	918	83	111
0	14	0	0	10	0	0	65	0
696	100	56	43	19	281	23	23	24
141	959	28	7	32	12	9	5	32
372	1864	389	226	546	217	775	1525	169
103	285	0	44	103	23	80	18	0
564	1125	255	120	651	270	368	726	1225
0	3856	0	0	380	23	0	0	0
341	1767	65	20	1021	2228	0	68	415
129	241	1857	514	52	687	658	103	320
115	6	0	411	3853	530	6	177	1421
25	594	10	3481	174	210	12	5566	723
1256	1304	1453	42	216	220	16	180	1486
1608	2141	0	76	238	8	11	1279	17
0	0	0	0	13	0	18	32	9
588	34	300	3716	160	2904	23	950	292
0	7	0	0	0	6	6	3287	1713
2	119	107	223	107	87	102	216	143
0	18	57	8	3	11	0	0	413
0	14	8	0	0	697	853	0	0
158	186	0	216	362	277	0	2167	95
205	1263	201	141	1382	450	0	222	108
205	0	45	61	0	0	0	205	181
2201	0	1152	465	33	303	0	0	184
0	0	8	0	0	1176	0	234	1082
8	319	5	0	0	5	0	24	0
120	7	109	90	131	127	36	284	219
107	134	15	0	747	189	97	103	190
32	0	36	119	639	440	0	97	2492
305	415	90	14	350	0	155	108	643
101	103	26	23	264	30	1852	8	73
12	4	11	66	93	3	0	525	414
0	12	3	361	0	490	0	8	1484

0	0	0	30	340	42	0	120	226
0	0	0	645	0	0	0	1536	1043
372	297	78	44	66	264	0	93	500
118	200	57	26	55	54	0	104	161
1133	56	700	348	295	25	0	99	67
0	0	0	0	0	0	0	47	25
0	0	2	0	6	5	2	0	0
0	0	0	0	13	0	0	58	5
147	0	388	31	48	67	0	16	51
0	224	105	32	0	0	0	194	289
232	0	79	24	0	47	0	50	0
5	26	0	0	0	0	33	0	0
235	91	198	26	26	48	116	25	54
69	6	3	0	31	118	0	12	267
308	0	91	51	31	147	175	9	273
0	0	0	0	0	646	0	224	317
35	0	0	51	6	42	0	121	83
40	0	0	0	215	101	0	82	29
12	0	16	0	57	66	0	228	0
43	33	40	0	65	33	0	10	94
34	101	334	33	47	0	486	54	0
0	0	0	38	94	70	0	38	28
9	31	20	0	13	0	86	0	0
0	73	85	58	54	71	0	9	199
0	0	0	0	0	0	0	0	0
0	15	0	0	0	0	23	0	0
0	11	0	0	0	0	31	34	5
9	83	0	35	5	0	0	31	14
0	105	39	0	26	21	0	46	20
0	0	0	0	0	0	0	0	0
57	0	146	19	29	30	0	40	98
0	0	0	0	0	0	0	0	0
0	26	0	0	21	0	15	0	0
2	0	0	0	0	0	0	6	6
0	0	0	0	0	0	0	0	0
0	113	0	0	0	0	0	0	0
37	0	10	13	30	16	3	66	10
23	123	0	36	54	0	0	116	240
0	0	0	0	0	0	0	33	0
5	0	0	9	0	4	0	8	0
0	18	89	0	0	0	0	75	0
9	0	13	0	0	48	0	0	0
0	12	0	0	0	0	0	0	11

0	0	0	0	0	0	0	0	0
0	0	82	72	24	101	0	0	51
17	0	0	0	0	216	0	0	82
0	0	0	0	18	462	0	0	0
0	0	0	0	0	0	0	5	0
53	49	0	0	151	115	0	51	0
279	0	0	0	0	0	0	119	0
13	18	24	0	19	4	27	23	9
0	0	0	0	8	12	0	25	84
0	0	0	0	0	0	0	57	5
0	0	0	0	0	0	0	15	0
0	6	3	7	8	6	4	0	0
0	0	0	0	0	0	0	12	0
0	18	0	0	21	24	0	55	36
27	154	0	0	4	24	0	0	7
0	0	0	0	0	0	0	0	16
0	15	0	0	16	0	0	0	19
0	0	0	0	0	0	0	0	0
4	6	0	0	0	0	0	0	0
0	0	0	0	0	0	0	0	0
0	12	0	0	0	0	11	0	0
0	0	7	0	0	0	0	0	0
0	0	0	3	11	18	0	0	63
0	28	0	0	0	0	0	0	10
0	0	0	0	0	0	0	0	0
0	87	0	0	49	0	0	0	34
0	7	18	0	11	10	0	9	12
0	0	0	0	0	0	0	4	0
0	0	0	0	0	0	0	0	0
0	0	0	0	0	0	0	0	10
0	0	0	0	0	0	0	17	16
0	0	0	4	0	0	0	13	0
11	0	0	0	29	171	0	0	0
0	0	0	0	0	0	0	0	1089
0	0	0	0	0	0	0	0	3
0	0	0	18	10	112	0	17	10
0	0	0	0	24	0	7	0	0
0	5	0	0	0	0	0	0	0
0	0	0	0	0	0	0	0	0
0	0	7	0	5	0	0	0	0
0	0	0	0	0	0	0	0	0
15	0	0	6	16	0	0	0	9
0	0	0	0	0	0	0	0	0

0	0	4	0	0	0	0	0	0
0	0	0	26	0	0	0	5	62
0	0	0	0	0	0	0	0	12
0	0	0	0	0	0	0	0	0
0	0	0	0	0	0	0	0	0
0	0	0	0	0	0	0	0	0
0	77	2	0	0	0	0	0	5
0	0	0	0	0	45	0	0	0
0	0	7	19	0	0	0	0	5
0	0	0	0	0	0	0	0	0
0	0	0	0	0	0	68	0	0
0	0	6	6	11	0	0	13	0
0	0	0	0	0	0	0	0	0
0	4	0	0	0	0	0	0	0
0	0	0	0	0	0	0	0	0
0	0	0	0	0	0	0	0	0
0	0	0	0	0	0	0	0	0
0	0	0	0	0	0	0	0	0
0	0	0	0	4	8	0	0	5
0	0	0	0	0	9	0	5	0
40	0	0	6	0	7	0	51	0
0	8	8	8	2	3	0	7	2
0	0	4	0	0	0	2	0	2
0	0	0	0	0	0	0	0	0
0	0	0	0	0	0	0	0	0
0	0	0	5	2	0	0	0	0
0	0	0	0	0	0	0	0	0
0	21	0	0	0	0	0	10	0
0	0	0	0	0	0	0	23	52
0	0	0	0	0	0	0	0	0
0	0	5	0	0	0	0	0	0
0	0	0	7	0	0	0	0	0
0	0	0	0	0	35	0	0	0
0	0	0	0	0	0	0	0	0
0	0	0	0	0	0	0	0	0
0	0	0	0	0	0	0	0	0
0	0	0	0	0	0	0	0	0
0	0	0	0	0	0	0	0	0
0	0	0	0	0	0	0	0	0
0	0	0	0	0	0	0	0	0
0	0	0	0	0	0	0	0	0
0	0	0	0	0	0	0	16	0
0	0	0	0	0	0	0	0	7

0	0	0	3	4	0	0	0	0
0	0	0	0	0	0	0	0	0
0	0	0	0	0	0	0	0	0
0	0	12	0	0	0	0	0	0
0	0	0	0	0	0	0	0	0
0	0	0	0	0	0	0	0	0
0	0	0	0	0	0	0	0	0
0	0	0	0	0	0	0	0	0
0	0	0	0	0	0	0	0	0
0	0	0	0	0	0	0	0	0
28	0	0	0	0	0	0	0	0
0	0	0	0	0	0	0	0	0
0	15	0	0	0	0	0	0	0
0	0	0	0	0	0	0	0	0
0	0	0	0	0	0	0	0	0
0	0	0	0	0	0	0	0	0
0	0	0	0	0	0	0	0	0
0	0	0	0	0	0	0	0	0
0	0	0	0	0	0	0	0	0
0	0	0	3	3	0	0	0	0
0	0	0	0	0	0	0	0	0
0	0	0	0	0	0	0	0	0
0	0	0	0	0	0	0	0	0
0	0	0	0	0	0	0	11	0
0	0	0	0	0	0	0	0	0
0	0	0	0	0	0	0	0	0
0	0	0	0	0	0	0	0	0
0	12	0	0	0	0	0	0	0
0	0	0	0	0	0	0	0	0
0	0	0	0	0	0	0	0	0
0	0	0	0	0	0	0	0	0
16	0	0	8	0	0	0	0	0
0	0	0	0	0	0	0	0	4
7	0	0	0	0	0	0	0	0
0	0	0	0	0	0	0	0	0
0	0	0	0	0	0	0	0	0
0	0	0	0	0	0	0	0	0
0	0	0	0	0	0	0	0	0
0	0	0	0	0	0	0	0	0
0	0	0	0	0	0	0	0	0
0	0	0	0	0	0	0	6	5
0	0	0	0	0	0	0	0	7
0	0	0	0	0	0	0	0	0

0	0	0	0	0	0	0	0	0
0	0	5	6	0	0	0	10	0
0	0	0	0	0	0	0	0	0
0	0	0	0	0	0	0	0	0
0	0	0	0	0	0	0	0	0
0	0	0	0	0	0	0	0	0
0	0	0	0	0	0	0	0	0
0	0	0	0	0	0	0	0	0
0	0	0	0	0	0	0	3	0
0	0	0	0	0	0	0	0	0
0	0	0	0	0	0	0	0	0
0	0	0	0	0	0	0	0	0
0	0	0	0	0	0	0	0	0
0	0	0	0	0	0	0	0	0
0	0	0	0	0	0	0	0	0
0	0	0	0	0	0	0	0	0
0	0	0	0	0	0	0	0	0
0	0	0	0	0	0	0	0	0
0	0	0	0	0	0	0	0	0
0	0	0	0	0	0	0	0	0
0	0	0	0	0	0	0	0	0
0	0	0	0	0	0	0	0	0
0	0	0	0	0	0	0	0	0
0	0	0	0	0	0	0	0	0
0	0	0	0	0	0	0	0	0
0	0	0	0	0	0	0	0	0
0	0	0	0	0	0	0	0	0
0	0	0	0	0	0	0	0	0
0	0	0	0	0	0	0	0	0
0	0	0	0	0	0	0	0	0
0	0	0	0	0	0	0	0	0
0	0	0	0	0	0	0	0	0
0	0	0	0	0	0	0	0	0
0	0	0	0	0	0	0	0	0
0	0	0	0	0	0	0	0	0
0	0	0	0	0	0	0	0	0
0	0	0	0	0	0	0	0	0
0	0	0	0	0	0	0	0	0
0	0	0	0	0	0	0	0	0
0	0	0	0	0	0	0	0	0
0	0	0	0	0	0	0	0	0
0	0	0	0	0	0	0	0	0
0	0	0	0	0	0	0	0	0

0	0	0	0	0	0	0	0	0
0	0	0	0	0	0	0	0	0
0	0	0	0	0	0	0	0	0
0	0	0	0	0	0	0	0	0
0	0	0	0	0	0	0	0	0
0	0	0	0	0	0	0	0	0
0	0	0	0	0	0	0	0	0
0	0	0	0	0	0	0	0	0
0	0	0	0	0	0	0	0	0
0	0	0	0	0	0	0	0	0
0	0	0	0	0	0	0	0	0
0	0	0	0	0	0	0	0	0
0	0	0	0	0	0	0	0	0
0	0	0	0	0	0	0	0	0
0	0	0	0	0	0	0	0	0
0	0	0	0	0	0	0	0	2
0	0	0	0	0	0	0	0	0
0	0	0	0	0	0	0	0	0
0	0	0	0	0	0	0	0	0
0	0	0	0	0	0	0	0	0
0	0	0	0	0	0	0	0	0
0	0	0	0	0	0	0	0	0
0	0	0	0	0	0	0	0	0
0	0	0	0	0	0	0	0	0
0	0	0	0	0	0	0	0	0
0	0	0	0	0	0	0	0	0
0	0	0	0	0	0	0	0	0
0	0	0	0	0	0	0	0	0
0	0	0	0	0	0	0	0	0
0	0	0	0	0	0	0	0	0
0	0	0	0	0	0	0	0	0
0	0	0	0	0	0	0	0	0
0	0	0	0	0	0	0	0	0
0	0	0	0	0	0	0	0	0
0	0	0	0	0	0	0	0	0
0	0	0	0	0	0	0	0	0
0	0	0	0	0	0	0	0	0
0	0	0	0	0	0	0	0	0
0	0	0	0	0	0	0	0	0
0	0	0	0	0	0	0	0	0
0	0	0	0	0	0	0	0	0
0	0	0	0	0	0	0	0	0
0	0	0	0	0	0	0	0	0

0	0	0	0	0	0	0	0	0
0	0	0	0	0	0	0	0	0
0	0	0	0	0	0	0	0	0
0	0	0	0	0	0	0	0	0
0	0	0	0	0	0	0	0	0
0	0	0	0	0	0	0	0	0
0	0	0	0	0	0	0	0	0
0	0	0	0	0	0	0	0	0
0	0	0	0	0	0	0	0	0
0	0	0	0	0	0	0	0	0
0	0	0	0	0	0	0	0	0
0	0	0	0	0	0	0	0	0
0	0	0	0	0	0	0	0	0
0	0	0	0	0	0	0	0	0
0	0	0	0	0	0	0	0	0
0	0	0	0	0	0	0	0	0
0	0	0	0	0	0	0	0	0
0	0	0	0	0	0	0	0	0
0	0	0	0	0	0	0	0	0
0	0	0	0	0	0	0	0	0
0	0	0	0	0	0	0	0	0
0	0	0	0	0	0	0	0	0
0	0	0	0	0	0	0	0	0
0	0	0	0	0	0	0	0	0
0	0	0	0	0	0	0	0	0
0	0	0	0	0	0	0	0	0
0	0	0	0	0	0	0	0	0
0	0	0	0	0	0	0	0	0
0	0	0	0	0	0	0	0	0
0	0	0	0	0	0	0	0	0
0	0	0	0	0	0	0	0	0
0	0	0	0	0	0	0	0	0
0	0	0	0	0	0	0	0	0
0	0	0	0	0	0	0	0	0
0	0	0	0	0	0	0	0	0
0	0	0	0	0	0	0	0	0
0	0	0	0	0	0	0	0	0
0	0	0	0	0	0	0	0	0
0	0	0	0	0	0	0	0	0
0	0	0	0	0	0	0	0	0
0	0	0	0	0	0	0	0	0
0	0	0	0	0	0	0	0	0
0	0	0	0	0	0	0	0	0

0	0	0	0	0	0	0	0	0
0	0	0	0	0	0	0	0	0
0	0	0	0	0	0	0	0	0
0	0	0	0	0	0	0	0	0
0	2	0	0	0	0	0	0	0
0	0	0	0	0	0	0	0	0
0	0	0	0	0	0	0	0	0
0	0	0	0	0	0	0	0	0
0	0	0	0	0	0	0	0	0
0	0	0	0	0	0	0	0	0
0	0	0	0	0	0	0	0	0
0	0	0	0	0	0	0	0	0
0	0	0	0	0	0	0	0	0
0	0	0	0	0	0	0	0	0
0	0	0	0	0	0	0	0	0
0	0	0	0	0	0	0	0	0
0	0	0	0	0	0	0	0	0
0	0	0	0	0	0	0	0	0
0	0	0	0	0	0	0	0	0
0	0	0	0	0	0	0	0	0
0	0	0	0	0	0	0	0	0
0	0	0	0	0	0	0	0	0
0	0	0	0	0	0	0	0	0
0	0	0	0	0	0	0	0	0
0	0	0	0	0	0	0	0	0

LC55	LC56	LC57	LC58	LC59	LC60	LC61	LC63	LC66
4894	38673	30927	7506	10446	29009	7153	19209	5587
44	4466	38	75	25	573	24	60	8073
113	1226	4629	718	1587	4819	524	6906	3301
5984	1041	39	5902	80	57	4347	43	324
30	22	27	3671	26	42	24896	33	65
0	1226	1944	56	3101	1726	18	11	153
42	1149	1314	91	1455	2726	607	413	240
2606	318	926	176	257	1210	660	1794	1785
4594	802	385	160	145	220	324	12427	635
1256	149	452	192	805	2782	2406	9	409
45	4	0	225	0	6	549	112	9
20	12	76	24449	5	235	18	108	48
288	19	62	5767	147	80	599	25	79
514	163	178	379	24	927	1392	1290	500
15491	11	23	297	15	36	1910	14	345
3978	311	601	1124	115	2378	850	162	526
71	0	0	18	0	17	0	0	40
8813	44	63	104	25	146	567	271	589
540	49	223	7	92	32	253	1156	200
0	52	2115	116	6689	20	0	0	54
0	882	3935	17	857	9	3	12	46
7	711	4259	346	1529	940	11	573	193
10	129	6	9	17	138	6215	1138	5
11	0	0	0	12	0	0	0	0
5	49	2088	169	64	229	0	1596	4810
23	0	0	254	589	0	3	0	69
139	3537	220	8	133	270	103	82	9863
12	912	452	2	7	24	0	0	6
0	3547	751	0	102	3	0	386	269
6	519	219	4	403	823	0	174	652
0	747	218	1892	105	216	550	141	212
4	410	7	5653	10	5660	766	0	474
0	0	152	0	0	0	0	717	45
0	6	1367	0	7049	0	7	0	191
0	8	23	59	29	19	369	0	1543
81	40	162	162	471	97	63	89	2243
54	26	1164	198	156	1377	97	8	422
0	0	862	0	1246	66	0	0	42
315	43	224	0	55	646	208	31	158
567	13	81	13	100	208	549	84	131
0	51	180	0	192	375	0	0	113
5	0	1216	0	5623	25	3	14	92

5	7	55	0	11008	0	3	0	489
0	0	0	0	0	0	0	0	0
467	77	217	569	36	261	237	167	114
246	37	154	68	55	738	67	41	420
59	32	749	0	25	1059	25	45	207
2	33	13	7	4	38	0	0	9673
4	0	0	0	0	0	0	0	683
0	0	0	14	28	371	0	0	0
0	0	184	716	40	309	551	110	187
0	0	303	53	274	44	0	0	0
17	0	27	139	0	3	117	197	22
250	0	3	0	0	4	0	0	0
18	20	41	114	0	423	53	268	105
2386	50	54	0	70	0	667	0	32
288	0	148	0	237	1503	14	75	114
0	369	0	0	0	166	0	0	0
0	0	120	200	0	279	0	0	59
405	37	71	222	14	83	116	8	0
20	50	4	68	0	73	208	41	29
47	0	81	11	17	265	0	11	197
17	0	31	16	23	0	13	22	181
0	90	114	0	1374	83	0	0	29
0	0	0	8	0	0	9	0	0
0	9	251	0	52	0	0	89	40
0	0	0	0	0	0	0	0	0
0	0	0	21	0	3	0	0	0
51	0	0	0	0	0	74	155	0
0	82	4	4	104	99	6	8	0
0	0	71	0	0	221	29	0	0
0	0	0	0	0	0	0	0	0
0	0	193	0	110	0	0	6	15
0	484	0	0	0	0	0	0	11
17	0	8	10	0	100	9	17	0
11	4	0	20	13	0	0	0	0
0	0	0	0	0	0	0	0	0
0	0	0	0	0	0	0	0	0
217	46	70	101	34	134	111	5	0
0	33	12	0	75	18	5	0	0
5	8	18	3	25	12	0	4	2922
0	0	153	16	0	0	0	170	0
0	0	0	0	3	0	125	23	416
0	0	0	14	6	0	0	0	7
64	0	10	15	0	11	28	4	396

0	0	0	0	20	0	0	0	0
0	160	20	13	112	0	0	0	0
0	9	574	0	0	0	0	0	0
0	0	0	0	8	43	0	0	0
0	0	0	0	0	14	0	0	0
0	54	8	6	123	0	47	0	163
0	0	0	0	0	0	0	0	26
11	16	8	12	0	0	16	0	32
0	0	0	0	824	0	0	0	0
3	10	9	3	7	35	0	0	2029
0	0	6	0	31	61	0	0	0
0	2	0	0	0	0	6	11	0
0	12	0	0	0	105	0	0	77
0	0	30	6	152	50	0	0	32
0	0	0	0	0	0	0	0	0
0	0	0	0	123	0	0	0	0
506	0	0	0	0	31	243	0	0
0	0	0	0	0	0	0	0	0
13	0	0	20	0	8	0	0	0
0	0	0	0	0	0	0	0	0
14	0	0	92	0	0	11	0	0
0	161	0	0	0	0	0	0	43
0	0	0	0	271	0	0	0	0
0	4	0	0	57	2	0	0	0
0	0	0	0	0	0	0	0	0
0	0	0	0	0	0	85	0	0
9	0	0	9	11	12	0	6	14
1261	0	0	0	0	0	74	6	0
0	0	0	12	0	0	0	0	0
0	10	0	0	12	10	3	0	1183
0	0	0	0	0	0	0	0	48
0	0	0	2	0	0	0	0	0
0	0	9	0	584	0	0	0	0
0	0	0	0	0	0	0	0	0
0	0	0	0	0	0	0	0	126
0	0	51	0	0	0	0	27	125
0	0	0	0	0	0	0	0	0
279	0	0	0	0	65	172	0	0
0	0	0	0	0	0	0	0	0
0	0	0	0	0	0	0	0	0
0	0	0	0	0	0	0	0	0
23	0	0	0	0	15	0	13	34
0	0	0	0	0	0	0	0	0

0	0	0	0	0	0	0	0	0
0	0	0	0	7	49	0	0	85
0	0	0	0	95	0	0	0	0
36	0	0	8	0	0	0	0	0
0	0	0	0	25	0	0	0	0
0	0	0	0	0	0	0	0	0
0	0	0	0	5	0	0	0	0
0	0	0	0	0	0	0	150	0
0	0	8	4	0	8	0	0	8
0	0	0	0	0	0	0	0	0
0	0	0	0	0	0	0	0	0
7	0	0	0	0	9	19	0	5
0	0	0	0	0	0	0	0	0
0	0	0	5	0	0	0	0	0
0	0	0	0	0	0	0	0	400
0	0	22	0	0	0	0	0	0
4	0	0	0	0	0	0	0	0
0	0	2	0	0	0	0	0	0
0	0	0	0	14	47	0	0	0
0	2	9	0	0	9	7	26	0
0	0	0	0	4	0	3	5	0
0	0	0	0	0	0	0	0	0
0	0	0	0	0	0	0	0	0
0	0	0	0	0	0	0	0	259
0	0	0	0	0	0	0	0	0
0	0	0	0	0	0	0	0	4
0	0	7	0	0	9	0	0	0
0	0	0	0	0	21	0	0	0
0	0	0	0	0	18	0	0	0
0	0	0	0	0	0	0	0	0
0	0	0	7	0	0	0	0	0
0	0	0	0	0	0	0	0	0
0	0	0	0	0	0	0	0	0
0	17	0	0	50	18	0	0	0
0	0	0	0	165	0	0	0	0
0	0	0	0	0	0	0	0	0
0	0	0	0	0	0	0	0	220
0	0	0	0	0	0	0	0	0
0	0	0	0	0	7	0	0	0
0	10	0	0	0	2	0	0	0
0	0	0	0	0	0	0	0	0
0	0	0	0	0	0	0	0	0
0	0	0	0	0	15	0	0	0

0	0	0	0	0	0	0	0	0
0	0	0	0	154	0	0	0	0
0	0	0	0	0	0	0	0	0
0	0	0	0	0	0	0	0	0
4	0	0	5	0	0	0	0	0
0	0	0	0	0	0	0	0	0
0	0	0	0	0	0	0	0	0
0	0	0	12	0	0	8	0	0
0	0	0	0	0	4	0	0	0
0	0	3	0	0	8	0	0	0
0	0	0	0	0	0	0	0	0
0	0	0	0	0	0	0	0	0
0	0	0	7	0	0	0	0	0
0	0	0	0	0	0	0	0	0
0	0	0	0	0	0	0	0	0
0	0	0	0	0	0	0	0	100
0	8	11	0	15	38	0	0	0
0	0	0	0	0	0	0	8	0
0	0	0	0	0	0	0	0	0
0	0	0	0	0	14	0	0	0
0	0	0	0	0	0	0	0	0
0	0	0	0	0	5	0	0	0
0	0	0	0	0	0	0	0	0
0	0	0	0	0	0	0	0	0
0	0	0	0	0	0	0	0	0
0	0	0	0	0	0	0	0	0
0	0	0	0	0	0	0	0	0
0	4	0	0	0	0	0	13	0
0	0	0	0	0	0	0	0	50
0	0	0	0	0	0	0	0	0
0	0	0	25	0	0	0	0	0
0	0	0	0	0	0	0	0	0
0	0	0	0	0	0	0	0	0
0	0	0	0	0	0	0	0	0
0	0	0	0	0	0	0	0	0
0	0	0	0	0	0	0	0	0
0	0	0	0	0	5	2	0	0
0	0	0	0	0	0	0	0	0
0	0	0	0	0	0	0	0	0
0	0	0	0	0	0	0	0	54
0	0	0	0	0	0	0	0	0
0	0	0	0	0	0	0	0	17
0	0	0	0	0	0	0	0	0

0	0	0	0	0	0	0	0	0
0	0	0	0	0	0	0	0	0
0	0	0	0	26	0	0	0	0
0	0	0	0	0	0	0	0	0
0	0	0	0	0	0	0	0	0
0	0	0	0	0	0	0	0	0
0	0	0	0	0	0	0	0	0
0	0	0	0	0	0	0	0	0
0	0	0	0	0	0	0	0	0
0	0	0	0	0	0	0	0	0
0	0	0	0	0	0	0	0	0
0	0	0	0	0	0	0	0	0
0	0	0	0	0	0	0	0	0
5	0	0	0	0	0	0	0	12
0	0	0	0	0	0	0	0	0
0	0	0	0	0	0	0	0	0
0	0	0	0	0	0	0	0	0
0	0	0	0	0	0	0	0	0
0	0	0	0	0	0	0	0	0
0	0	0	0	0	0	0	0	0
0	0	0	0	0	0	0	0	0
0	0	0	0	0	0	0	0	0
0	0	0	0	0	4	0	0	0
5	0	0	0	0	0	0	0	0
0	0	0	0	0	4	0	0	0
21	0	0	0	0	0	0	0	0
0	0	0	0	0	0	0	0	0
0	0	0	0	0	0	0	0	0
0	0	0	0	0	0	0	0	0
0	0	0	0	0	0	0	0	0
0	0	0	0	0	0	0	0	0
0	0	0	0	0	0	0	0	0
0	0	0	0	0	0	0	0	0
0	0	0	0	0	0	0	0	0
0	0	0	0	0	0	0	0	0
0	0	0	0	0	0	0	0	0
0	0	0	0	0	0	0	0	0
0	0	0	0	0	0	0	0	0
0	0	0	0	0	0	0	0	13
0	0	0	0	0	0	0	0	0
0	0	0	0	0	0	0	0	0
0	0	0	0	0	0	0	0	0
0	0	0	0	0	0	0	0	0

0	0	0	0	0	0	0	0	0
0	0	0	0	0	0	0	0	0
0	0	0	0	0	0	0	0	0
0	0	0	0	0	0	0	0	0
0	0	0	0	0	0	0	0	0
0	0	0	0	0	0	0	0	0
0	0	0	0	0	0	0	0	0
0	0	0	5	0	0	0	0	0
0	0	0	0	0	0	0	0	0
0	0	0	0	0	0	0	0	0
0	0	0	0	0	0	0	0	0
0	0	0	0	0	0	0	0	0
0	0	0	0	0	0	0	0	0
0	0	0	0	0	0	0	0	0
0	0	0	0	0	0	0	0	0
0	0	0	0	0	0	0	0	0
0	0	0	0	0	0	0	0	0
0	0	0	0	0	0	0	0	0
0	0	0	0	0	0	0	0	0
0	0	0	0	0	0	0	0	0
0	0	0	0	0	0	0	0	0
0	0	0	0	0	0	0	0	0
0	0	0	0	0	0	0	0	0
0	0	0	0	7	0	0	0	0
0	0	0	0	0	0	0	0	0
0	0	0	0	0	0	0	0	0
0	0	0	0	0	0	0	0	0
0	0	0	0	0	0	0	0	0
0	0	0	0	0	0	0	0	0
0	0	0	0	0	0	0	0	0
0	0	0	0	0	0	0	0	0
0	0	0	0	0	0	0	0	0
0	0	0	0	0	0	0	0	0
0	0	0	0	0	0	0	0	0
0	0	0	0	0	0	0	0	0
0	0	0	0	0	0	0	0	0
0	0	0	0	0	0	0	0	0
0	0	0	0	6	0	0	0	0
0	0	0	0	0	0	0	0	0
0	0	0	0	0	0	0	0	0
0	0	0	0	0	0	0	0	0
0	0	0	0	0	0	0	0	0
0	0	0	0	0	0	0	0	0

0	0	0	0	0	0	0	0	0
0	0	0	0	0	0	0	0	0
0	0	0	0	0	0	0	0	0
0	0	0	0	0	0	0	0	0
0	0	0	0	0	0	0	0	0
0	0	0	0	0	0	0	0	0
0	0	0	0	0	0	0	0	0
0	0	0	0	0	0	0	0	0
0	0	0	0	0	0	0	0	0
0	0	0	0	0	0	0	0	0
0	0	0	0	0	0	0	0	0
0	0	0	0	0	0	0	0	0
0	0	0	0	0	0	0	0	0
0	0	0	0	0	0	0	0	0
0	0	0	0	0	0	0	0	0
0	0	0	0	0	4	0	0	0
0	0	0	0	0	0	0	0	0
0	0	0	0	0	0	0	0	0
0	0	0	0	0	0	0	0	0
0	0	0	0	0	0	0	0	0
0	0	0	0	0	0	0	0	0
0	0	0	0	0	0	0	0	0
0	0	0	0	0	0	0	0	0
0	0	0	0	0	0	0	0	0
0	0	0	0	0	0	0	0	0
0	0	0	0	0	0	0	0	0
0	0	0	0	0	0	0	0	0
0	3	0	0	0	0	0	0	0
0	0	0	0	0	0	0	0	0
0	0	0	0	0	0	0	0	0
0	0	0	0	0	0	0	0	0
0	0	0	0	0	0	0	0	0
3	0	0	0	0	0	0	0	0
0	0	0	0	0	0	0	0	0
0	0	0	0	0	0	0	0	0
0	0	0	0	0	0	0	0	0
0	0	0	0	0	0	0	0	0
0	0	0	0	0	0	0	0	0
0	0	0	0	0	0	0	0	0
0	0	0	0	0	0	0	0	0
0	0	0	0	0	0	0	0	0
0	0	0	0	0	0	0	0	0
0	0	0	0	0	0	0	0	0

0	0	0	0	0	0	0	0	0
0	0	0	0	0	0	0	0	0
0	0	0	0	0	0	0	0	0
0	0	0	0	0	0	0	0	0
0	0	0	0	0	0	0	0	0
0	0	0	0	0	0	0	0	0
0	0	0	0	0	0	0	0	0
0	0	0	0	0	0	0	0	0
0	0	0	0	0	0	0	0	0
0	0	0	0	0	0	0	0	0
0	0	0	0	0	0	0	0	0
0	0	0	0	0	0	0	0	0
0	0	0	0	0	0	0	0	0
0	0	0	0	0	0	0	0	0
0	0	0	0	0	0	0	0	0
0	0	0	0	0	0	0	0	0
0	0	0	0	0	0	0	0	0
0	0	0	0	0	0	0	0	0
0	0	0	0	0	0	0	0	0
0	0	0	0	0	0	0	0	0
0	0	0	0	0	0	0	0	0
0	0	0	0	0	0	0	0	0
0	0	0	0	0	0	0	0	0
0	0	0	0	0	0	0	0	0
0	0	0	0	0	0	0	0	0

LC67	HC46	HC47	HC48	HC49	HC50	HC51	HC52	HC53
35679	24905	27455	19694	35163	22342	13239	18629	12001
34	116	76	122	69	51	1316	62	76
1465	40	7603	826	2390	3466	3723	43	11565
9082	7598	50	1281	369	339	440	7273	5553
4210	0	115	0	50	89	123	490	237
0	13	196	10	8396	3243	1412	274	21
1144	640	316	1033	2623	2998	1067	795	44
35	5696	523	444	791	673	953	1634	3481
36	0	784	19	467	96	499	0	1526
434	4678	148	2838	1635	1003	864	202	14
0	83	0	14	0	19	0	6556	97
10	29	88	34	65	56	47	46	69
14	194	11	43	180	71	6	8	31
107	8989	554	1552	148	387	597	3651	354
73	309	0	146	12	19	27	3098	1340
256	243	304	406	822	962	803	89	619
0	40	326	33	0	236	40	0	19
0	15	148	3598	893	461	311	7	0
193	749	433	3274	70	921	145	12	2387
3	0	139	848	540	794	1723	8	0
53	5	0	0	319	381	625	3	0
86	3	62	5	245	493	337	0	0
514	2377	724	7	61	281	47	0	10
15	0	21	0	0	3	0	12	0
0	0	161	391	121	343	334	0	10
0	6	0	8	3	1454	2188	0	8
72	91	54	40	214	168	186	140	156
507	3	0	497	9	398	1320	326	0
482	5	0	101	250	119	534	4	3035
12	9	0	34	404	647	242	0	2576
0	15	123	104	131	67	149	18	26
0	455	114	0	255	231	137	116	0
19	0	99	0	37	112	1264	0	0
0	0	0	0	799	0	163	16	0
35	41	0	0	3	5	0	3	3
0	22	80	124	206	186	234	315	0
0	0	17	0	0	161	628	0	0
0	0	5	0	664	599	456	0	0
29	24	162	276	236	403	188	0	457
70	267	164	251	123	87	210	16	276
0	9	58	0	0	149	88	0	0
0	0	3	0	54	273	641	0	0

0	0	6	0	415	42	220	0	8
0	0	0	0	0	3316	268	0	0
27	0	49	59	132	201	395	0	0
136	0	31	169	239	179	327	0	84
14	0	135	125	23	200	207	0	188
0	51	0	17	162	84	6	24	5
0	0	5	6	8	5	3	0	0
0	0	0	0	0	0	0	0	0
11	0	98	276	0	81	81	3	0
0	6	12	0	784	117	588	48	0
0	46	241	159	21	41	90	51	233
0	13	0	2	9	0	0	0	56
23	4	267	79	32	90	59	73	66
16	38	0	0	23	10	0	163	175
56	0	82	415	28	96	370	0	89
0	0	0	0	63	921	380	0	0
0	0	10	0	46	139	48	0	0
0	135	0	43	40	18	32	87	568
0	204	46	0	57	67	133	617	0
0	0	46	50	57	49	184	0	139
137	18	72	49	0	0	0	77	109
0	0	0	0	244	319	342	0	0
0	0	0	0	0	0	17	29	10
0	0	368	20	18	65	323	0	0
6	0	0	0	0	0	0	0	0
48	0	0	11	0	0	4	23	12
53	687	54	84	0	12	27	67	0
0	0	0	0	35	26	7	11	0
0	0	105	0	10	0	430	0	0
0	0	0	0	0	0	0	0	0
0	0	0	0	23	94	46	0	0
0	0	0	0	0	0	0	0	0
22	3	4	17	0	0	41	25	7
5	0	0	0	0	0	4	5	5
8	0	0	0	0	0	0	0	0
0	0	0	65	0	0	45	0	104
101	92	6	7	25	1656	20	28	6
17	0	17	373	111	79	151	24	0
0	29	0	0	28	19	8	22	0
0	0	0	0	0	0	0	0	5
0	0	44	0	34	31	13	108	0
0	0	0	10	0	7	14	0	0
17	0	0	16	26	12	0	0	17

0	0	0	2253	0	0	0	0	0
0	0	0	0	75	6	0	0	0
0	0	0	0	0	79	198	0	0
0	0	0	0	0	78	6	0	0
0	0	0	0	0	0	0	0	0
0	0	15	0	19	0	0	0	36
0	0	0	0	0	76	0	0	0
0	23	13	18	31	29	12	33	26
0	0	0	0	11	45	86	0	0
0	7	0	0	7	0	0	0	0
0	0	0	0	0	0	0	0	0
9	9	4	11	9	7	12	0	16
0	1753	0	0	0	0	4	38	0
0	0	0	0	51	28	38	0	0
0	0	0	32	16	0	43	0	0
0	0	0	0	5	4	36	0	0
0	0	0	119	0	48	8	56	0
0	0	0	0	0	7	0	0	0
0	15	0	0	9	0	0	0	0
0	0	0	0	0	0	0	0	0
0	0	0	0	0	0	0	2	0
0	0	0	0	0	0	0	0	0
0	0	0	0	2	171	38	0	0
0	0	0	0	11	0	13	0	0
0	0	0	0	0	0	0	0	0
0	0	0	0	0	0	56	0	14
0	8	0	14	9	18	11	17	15
0	0	3	0	2	0	4	0	0
0	0	0	0	0	0	0	0	0
0	4	0	0	0	0	0	0	0
0	0	0	0	0	41	0	0	0
0	0	0	4	0	0	0	0	0
0	0	0	0	0	0	0	0	0
0	0	0	0	9	13	7	7	10
0	0	0	0	0	0	0	0	0
0	0	18	0	0	30	41	0	0
19	0	0	27	0	0	0	7	39
0	0	0	44	0	14	0	0	0
0	0	0	0	0	0	0	0	0
0	0	0	0	0	0	0	0	0
0	0	0	0	0	0	0	0	0
0	0	0	0	15	23	32	0	0
0	0	0	0	0	0	0	19	19

0	0	0	0	0	0	0	0	0
0	0	0	0	12	11	13	0	0
0	0	0	0	0	0	0	0	0
0	6	0	0	0	0	0	0	0
0	0	0	0	26	0	0	0	0
0	0	0	0	0	0	0	0	0
0	0	0	0	24	0	0	0	0
0	0	0	0	0	0	0	0	0
6	5	0	7	0	6	17	0	0
0	0	0	0	0	0	0	0	0
0	0	0	0	0	0	0	0	0
0	0	12	12	0	0	2	22	0
0	0	0	0	0	0	0	0	0
0	0	0	0	0	0	0	0	0
0	0	0	0	0	0	0	0	0
0	0	0	0	0	10	0	0	0
0	0	0	0	0	0	0	0	0
0	0	0	0	0	0	0	0	0
0	0	0	0	0	6	18	0	0
0	0	21	0	8	0	0	0	0
0	0	4	17	7	0	7	0	0
0	0	0	0	0	0	5	0	0
0	0	0	3	11	17	5	5	5
0	0	0	0	0	0	0	0	0
0	0	0	0	0	0	0	0	0
0	0	0	6	0	9	6	0	9
5	0	0	0	0	0	0	0	0
0	0	0	0	0	0	0	0	0
0	0	0	0	0	0	18	0	0
0	0	0	0	0	0	0	0	0
0	0	0	0	0	0	0	0	0
0	0	0	0	0	0	5	0	0
0	0	0	0	0	0	9	0	0
0	0	0	0	0	0	0	0	0
0	0	0	0	30	0	0	0	0
0	0	0	0	0	0	0	0	0
0	0	0	0	0	0	0	0	0
0	0	0	0	6	31	0	0	0
0	0	0	0	0	14	0	0	0
0	0	0	0	0	0	8	0	0
0	0	0	0	0	0	0	0	0
0	0	0	0	0	0	11	0	0
0	0	0	0	68	0	0	0	0

0	0	0	0	0	0	0	0	0
0	0	0	0	0	0	0	0	0
0	0	0	0	0	0	0	0	0
0	0	0	38	0	0	0	0	0
0	0	0	0	0	0	0	0	0
0	0	0	0	0	0	0	0	0
0	0	0	0	0	0	0	0	0
0	0	0	0	0	0	0	0	0
0	0	0	0	0	0	0	0	0
0	0	0	0	0	0	5	0	0
0	0	0	0	0	0	0	0	0
0	0	0	0	0	0	0	0	0
0	0	0	0	0	0	6	22	0
0	0	0	0	0	0	0	0	0
0	0	0	0	0	0	0	0	0
0	0	0	0	0	0	0	0	0
0	0	0	0	0	0	0	0	0
0	0	0	0	0	0	0	0	0
0	0	0	0	0	0	0	0	0
0	0	0	0	0	0	0	0	0
0	0	0	0	0	0	0	0	0
0	0	0	0	0	0	0	0	0
0	0	0	0	0	0	0	0	0
0	0	0	0	0	0	4	0	0
0	0	0	0	0	0	0	0	0
0	6	0	0	0	0	0	0	0
0	0	13	0	0	0	0	0	0
0	0	0	0	0	0	0	0	0
0	0	0	0	0	0	0	0	0
0	0	0	0	0	0	8	0	0
0	0	0	0	0	0	0	0	0
0	0	0	6	0	0	0	0	0
0	0	0	5	0	0	0	0	0
0	0	0	0	0	0	0	0	0
0	9	8	0	0	0	0	0	0
0	0	0	0	0	0	0	9	0
0	0	0	0	0	0	0	0	0
0	0	0	0	0	0	0	0	0
0	0	0	0	0	0	0	0	0
0	0	0	0	0	0	0	0	0
0	0	0	0	0	0	0	0	0
0	0	0	0	0	0	0	0	0
0	0	0	0	0	5	0	0	0

0	5	6	0	0	0	0	0	0
0	0	0	0	0	0	0	0	0
0	0	0	0	0	0	0	0	0
0	0	0	0	3	0	0	0	0
0	0	0	0	0	0	0	0	0
0	0	0	0	0	0	0	0	0
0	6	9	13	0	0	0	0	0
0	0	0	0	0	0	0	0	0
19	0	0	0	0	0	0	0	0
0	0	0	0	0	0	0	0	0
0	0	3	5	0	0	0	0	0
0	0	0	0	0	0	0	0	0
0	0	0	0	0	0	0	0	0
0	0	0	0	0	0	0	0	0
0	0	0	0	0	0	0	0	0
0	0	0	0	0	0	0	0	0
0	6	0	8	0	0	0	0	0
0	0	0	0	0	0	0	0	0
0	0	0	0	0	0	2	0	0
0	0	0	0	0	0	0	0	0
0	0	0	0	7	0	0	0	0
0	0	0	0	0	0	0	0	0
0	0	0	0	0	0	0	0	0
0	0	0	0	0	0	0	0	0
0	0	0	0	0	0	0	0	0
0	0	0	0	0	0	0	0	0
0	0	0	0	0	0	0	0	0
0	0	0	0	0	0	0	0	0
0	0	0	0	0	0	0	0	0
0	0	0	0	0	0	0	0	0
0	0	0	0	0	0	0	0	0
0	0	0	0	0	0	0	0	0
0	0	0	0	0	0	2	0	0
0	0	0	0	0	0	0	0	0
0	0	0	0	7	0	0	0	0
0	0	0	0	0	0	0	0	0
0	0	0	0	0	0	0	0	0
0	0	0	0	0	0	0	0	0
0	0	0	0	0	0	0	0	0
0	0	0	0	0	0	0	0	0
0	0	0	0	0	0	0	0	0
0	0	0	0	0	0	0	0	0
0	0	0	0	0	0	0	0	0

0	0	0	0	0	0	0	0	0
0	0	0	0	6	0	0	0	0
0	0	0	0	0	0	0	0	0
0	0	0	0	0	0	0	0	0
0	0	0	0	0	0	0	0	0
0	10	0	0	0	0	0	0	0
0	0	0	0	0	0	0	0	0
0	0	0	0	0	0	0	0	0
0	0	0	0	0	0	0	0	0
0	0	0	0	0	0	0	0	0
0	0	0	0	0	0	0	0	0
0	0	0	0	0	0	0	0	0
0	0	0	0	0	0	0	0	0
0	0	0	0	0	0	0	0	0
0	0	0	0	0	0	0	0	0
0	0	0	0	0	0	0	0	0
0	0	0	0	0	0	0	0	0
0	0	0	0	0	0	0	0	0
0	0	0	0	0	0	0	0	0
0	0	0	0	0	0	0	0	0
0	0	0	0	0	0	0	0	0
0	0	0	0	0	0	0	4	0
0	0	0	0	0	0	0	0	0
0	0	0	0	0	0	0	0	0
0	0	0	0	0	0	0	0	0
0	0	0	0	0	0	0	0	0
0	0	0	0	0	0	0	0	0
0	0	0	0	0	0	0	0	0
0	0	0	0	0	0	0	0	0
0	0	0	0	0	0	0	0	0
0	0	0	0	0	0	0	0	0
0	0	0	0	0	0	0	0	0
0	0	0	0	0	0	0	0	0
0	0	0	0	0	0	0	0	0
0	0	0	0	0	0	0	0	0
0	0	0	0	0	0	0	0	0
0	0	0	0	0	0	0	0	0
0	0	0	0	0	0	0	0	0
0	0	0	0	0	0	0	0	0
0	0	0	0	0	0	0	0	0
0	0	0	0	0	0	0	0	0
0	0	0	0	0	0	0	0	0
0	0	0	0	0	0	0	0	0

0	0	0	0	0	0	0	0	0
0	0	0	0	0	0	5	0	0
0	0	0	0	0	0	0	0	0
0	0	0	0	0	0	0	0	0
0	0	0	0	0	0	0	0	0
0	0	0	0	0	0	0	0	0
0	0	0	0	0	0	0	0	0
0	0	0	0	0	0	0	0	0
0	0	0	0	0	0	0	0	0
0	0	0	0	0	0	0	0	0
0	0	0	0	0	0	0	0	0
0	0	0	0	0	0	0	0	0
0	0	0	0	0	0	0	0	0
0	0	0	0	0	0	0	0	0
0	0	0	0	0	2	0	0	0
0	0	0	0	0	0	0	0	0
0	0	0	0	0	0	0	0	0
0	0	0	0	0	0	0	0	4
0	0	0	0	0	0	0	0	0
0	0	0	0	0	0	0	0	0
0	0	0	0	0	0	0	0	0
0	0	0	0	0	0	0	0	0
0	0	0	0	0	0	0	0	0
0	0	0	0	0	0	0	0	0
0	0	0	0	0	0	0	0	0
0	0	0	0	0	0	0	0	0
0	0	0	0	0	0	0	0	0
0	0	0	0	0	0	0	0	0
0	0	0	0	0	0	0	0	0
0	0	0	0	0	0	0	0	0
0	0	0	0	0	0	0	0	0
0	0	0	0	0	0	0	0	0
0	0	0	0	0	0	0	0	0
0	0	0	0	0	0	0	0	0
0	0	0	0	0	0	0	0	0
0	0	0	0	0	0	0	0	0
0	0	0	0	0	0	0	0	0
0	0	0	0	0	0	0	0	0
0	0	0	0	0	0	0	0	0
0	0	0	0	0	0	0	0	0
0	0	0	0	0	0	0	0	0
0	0	0	0	0	0	0	0	0
0	3	0	0	0	0	0	0	0

0	0	0	0	0	0	0	0	0
0	0	0	0	0	0	0	0	0
0	0	0	0	0	0	0	0	0
0	0	0	0	0	0	0	0	0
0	0	0	0	0	0	0	0	0
0	0	0	0	0	0	0	0	0
0	0	0	0	0	0	0	0	0
0	0	0	0	0	0	0	0	0
0	0	0	0	0	0	0	0	0
0	0	0	0	0	0	0	0	0
0	0	0	0	0	0	0	0	0
0	0	0	0	0	0	0	0	0
0	0	0	0	0	0	0	0	0
0	0	0	0	0	0	0	0	0
0	0	0	0	0	0	0	0	0
0	0	0	0	0	0	0	0	0
0	0	0	0	0	0	0	0	0
0	0	0	0	0	0	0	0	0
0	0	0	0	0	0	0	0	0
0	0	0	0	0	0	0	0	0
0	0	0	0	0	0	0	0	0
0	0	0	2	0	0	0	0	0
0	0	0	0	0	0	0	0	0
0	0	0	0	0	0	0	0	0
0	0	0	0	0	0	0	0	0

HC54	HC55	LC1	LC2	LC3	LC4	LC5	LC6	LC7
9950	25362	14672	687	3180	27376	34000	39047	3061
13933	44	100	38280	71	42	86	95	19086
2175	5438	38	8540	6764	919	2804	44	673
361	409	323	327	3280	577	237	282	360
49	29	14210	1334	27	315	48	11	35
3453	390	18	56	5	8846	6161	21	202
1218	416	1353	82	19	1437	1656	28	369
683	149	941	163	285	572	429	4271	199
483	366	0	165	12585	125	364	0	72
219	1482	0	174	7747	2404	958	13	12
28	0	8456	0	0	4	0	8	12315
45	54	24	374	10623	36	41	28	60
33	9	147	242	1116	42	342	870	51
279	479	557	335	1475	353	158	0	230
15	34	176	47	731	153	12	16	48
964	436	170	154	1305	660	367	0	216
73	87	27	37	0	164	61	21915	0
779	406	0	417	0	0	701	17	207
283	193	0	274	490	146	127	14	12
578	0	0	0	0	677	440	0	9
421	0	0	0	441	332	3502	8	15
330	86	0	679	0	0	414	9	253
304	0	443	0	250	197	5	8	1431
29	17	21	0	0	0	14	174	0
86	0	0	18	90	124	80	18	927
623	0	0	7	32	8336	1185	11	5
3197	82	107	116	0	718	543	112	454
8	0	0	669	0	0	510	0	0
6	6	8	505	1621	0	61	0	0
609	171	0	0	41	2668	166	2	11
131	192	877	128	205	3506	125	0	399
178	156	0	81	0	205	360	0	32
79	0	0	17	286	0	73	0	60
521	0	0	0	1179	0	79	8	0
0	4	0	0	61	0	58	311	0
121	36	24	11	77	107	110	0	95
141	0	0	212	402	80	361	0	76
246	0	0	0	0	397	36	0	0
135	268	0	14	460	0	491	8	33
85	457	0	17	6	49	170	0	27
700	8	5	9	0	528	434	4	79
0	0	0	0	128	0	50	0	0

21	0	6	0	0	0	41	0	0
299	0	0	0	0	349	1742	0	0
111	80	48	111	1330	164	44	0	66
232	220	0	19	360	1050	69	0	34
7	131	0	63	136	95	1033	0	0
8	8	15	0	5	754	355	10	6
5	0	7	17	0	31	12	14	0
51	370	0	0	0	658	0	0	0
11	0	6	13	0	306	176	3	48
448	0	0	0	0	293	464	0	29
42	0	0	271	298	29	39	0	184
0	27	19	7	215	75	0	5	0
2	0	0	30	2300	223	5	0	10
9	16	0	8	15	11	19	0	22
41	186	0	18	97	0	42	0	0
0	0	0	0	17	0	0	0	0
64	0	0	165	0	96	84	0	58
25	63	0	0	1877	10	0	0	0
54	0	195	0	15	72	22	0	435
64	0	0	0	97	0	58	0	6
12	256	149	22	45	263	8	0	9
127	0	0	37	0	145	53	0	10
0	23	1354	0	43	133	0	9	0
66	25	0	0	0	0	54	3	0
0	6	0	0	0	0	0	0	0
0	0	58	0	90	4	0	86	0
0	26	0	0	0	50	0	0	0
12	0	14	0	0	503	0	0	10
48	0	0	0	0	0	56	0	0
0	0	0	0	0	3	0	0	0
207	38	0	68	0	5	54	0	34
0	0	0	0	0	0	0	0	0
0	0	0	2	28	160	0	0	11
0	7	10	4	17	9	4	28	0
0	0	397	0	0	0	0	0	0
0	0	0	9	0	8	0	0	0
0	43	0	0	12	97	14	0	20
28	16	0	0	9	214	6	0	24
10	0	0	6	0	28	10	0	16
0	0	0	0	478	7	0	0	0
13	0	9	67	0	128	8	0	68
0	0	0	0	0	0	0	0	0
0	13	0	9	41	7	0	8	24

0	0	0	0	0	0	0	0	0
0	0	21	0	0	690	0	0	0
32	0	0	0	0	0	15	0	0
0	0	0	0	0	0	5	0	0
0	0	0	0	0	0	0	0	0
0	41	0	10	0	137	116	0	0
63	0	0	27	0	0	0	0	0
30	25	16	25	38	18	21	18	25
3	0	0	0	0	3	0	0	0
0	0	0	0	0	33	7	0	0
0	0	0	0	7	84	0	0	0
15	0	5	12	8	0	0	16	9
0	0	0	0	0	0	0	0	0
46	0	0	0	0	65	20	0	0
23	51	0	0	0	0	0	0	0
0	0	0	0	0	0	0	0	0
17	53	0	25	0	0	0	0	4
0	0	0	0	0	0	0	0	0
0	0	0	6	26	7	0	0	0
0	0	0	0	0	0	0	0	0
0	0	0	0	147	0	0	0	0
169	0	0	0	0	0	0	0	0
0	0	0	0	0	0	90	0	0
0	0	12	0	0	0	15	0	0
0	0	0	0	0	0	0	0	0
0	0	0	0	46	0	0	0	19
16	0	12	10	7	13	0	15	15
0	0	0	0	0	0	0	0	0
0	0	0	0	0	0	0	0	0
0	0	0	0	0	0	17	0	0
22	0	0	6	0	0	0	0	0
0	37	3	0	0	0	3	0	0
0	0	0	0	0	168	0	0	0
7	7	0	0	0	0	0	0	0
0	0	0	0	0	0	0	0	0
0	0	0	0	0	0	13	0	0
0	196	0	0	0	25	0	0	0
0	0	0	2	0	0	0	0	0
0	0	0	0	0	0	0	0	0
0	0	0	0	0	55	0	0	0
0	0	0	0	0	0	0	0	0
16	0	0	0	0	0	11	0	0
0	0	20	0	0	0	119	0	0

0	0	0	0	0	0	0	0	0
12	0	0	0	0	7	0	0	0
84	0	0	0	0	6	0	0	0
0	0	0	0	7	0	0	0	0
0	0	0	0	0	0	0	0	0
0	0	0	0	0	0	0	0	0
0	0	0	0	0	0	0	0	3
0	0	0	0	0	0	0	0	0
0	0	0	9	0	6	0	0	0
0	13	0	0	0	255	0	0	0
0	0	0	0	0	0	0	0	0
0	0	11	0	0	7	0	0	8
0	0	0	0	113	0	0	0	0
0	0	0	0	6	0	0	0	0
0	0	0	0	0	0	0	0	0
0	0	0	0	0	0	0	0	0
0	0	0	0	0	0	0	0	0
0	0	0	0	0	0	0	20	0
16	0	0	3	0	0	14	0	0
0	0	0	0	3	134	0	0	0
0	0	0	4	0	0	3	0	2
0	0	4	8	0	5	0	6	0
17	4	26	17	0	7	28	9	0
0	0	0	0	0	0	0	0	0
0	0	0	0	0	0	0	0	0
0	0	0	5	0	0	0	4	0
0	0	0	0	0	15	0	0	0
0	0	0	0	0	45	0	0	0
9	0	0	0	0	0	15	0	0
0	0	0	0	0	0	0	0	0
0	16	0	0	0	142	0	0	0
0	0	0	0	0	0	0	0	0
0	0	0	0	0	0	0	0	0
0	0	0	0	0	0	0	0	0
0	0	0	0	0	0	0	0	0
0	0	0	0	0	0	0	0	0
0	0	0	0	0	0	0	0	0
49	0	0	0	0	0	0	0	0
0	99	0	0	0	0	0	0	0
5	0	0	0	0	0	0	0	0
0	0	0	0	0	0	0	0	0
15	0	0	0	0	10	0	0	0
7	0	0	0	0	0	0	0	0

0	0	0	0	0	0	0	0	0
0	0	0	0	0	0	0	0	0
0	0	0	0	12	0	0	0	0
0	0	10	0	0	17	0	0	0
0	0	0	0	8	0	0	0	0
0	0	0	0	0	0	0	0	0
0	0	0	0	0	0	0	0	0
0	0	0	0	0	0	0	0	0
0	0	0	0	0	0	0	0	0
0	0	0	0	0	72	0	0	0
0	0	0	0	0	0	0	0	0
0	0	0	0	0	128	0	0	0
0	0	6	2	0	0	0	0	0
0	0	0	0	0	0	0	0	0
0	0	0	0	0	0	0	0	0
0	0	0	0	0	0	0	0	0
0	0	0	0	0	21	0	0	0
0	0	6	0	3	0	0	0	0
0	0	0	0	0	0	0	0	0
0	0	0	6	0	0	0	0	0
0	0	0	0	0	0	0	0	0
0	0	0	0	0	0	0	0	0
0	0	0	0	0	0	0	0	0
0	0	0	0	0	0	0	0	0
0	0	0	0	0	0	0	0	0
0	0	0	0	0	0	0	0	0
0	0	0	0	0	0	0	0	0
0	0	0	0	0	0	0	0	0
0	0	0	0	0	0	0	0	0
0	0	0	0	0	4	0	0	0
0	0	0	0	0	0	0	0	0
0	0	0	0	0	0	0	0	0
0	0	0	0	0	0	0	5	0
0	0	0	0	0	0	0	0	0
0	0	0	0	0	0	0	0	0
0	0	0	0	0	0	0	0	0
0	0	0	0	0	0	0	0	0
0	0	0	0	0	0	0	0	0
0	0	0	0	0	0	0	0	0
0	0	0	0	0	0	0	0	0
0	0	0	0	0	12	0	0	0
0	0	0	0	0	1	0	0	0
0	0	0	0	0	0	0	0	0

0	0	0	0	0	0	0	0	0
0	0	0	0	0	24	0	0	0
0	0	0	0	0	0	0	0	0
0	0	0	0	0	15	0	0	0
0	0	0	0	0	0	0	0	0
0	0	0	0	0	0	0	0	0
0	0	0	0	0	0	0	0	0
0	0	0	0	0	0	0	0	0
0	0	0	0	0	3	0	0	0
0	0	0	0	0	38	0	0	0
0	0	0	0	0	0	0	0	0
0	0	0	0	0	0	0	0	0
0	0	0	0	0	0	0	0	0
0	0	0	0	0	0	0	0	0
0	0	0	0	0	0	0	0	0
0	0	0	0	0	0	0	0	0
0	0	0	0	0	0	0	0	0
0	0	0	0	5	0	0	5	0
0	0	0	0	0	0	0	0	0
0	0	0	0	0	26	0	0	0
4	0	5	0	0	0	0	0	5
0	0	0	0	0	0	0	0	0
0	0	0	0	0	0	0	0	0
0	0	0	0	0	0	0	0	0
0	0	0	0	0	0	0	0	0
0	0	0	0	0	0	0	0	0
0	0	0	0	0	0	0	0	0
0	0	0	0	0	0	0	0	0
0	0	0	4	0	0	0	0	0
0	0	0	0	0	0	0	0	0
0	0	0	0	0	0	0	0	0
0	0	0	0	0	0	0	0	0
0	0	0	0	0	0	0	0	0
0	0	0	0	0	0	5	0	0
0	0	0	0	0	0	0	0	0
0	0	0	0	0	0	0	0	0
3	0	0	0	0	0	0	0	4
0	0	0	0	0	0	0	0	0
0	0	0	0	0	0	0	0	0
0	0	0	0	0	0	0	0	0
0	0	0	0	0	0	0	0	0
0	0	0	0	0	0	0	0	0
0	0	0	0	0	0	0	0	0

0	0	0	0	0	0	0	0	0
0	0	0	0	0	0	0	0	0
0	0	0	0	0	0	0	0	0
0	0	0	0	0	0	0	0	0
0	0	0	0	0	0	0	0	0
0	0	0	0	0	0	0	0	0
0	0	0	0	0	0	0	0	0
0	0	0	0	0	0	0	0	0
0	0	0	0	0	0	0	0	0
0	0	0	0	0	0	0	0	0
0	0	0	0	0	0	0	0	0
0	0	0	0	0	0	0	0	0
0	0	0	0	0	0	0	8	0
0	0	0	0	0	0	0	0	0
0	0	0	0	0	0	0	0	0
0	0	0	0	0	0	0	0	6
0	0	0	0	0	0	0	0	0
0	0	0	0	0	0	0	0	0
0	0	0	0	0	0	0	0	0
0	0	0	0	0	0	0	0	0
0	0	0	0	0	0	0	0	0
0	0	0	0	0	0	0	0	0
0	0	0	0	0	0	0	0	0
0	0	0	0	0	0	0	0	0
0	0	0	0	0	0	0	0	0
0	0	0	0	0	0	0	0	0
0	0	0	0	0	0	0	0	0
0	0	0	0	0	0	0	0	0
0	6	0	0	0	0	0	0	0
0	0	0	0	0	0	0	0	0
0	0	0	0	0	0	0	0	0
0	0	0	0	0	0	0	0	0
0	0	0	0	0	0	0	0	0
0	0	0	0	0	0	0	0	0
0	0	0	0	0	0	0	0	0
0	0	0	0	0	0	0	0	0
0	0	0	0	0	0	0	0	0
0	0	0	0	0	0	0	0	0
0	0	0	0	0	0	0	0	0
0	0	0	0	0	0	0	0	0
0	0	0	0	0	0	0	0	0
0	0	0	0	0	0	0	0	0
0	0	0	0	0	0	0	0	0

0	0	0	0	0	0	0	0	0
0	0	0	0	0	0	0	0	0
0	0	0	0	0	0	0	0	0
0	0	0	0	0	0	0	0	0
0	0	0	0	0	0	0	0	0
0	0	0	0	0	0	0	0	0
0	0	0	0	0	0	0	0	0
0	0	0	0	0	0	0	0	0
0	0	0	0	0	0	0	0	0
0	0	0	0	0	0	0	0	0
0	0	0	0	0	0	0	0	0
0	0	0	0	0	0	0	0	0
0	0	0	0	0	0	0	0	0
0	0	0	0	0	0	0	0	0
0	0	0	0	0	0	0	0	0
0	0	0	0	0	0	0	0	0
0	0	0	0	0	0	0	0	0
0	0	0	0	0	0	0	0	0
0	0	0	0	0	0	0	0	0
0	0	0	0	0	0	0	0	0
0	0	0	0	0	0	0	0	0
0	0	0	0	0	0	0	0	0
0	0	0	0	0	0	0	0	0
0	0	0	0	0	0	0	0	0
0	0	0	0	0	0	0	3	0
0	0	0	0	0	0	0	0	0
0	0	0	0	0	0	0	0	0
0	0	0	0	0	0	0	0	0
0	0	0	0	0	0	0	0	0
0	0	0	0	0	0	0	0	0
0	0	0	0	0	0	0	0	0
0	0	0	0	0	0	0	0	0
0	0	0	0	0	0	0	0	0
0	0	0	0	0	0	0	0	0
0	0	0	0	0	0	0	0	0
0	0	0	0	0	0	0	0	0
0	0	3	0	0	0	0	0	0
0	0	0	0	0	0	0	0	0
0	0	0	0	0	0	0	0	0
0	0	0	0	0	0	0	0	0
0	0	0	0	0	0	0	0	0
0	0	0	0	0	0	0	0	0
0	0	0	0	0	0	0	0	0

0	0	0	0	0	0	0	0	0
2	0	0	0	0	0	0	0	0
0	0	0	0	0	0	0	0	0
0	0	0	0	0	0	0	0	0
0	0	0	0	0	0	0	0	0
0	0	0	0	0	0	0	0	0
0	0	0	0	0	0	0	0	0
0	0	0	0	0	0	0	0	0
0	0	0	0	0	0	0	0	0
0	0	0	0	0	0	0	0	0
0	0	0	0	0	0	0	0	0
0	0	0	0	0	0	0	0	0
0	0	0	0	0	0	0	0	0
0	0	0	0	0	0	0	0	0
0	0	0	0	0	0	0	0	0
0	0	0	0	0	0	0	0	0
0	0	0	0	0	0	0	0	0
0	0	0	0	0	0	0	0	0
0	0	0	0	0	0	0	0	0
0	0	0	0	0	0	0	0	0
0	0	0	0	0	0	0	0	0
0	0	0	0	0	0	0	0	0
0	0	0	0	0	0	0	0	0
0	0	0	0	0	0	0	0	0
0	0	0	0	0	0	0	0	0

LC8	LC10	LC11	LC12	LC16	LC17	LC18	LC19	LC20
5706	24822	18725	23520	34376	11513	23890	43076	41188
28233	134	104	50	51	376	58	53	65
1581	76	1428	4883	463	1663	1676	1226	37
1594	16713	121	97	392	38748	215	5947	2263
0	0	39	7965	297	333	184	179	2727
5	75	255	14	7	18	15	10	13
554	382	217	5	16	5	160	965	10
203	28	547	804	1335	194	374	23	1044
56	0	40	2623	1073	26	0	0	16
1425	0	24	26	12	44	11	1248	28
14	13	12810	23	8474	46	940	194	1172
33	20	373	100	63	68	6792	74	1478
33	10	22	9	31	89	428	21	905
170	479	843	251	374	0	286	55	131
450	164	153	9	22	108	710	21	1269
127	93	155	1232	507	190	1195	1440	8
0	0	19	0	1178	209	11	32	606
39	0	56	0	740	23	0	19	0
373	9	1563	481	1824	2813	379	15	13
121	0	0	138	0	7	0	0	0
0	0	1	114	0	10	3	0	0
172	0	12	245	0	7	5	0	0
679	0	1019	7	11	8	860	18	22
0	20	0	0	0	0	23	25	9
43	17	302	75	554	462	0	29	0
0	0	0	0	0	0	0	0	0
78	76	78	70	73	55	83	92	63
0	141	0	630	0	0	3	1973	2377
21	20	7	224	0	9	5	244	8
0	0	11	106	0	13	0	0	0
161	0	74	0	253	6	10	0	0
202	0	52	129	19	0	0	0	0
154	0	1580	1868	2683	0	0	0	0
5	0	0	595	0	0	0	0	0
254	3	0	5	3	0	0	5	8
40	19	10	58	9	0	61	23	0
96	0	7	0	0	0	0	4	0
5	0	0	0	0	0	0	0	0
79	0	43	418	80	47	91	0	0
116	0	37	103	164	0	57	254	4
7	0	61	0	0	0	22	0	0
0	2	0	0	0	0	0	0	0

0	7	0	0	0	0	8	8	0
0	0	94	0	0	0	0	0	0
70	0	45	145	16	0	0	0	0
52	2	30	236	29	0	6	1021	0
321	0	109	0	563	0	0	0	0
0	7	3	5	44	7	8	108	0
0	0	0	0	0	0	3	0	0
0	0	0	0	0	0	0	0	0
17	0	91	376	130	0	0	0	0
0	0	0	0	0	0	0	0	0
117	0	121	134	441	0	0	0	0
0	0	0	7	4	27	29	25	34
78	11	52	79	36	29	21	0	0
10	14	255	33	5	7	5	4	4
15	0	15	259	0	0	7	0	0
0	0	0	0	0	0	0	0	0
72	0	45	81	11	0	0	0	6
8	0	31	0	5	0	0	0	0
6	0	75	0	0	2	0	0	0
21	0	9	1425	44	0	0	0	0
0	17	0	22	0	82	43	0	44
0	0	0	0	0	0	0	0	0
14	0	52	19	0	0	36	0	0
0	0	0	0	0	0	0	0	0
0	0	0	0	0	0	0	0	0
48	0	0	4	0	11	20	6	65
12	0	4	0	5	0	28	0	0
0	0	0	0	0	0	0	0	0
24	0	45	424	0	0	0	0	0
0	0	0	0	0	0	0	0	0
80	0	0	0	0	0	0	0	0
0	0	0	0	0	0	0	0	0
0	5	8	45	0	0	0	5	0
0	0	3	0	0	0	10	0	0
0	0	0	0	0	0	24	3254	79
0	0	0	0	26	85	37	29	2734
18	0	0	5	13	0	14	10	0
4	0	0	32	0	0	0	0	0
23	25	0	11	0	0	13	12	0
0	0	16	0	0	0	0	0	0
29	0	84	39	0	179	0	0	0
0	0	0	61	0	8	25	0	169
13	11	18	5	18	33	8	20	23

0	0	0	0	0	0	0	0	0
5	0	0	37	0	0	0	11	0
0	0	0	0	0	0	0	0	0
0	0	0	0	0	86	0	0	0
0	0	0	0	0	0	0	0	0
11	0	0	4	0	0	0	0	0
19	0	0	0	0	0	0	0	0
30	24	20	28	33	17	46	15	27
0	0	0	0	0	0	0	0	0
0	0	0	0	0	0	0	6	0
0	0	0	0	0	0	0	4	0
12	9	14	0	6	11	10	9	13
0	0	0	0	0	0	0	0	0
11	0	0	0	0	0	0	0	0
0	0	0	208	0	0	0	0	0
0	0	0	0	0	0	0	0	0
0	0	0	0	0	0	0	0	0
0	0	0	0	0	0	0	0	0
0	0	0	0	5	0	0	168	14
0	0	0	105	0	0	0	0	0
0	0	0	0	0	0	0	0	28
141	0	0	0	0	0	0	0	0
0	0	0	0	0	0	0	0	0
0	0	0	0	0	0	0	0	0
0	0	0	0	4	9	0	0	0
0	0	17	0	0	0	0	0	0
22	16	18	0	11	12	9	16	18
0	0	0	0	0	0	0	0	0
0	0	4	0	0	0	4	0	0
0	0	0	0	0	0	0	0	0
11	0	0	0	0	0	0	0	0
0	0	0	34	7	14	7	0	252
0	11	0	0	0	0	0	0	0
0	0	0	0	0	0	0	0	0
0	0	0	0	0	0	0	0	0
0	0	31	57	0	0	0	0	0
0	0	0	0	0	0	0	0	0
0	0	0	0	0	0	0	4	9
0	0	0	0	0	0	0	0	0
0	0	0	0	0	0	0	0	0
0	0	0	0	0	0	0	0	0
0	0	0	0	0	0	0	0	0
0	13	0	0	0	0	17	0	0

0	0	0	0	0	0	0	0	0
0	0	0	0	0	0	0	0	0
0	0	0	27	0	0	0	0	0
0	0	0	0	0	0	0	3	16
0	0	0	0	0	0	0	0	0
0	0	0	0	0	0	0	0	0
0	0	0	0	0	0	0	0	0
0	0	0	0	0	0	0	0	0
12	0	0	0	0	9	0	0	0
0	0	0	0	0	0	0	0	0
0	0	0	0	0	0	0	0	0
0	5	0	0	0	0	0	13	0
7	0	0	0	0	0	0	0	0
0	0	0	0	0	0	0	8	6
0	0	0	0	0	0	0	0	0
0	0	0	0	0	0	0	0	0
0	0	0	0	0	0	0	0	0
0	0	0	0	0	0	0	0	0
0	0	0	0	0	0	0	0	0
0	0	0	0	0	0	0	0	0
0	0	0	0	0	0	5	4	0
0	0	0	0	0	0	0	0	0
0	0	0	0	0	0	0	0	0
0	0	0	0	0	0	7	8	0
0	0	0	0	0	0	0	0	0
0	0	7	0	0	0	0	0	5
0	0	0	0	0	0	0	0	0
0	0	0	0	0	0	0	0	0
0	0	0	0	0	0	0	0	0
0	0	0	8	0	3	0	0	0
0	0	0	0	0	0	0	0	0
0	0	0	0	0	5	12	0	50
0	0	0	0	0	0	0	0	0
0	0	0	0	0	0	0	0	0
0	0	0	0	0	0	0	0	0
0	0	0	0	21	25	28	16	32
0	0	0	0	0	0	0	0	0
0	0	0	0	0	0	0	0	0
0	0	0	0	0	0	0	0	0
0	0	0	0	0	0	0	0	0
0	0	0	0	0	0	0	0	0
0	0	0	0	0	0	0	0	0
0	0	0	0	0	0	0	0	0

0	0	0	0	0	0	0	0	0
0	0	0	0	0	0	0	0	0
0	0	0	0	0	0	0	0	0
0	0	0	7	0	0	0	0	0
0	0	0	0	0	0	0	0	0
0	0	0	0	0	0	0	0	0
0	0	0	0	0	0	0	0	0
0	0	0	0	0	0	0	0	0
0	0	0	0	0	0	0	0	0
0	0	0	0	0	0	0	0	0
0	0	0	0	0	0	0	0	0
0	0	0	0	0	0	0	0	0
0	0	0	0	0	0	0	0	0
0	0	0	35	0	0	0	0	77
0	0	0	0	0	0	0	0	0
0	0	0	0	0	0	0	0	0
0	0	0	0	0	0	0	0	0
0	0	0	6	5	0	0	0	0
0	0	0	0	0	0	0	0	0
0	0	0	0	0	0	0	0	0
0	0	0	0	0	0	0	0	0
0	0	0	0	0	0	0	0	0
0	0	0	0	0	0	0	0	0
0	0	0	0	0	0	0	0	0
0	0	0	0	0	0	0	0	0
0	0	0	0	0	0	0	0	0
0	0	0	0	0	0	0	0	0
0	0	0	0	0	0	0	0	0
0	0	0	0	0	0	0	0	0
0	0	0	0	0	0	0	0	0
0	0	0	0	0	0	25	0	0
0	0	0	0	0	0	0	0	0
0	0	0	0	0	0	0	0	0
0	0	0	0	0	0	0	0	0
0	0	0	0	0	0	0	0	0
0	0	0	0	0	0	0	0	0
0	0	0	0	0	0	0	0	0
0	0	0	0	0	0	0	0	0
0	0	0	0	0	0	9	0	0
0	0	0	0	0	0	0	0	0
0	0	0	0	0	0	0	0	0
0	0	0	0	0	0	0	0	0
0	0	0	0	0	0	0	0	0

0	0	0	0	0	0	0	0	0
0	0	0	0	0	0	0	0	0
0	0	0	0	0	0	0	0	0
0	0	0	0	0	0	0	0	0
0	0	0	0	0	0	0	0	0
0	0	0	0	0	0	0	0	0
0	0	0	0	0	0	0	0	0
0	0	0	0	0	0	0	0	0
0	0	0	0	0	0	0	0	0
0	0	0	0	0	0	0	0	0
0	0	0	0	0	0	0	0	0
0	0	0	0	0	0	0	0	0
7	0	0	4	0	0	0	0	0
0	0	0	0	0	0	0	0	0
0	0	0	0	0	0	0	0	0
0	0	0	0	0	0	0	0	0
0	0	0	0	0	0	0	0	0
0	0	0	0	0	2	0	0	0
0	2	0	0	6	0	4	0	0
0	0	0	0	0	0	0	0	0
0	0	0	0	0	0	0	0	0
0	0	0	0	0	0	0	0	0
0	0	0	0	0	0	0	0	0
0	0	0	0	0	0	0	0	0
0	0	0	0	0	0	0	0	0
0	0	0	0	0	0	0	0	0
0	0	0	0	0	0	0	0	0
0	0	0	0	0	0	0	0	0
0	0	0	0	0	0	0	0	0
0	0	0	0	0	0	0	0	0
4	0	0	0	0	0	0	0	0
0	0	0	0	0	0	6	0	0
0	0	0	0	0	0	0	0	0
0	0	0	0	3	0	0	0	0
0	0	0	0	0	0	0	0	0
15	0	0	0	0	0	0	0	0
0	0	0	0	0	0	0	0	0
0	0	0	0	0	0	0	6	7
0	0	0	0	0	0	0	0	0
0	0	0	0	0	0	0	0	0
0	0	0	0	0	0	0	0	0
0	0	0	0	0	0	0	0	0
0	0	0	0	0	0	0	0	0

0	0	0	0	0	0	0	0	3
0	0	0	0	0	0	0	0	0
0	0	0	0	0	0	0	0	0
0	0	0	0	0	0	0	0	0
0	0	0	0	0	0	0	0	0
0	0	0	0	0	0	0	0	0
0	0	0	0	0	0	10	0	0
0	0	0	0	0	0	0	0	0
0	0	0	0	0	0	0	0	0
0	0	0	0	0	0	0	0	0
0	0	0	0	0	0	0	0	0
0	0	0	0	0	0	0	0	0
0	0	0	0	0	0	0	0	0
0	0	0	0	0	0	8	0	0
0	0	0	0	0	0	0	0	0
0	0	0	0	0	0	0	0	0
0	0	0	0	0	0	0	0	0
0	0	0	0	0	0	0	0	0
0	0	0	0	0	0	0	0	0
0	0	0	0	0	0	0	0	0
0	0	0	0	0	0	0	0	0
0	0	0	0	0	0	0	0	0
0	0	0	0	0	0	0	0	0
0	0	0	0	0	0	0	0	0
0	0	0	0	0	0	0	0	0
0	0	0	0	0	0	0	0	0
0	0	0	0	0	0	0	0	0
0	0	0	0	0	0	0	0	0
0	0	0	0	0	0	0	0	0
0	0	0	0	0	0	0	0	0
0	0	0	0	0	0	0	0	0
0	0	0	0	0	0	6	0	0
0	0	0	0	0	0	0	0	0
0	0	0	0	0	0	0	0	0
0	0	0	0	0	0	0	0	0
0	0	0	0	0	0	0	0	0
0	0	0	0	0	0	0	0	0
0	0	0	0	0	0	0	0	0
0	0	0	0	0	0	0	0	0
0	0	0	0	0	0	0	0	0
0	0	0	0	0	0	0	0	0
0	0	0	0	0	0	0	0	0
0	0	0	0	0	0	0	0	0

0	0	0	0	0	0	0	0	0
0	0	0	0	0	0	0	0	0
0	0	0	0	0	0	0	0	0
0	0	0	0	0	0	0	0	0
4	0	0	0	0	0	0	0	0
0	0	0	0	0	0	0	0	0
0	0	0	0	0	0	0	0	0
4	0	0	0	0	0	0	0	0
0	0	0	0	0	0	0	0	0
0	0	0	0	0	0	0	0	0
0	0	0	0	0	0	0	0	0
0	0	0	0	0	0	0	0	0
0	0	0	0	0	0	0	0	0
0	0	0	0	0	0	0	0	0
0	0	0	0	0	0	0	0	0
0	0	0	0	0	0	0	0	0
0	0	0	0	0	0	0	0	0
0	0	0	0	0	0	0	0	0
0	0	0	0	0	0	0	0	0
0	0	0	0	0	0	0	0	0
0	0	0	0	0	0	0	0	0
0	0	0	0	0	0	0	0	0
0	0	0	0	0	0	0	0	0
0	0	0	0	0	0	0	0	0
0	0	0	0	0	0	0	0	0
0	0	0	0	0	0	0	0	0
0	0	0	0	0	0	0	0	0
0	0	0	0	0	0	0	0	0
0	0	0	0	0	0	0	0	0
0	0	0	0	0	0	0	0	0
0	0	0	0	0	0	0	0	0
0	0	0	0	0	0	0	3	0
0	0	0	0	0	0	0	0	0
0	0	0	0	0	0	0	0	0
0	0	0	0	0	0	0	3	0
0	0	0	0	0	0	0	0	0
0	0	0	0	0	0	0	0	0
0	0	0	0	0	0	0	0	0
0	0	0	0	0	0	0	0	0
0	0	0	0	0	0	0	0	0
0	0	0	0	0	0	0	0	0
0	0	0	0	0	0	0	0	0
0	0	0	0	0	0	0	0	0

0	0	0	0	0	0	0	0	0
0	0	0	0	0	0	0	0	0
0	0	0	0	0	0	0	0	0
0	0	0	0	0	0	0	0	0
0	0	0	0	0	0	0	0	0
0	0	0	0	0	0	0	0	0
0	0	0	0	0	0	0	0	0
0	0	0	0	0	0	0	0	0
0	0	0	0	0	0	0	0	0
0	0	0	0	0	0	0	0	0
0	0	0	0	0	0	0	0	0
0	0	0	0	0	0	0	0	0
0	0	0	0	0	0	0	0	0
0	0	0	0	0	0	0	0	0
0	0	0	0	0	0	0	0	0
0	0	0	0	0	0	0	0	0
0	0	0	0	0	0	0	0	0
0	0	0	0	0	0	0	0	0
0	0	0	0	0	0	0	0	0
0	0	0	0	0	0	0	0	0
0	0	0	0	0	0	0	0	0
0	0	0	0	0	0	0	0	0
0	0	0	0	0	0	0	0	0
0	0	0	0	0	0	0	0	0
0	0	0	0	0	0	0	0	0

LC21	LC22	LC23	LC25	LC35	LC43	LC45	LC62	LC64
13229	29737	22268	38405	16021	3331	29354	22064	32480
3879	42	108	68	109	41	18	37	105
1675	2546	4467	2201	5424	47	1824	3120	1578
10954	340	4809	125	358	3142	359	10753	11049
575	1052	4489	104	111	10	2297	276	756
2708	21	849	17	17	10981	1059	113	0
257	265	3003	2490	91	672	945	190	30
316	2061	861	352	10314	1283	413	1340	3480
642	1694	2617	101	0	83	198	1360	19
52	678	27	91	230	139	39	59	42
16	20	19	6	37	14	0	29	0
88	9637	73	30	44	13	25	46	19
14	2895	51	597	40	2364	38	0	90
158	1365	838	447	762	1939	129	333	0
0	41	48	12	41	0	0	210	695
233	655	514	463	717	4177	880	268	1284
32	10	5	0	31	13	59	129	5
27	36	937	509	0	100	591	27	0
1133	77	2511	175	202	0	36	942	73
436	12	386	5057	0	351	61	6	0
228	349	885	1998	239	1595	366	0	3
465	770	55	1176	251	6522	285	37	7
115	4273	13	5	10	83	0	899	0
18	0	0	47	0	0	23	59	36
83	21	133	148	25	10	379	37	0
0	0	0	0	0	70	0	0	0
56	139	66	213	84	35	0	89	85
0	4	290	404	2574	0	117	0	261
2153	11	7	745	167	0	350	13	547
594	0	528	660	0	0	236	0	0
74	559	125	1316	150	16	318	510	0
62	299	0	2	61	124	0	4	0
803	12	645	112	0	0	31	0	3
0	1154	7	0	0	0	60	708	5
4	9	0	56	0	5	3	0	0
76	101	139	429	96	1146	77	135	0
103	178	39	145	0	0	318	37	6
8	0	17	676	0	0	26	0	0
127	64	152	162	693	0	82	66	0
35	192	324	27	578	142	65	56	1753
0	0	2	0	0	26	0	9	0
10	142	76	108	0	0	8	0	8

0	0	7	432	2	208	0	0	0
0	0	0	0	0	393	0	0	0
91	120	51	137	78	34	96	259	0
81	353	117	94	85	344	132	30	278
22	17	57	91	79	0	43	21	10
6	413	17	71	5	47	0	0	5
0	0	0	0	0	0	0	0	0
0	0	0	0	0	0	0	13	5
45	65	145	423	0	150	22	89	0
145	11	125	0	0	439	0	0	0
40	416	40	37	756	0	19	279	0
0	5	0	0	22	5559	0	0	225
10	70	115	31	98	353	21	75	0
22	19	0	0	0	104	5	850	70
269	6	35	56	32	0	7	256	4
0	0	4	501	0	0	0	0	0
124	1139	234	87	0	8	0	0	88
0	103	0	0	0	0	11	96	0
9	1458	73	0	0	0	0	136	0
79	11	82	34	127	0	8	0	90
0	14	6	18	378	0	24	92	344
1008	13	86	0	0	0	0	0	0
8	13	0	12	72	102	0	0	0
25	0	599	257	40	0	0	47	0
14	0	0	0	0	0	0	42	5
0	5	4	0	6	2483	0	18	12
8	5	14	30	12	0	0	146	0
412	0	11	24	0	1893	81	17	0
0	118	158	11	120	0	0	71	0
0	0	0	0	0	0	0	0	0
24	0	120	157	0	0	29	0	0
0	3	0	0	0	0	0	0	0
0	14	46	59	50	3905	27	0	0
9	34	3	6	0	23	0	0	0
6	0	0	0	0	0	0	0	0
40	27	0	0	14	0	0	786	0
4	10	0	105	0	27	10	38	0
0	0	4	88	19	668	37	0	0
0	41	12	32	0	18	0	0	0
0	8	0	0	0	0	0	22	0
23	0	7	16	0	27	0	22	0
0	34	0	37	15	0	0	0	0
0	37	22	83	0	161	0	0	15

0	0	0	0	0	0	0	0	0
0	0	0	0	0	0	0	0	0
0	0	0	237	0	0	97	0	0
0	0	0	497	0	0	14	0	0
0	9	0	0	0	0	0	0	0
0	0	0	114	33	140	85	53	0
29	0	0	96	0	0	0	0	0
17	21	19	14	25	4	0	8	8
0	0	0	0	0	0	0	0	0
0	16	0	6	0	12	0	0	0
0	0	0	0	0	2250	6	0	0
0	0	11	7	0	0	0	0	7
0	0	0	0	0	0	0	0	0
0	0	0	52	0	436	51	0	0
0	0	0	0	0	31	0	0	0
0	0	0	0	0	0	0	0	0
0	0	6	0	84	0	0	0	0
0	0	0	0	0	0	0	0	0
0	41	0	46	0	73	0	0	0
0	52	0	0	0	0	0	0	0
0	0	0	0	0	0	0	0	0
0	0	0	0	0	0	0	0	0
0	0	0	0	0	0	0	0	0
0	0	0	13	0	1105	0	0	0
0	0	0	0	0	0	0	0	0
0	0	0	16	0	0	31	0	0
15	30	11	11	2	8	6	0	0
0	0	0	6	0	0	0	0	0
0	0	0	0	0	0	0	0	0
0	8	0	0	0	0	0	0	0
0	0	0	0	0	956	0	0	0
4	0	0	7	3	5	12	0	0
0	0	0	49	0	0	0	22	0
0	0	0	0	0	0	0	0	0
0	0	0	0	0	0	0	0	0
10	0	24	6	0	0	0	0	0
0	0	0	0	0	47	0	0	0
0	0	0	0	2	0	0	0	0
0	0	0	0	0	805	0	0	0
0	0	0	0	0	219	12	0	0
0	0	0	0	0	0	0	0	0
11	0	0	12	0	0	8	7	0
0	0	0	0	0	0	0	5	0

0	0	0	0	0	0	0	0	0
0	0	0	24	0	0	0	0	0
0	0	0	0	0	84	0	0	0
0	46	0	0	0	260	0	0	0
0	0	0	0	0	0	0	0	0
0	0	0	0	0	0	0	0	0
0	0	0	0	0	282	0	0	0
0	0	0	0	0	0	0	0	0
0	10	0	0	0	3	0	0	0
0	0	0	5	0	182	0	0	0
30	0	0	0	0	5	0	0	0
0	19	4	0	0	0	0	14	0
6	0	0	0	0	147	0	0	0
0	0	0	7	0	12	0	0	0
0	0	0	0	0	0	0	0	0
6	0	0	0	0	91	0	0	0
0	0	0	0	0	231	0	0	0
0	0	0	0	0	0	0	0	0
0	0	17	3	0	0	0	0	0
0	2	3	15	0	0	0	0	0
0	0	7	0	0	0	0	0	2
0	0	0	0	0	0	0	0	0
0	0	0	0	0	0	0	0	0
0	0	0	0	0	0	0	0	0
0	0	0	0	0	0	0	0	0
3	3	0	0	5	0	0	0	0
0	0	0	6	0	148	0	0	0
0	0	0	4	5	95	0	0	0
0	0	0	0	0	0	0	0	0
0	0	0	0	0	0	0	0	0
0	0	0	10	0	0	0	0	0
0	0	0	0	0	0	0	0	0
0	0	0	15	0	36	0	0	0
0	0	0	0	0	96	0	0	0
0	0	0	0	0	0	0	0	0
25	21	7	12	0	0	0	0	0
0	0	0	0	0	0	0	0	0
0	0	0	0	0	13	0	0	0
0	0	0	0	0	0	0	0	0
0	0	0	0	0	132	0	0	0
0	0	0	0	0	0	0	0	0
0	0	0	0	0	112	0	0	0
0	0	0	0	0	92	0	0	0

0	12	0	0	0	0	0	0	0
0	0	0	0	0	0	0	0	0
0	0	0	0	0	80	0	0	0
0	0	0	0	0	9	0	0	0
0	0	0	4	0	46	0	0	0
0	0	0	0	0	0	0	76	0
0	6	0	0	0	0	0	0	0
0	24	0	0	0	0	0	0	0
0	0	0	0	0	0	0	0	0
0	0	0	0	0	0	0	0	0
0	0	0	0	0	0	0	0	0
0	0	0	0	0	0	0	0	0
0	0	0	0	0	0	0	0	0
0	0	0	0	0	0	0	0	0
0	0	0	0	0	0	0	0	0
0	0	0	0	0	0	0	0	0
0	0	0	0	0	0	0	0	0
0	0	0	0	0	0	0	0	0
0	0	0	0	0	0	0	0	0
0	14	0	0	0	3	0	0	0
0	0	0	0	0	0	0	0	0
0	0	0	0	0	72	0	0	0
0	0	0	0	0	0	0	0	0
0	0	0	0	0	60	0	0	0
0	0	0	0	0	0	0	0	0
0	0	0	5	0	5	0	0	0
0	0	0	0	0	0	0	0	0
0	2	0	0	0	26	0	0	0
0	0	0	0	0	0	0	16	0
2	5	0	4	0	0	0	0	0
0	0	0	0	0	0	0	0	0
0	0	0	0	3	0	0	0	0
0	0	0	0	0	0	0	0	0
0	0	0	0	0	0	0	0	0
0	0	0	0	0	0	0	0	0
0	0	0	0	0	0	0	0	44
0	0	0	0	0	52	0	0	0
0	0	0	0	0	0	0	0	0
0	0	0	0	0	0	0	0	0
0	0	0	0	0	0	0	0	0
0	0	0	0	0	0	0	0	0
0	0	0	1	0	0	0	0	0
0	0	0	0	0	0	0	0	0

0	0	0	0	0	0	0	0	0
0	0	0	0	0	0	0	0	0
0	0	0	0	0	4	0	0	0
0	0	0	0	0	0	0	0	0
0	0	0	8	0	0	0	0	0
0	0	0	0	0	0	0	0	0
0	0	0	0	0	0	0	0	0
0	0	0	5	0	0	0	0	0
0	0	0	0	0	0	0	0	0
0	0	0	0	0	0	0	0	0
0	0	0	0	0	0	0	0	0
0	0	0	0	0	0	0	0	0
0	0	0	0	0	0	0	0	0
0	0	0	0	0	0	0	0	0
0	0	0	0	0	0	0	0	0
0	0	0	0	0	0	0	0	0
0	0	0	0	0	0	0	0	0
0	0	0	0	0	0	0	0	0
0	0	0	0	0	0	0	0	0
0	0	0	0	0	0	0	0	0
0	0	0	0	0	0	0	0	0
0	0	0	0	0	0	0	0	0
0	0	0	0	0	0	0	0	0
0	8	0	0	0	0	0	0	0
0	0	0	0	0	0	0	0	0
0	0	0	0	0	0	0	0	0
0	0	0	0	0	0	0	0	0
0	0	0	0	0	0	0	0	0
0	0	0	0	0	0	0	0	0
0	0	0	0	0	10	0	0	0
0	0	0	0	0	0	0	0	0
7	5	0	0	0	0	0	0	0
0	0	0	0	0	0	0	0	0
0	0	0	2	0	0	0	0	0
0	0	0	0	0	0	0	0	0
0	0	0	0	0	0	0	0	0
0	0	0	0	0	0	0	0	0
0	0	0	0	0	0	0	0	0
0	0	0	0	0	0	0	0	0
0	0	0	0	0	0	0	0	0
0	0	0	0	0	0	0	0	0
0	0	0	0	0	0	0	0	0
0	0	0	0	0	0	0	0	0

3	0	0	0	0	0	0	0	0
0	0	0	0	0	0	0	0	0
0	0	0	0	0	0	0	0	0
0	0	0	0	0	0	0	0	0
0	0	0	0	0	0	0	0	0
0	0	0	0	0	0	0	0	0
0	0	0	0	0	0	0	0	0
0	0	0	0	0	0	0	0	0
2	0	0	0	0	0	0	0	0
0	0	0	0	0	0	0	0	0
0	0	0	0	0	0	0	0	0
0	0	0	0	0	0	0	0	0
0	0	0	0	0	0	0	0	0
0	0	0	0	0	0	0	0	0
0	0	0	0	8	0	0	0	0
0	0	0	0	0	0	0	0	0
0	0	0	0	0	0	0	0	0
0	0	0	0	0	0	0	0	0
0	0	0	0	0	0	0	0	0
0	0	0	0	0	0	0	0	0
0	0	0	0	0	0	0	0	0
0	0	0	0	0	0	0	0	0
0	0	0	0	0	0	0	0	0
0	0	0	0	0	0	0	0	0
0	0	0	0	0	0	0	0	0
0	0	0	0	0	0	0	0	0
0	0	0	0	0	0	0	0	0
0	0	0	0	0	0	0	0	0
0	0	0	0	0	0	0	0	0
0	0	0	0	0	0	0	0	0
0	0	0	0	0	0	0	0	0
0	0	0	0	0	0	0	0	0
0	0	0	0	0	0	0	0	0
0	0	0	0	0	0	0	0	0
0	0	0	0	0	0	0	0	0
0	0	0	0	0	0	0	0	0
0	0	0	0	0	0	0	0	0
0	0	0	0	0	0	0	0	0
0	0	0	0	0	0	0	0	0
0	0	0	0	0	0	0	0	0
0	0	0	0	0	0	0	0	0
5	0	0	0	0	0	0	0	0
0	0	0	0	0	0	0	0	0

0	0	0	0	0	0	0	0	0
0	0	0	0	0	0	0	0	0
0	5	0	0	0	0	0	0	0
0	0	0	0	0	0	0	0	0
0	0	0	0	0	0	0	0	0
0	0	0	0	0	0	0	0	0
0	0	0	0	0	0	0	0	0
0	0	0	0	0	0	0	0	0
0	0	0	0	0	0	0	0	0
0	0	0	0	0	0	0	0	0
0	0	0	0	0	0	0	0	0
0	0	0	0	0	0	0	0	0
0	4	0	0	0	0	0	0	0
0	0	0	0	0	0	0	0	0
0	0	0	0	0	0	0	0	0
0	0	0	0	0	0	0	0	0
0	0	0	0	0	0	0	0	0
0	0	0	0	0	0	0	0	0
0	0	4	0	0	0	0	0	0
0	0	0	0	0	0	0	0	0
0	0	0	0	0	0	0	0	0
0	0	0	0	0	0	0	0	0
0	0	0	0	0	0	0	0	0
0	0	0	0	0	0	0	0	0
0	0	0	0	0	0	0	0	0
0	0	0	0	0	0	0	0	0
0	0	0	0	0	0	0	0	0
0	0	0	0	0	0	0	0	0
0	0	0	0	0	0	0	0	0
0	0	0	0	0	0	0	0	0
0	0	0	0	0	3	0	0	0
0	0	0	0	0	0	0	0	0
0	0	0	0	0	0	0	0	0
0	0	0	0	0	0	0	0	0
0	0	0	0	0	0	0	0	0
0	0	0	0	0	0	0	0	0
0	0	0	0	0	0	0	0	0
0	0	0	0	0	0	0	0	0
0	0	0	0	0	0	0	0	0
0	0	0	0	0	0	0	0	0
0	0	0	0	0	0	0	0	0
0	0	0	0	0	0	0	0	0
0	0	0	0	0	0	0	0	0

0	0	0	0	0	0	0	0	0
0	0	0	0	0	0	0	0	0
0	0	0	0	0	0	0	0	0
0	0	0	0	0	0	0	0	0
0	0	0	0	0	0	0	0	0
0	0	0	0	0	0	0	0	0
0	0	0	0	0	0	0	0	0
0	0	0	0	0	0	0	0	0
0	0	0	0	0	0	0	0	0
0	0	0	0	0	0	0	0	0
0	0	0	0	0	0	0	0	0
0	0	0	0	0	0	0	0	0
0	0	0	0	0	0	0	0	0
0	0	0	0	0	0	0	0	0
0	0	0	0	0	0	0	0	0
0	0	0	0	0	0	0	0	0
0	0	0	0	0	0	0	0	0
0	0	0	0	0	0	0	0	0
0	0	0	0	0	0	0	0	0
0	0	0	0	0	0	0	0	0
0	0	0	0	0	0	0	0	0
0	0	0	0	0	0	0	0	0
0	0	0	0	0	0	0	0	0
0	0	0	0	0	0	0	0	0
0	0	0	0	0	0	0	0	0

LC69	HC56	HC57	HC58	HC59	HC60
5808	29980	18418	41082	31897	8428
123	91	84	81	76	63
35	1731	2050	39	4818	3959
73	899	401	451	478	3142
158	29	48	385	55	2105
0	42	1215	10	15	2072
25892	1247	1394	18	1506	760
63	737	440	2740	622	1537
0	257	522	0	59	692
42	205	25	735	1208	24138
0	419	0	6	322	0
44	47	70	64	18	21
0	62	17	25	30	444
0	105	491	379	223	224
17289	96	35	589	76	22
0	542	299	817	1102	2580
0	66	47	17	19	0
0	12	473	128	2634	752
0	3856	2076	2957	130	150
0	8	237	0	0	621
0	4	225	0	470	608
0	9	111	6	313	904
0	6	985	0	7	0
2394	8	0	0	0	13
0	0	3624	9	257	73
0	15	1639	4	0	212
53	198	127	155	116	42
0	2039	0	3493	2	0
0	208	0	212	304	259
0	9	556	0	3490	0
0	47	171	0	314	793
0	140	33	610	0	1565
0	0	225	0	0	35
0	12	69	0	0	0
742	0	0	28	0	5
0	43	287	3	149	351
0	0	90	66	39	296
0	0	0	0	85	13
0	219	125	542	720	771
0	139	100	594	332	979
0	14	102	0	307	117
5	0	0	0	0	3

0	0	14	0	223	117
0	0	0	0	0	0
0	0	82	0	155	813
0	183	104	185	303	1098
0	272	38	28	25	61
3	3	0	51	5	17
0	5	15	6	13	0
0	6	124	0	0	0
0	0	152	2	93	640
0	0	0	0	0	43
11	173	183	0	100	25
3345	11	0	9	0	50
0	19	39	121	81	399
8	14	6	6	0	4
0	196	67	91	38	57
0	0	0	0	0	0
0	0	419	4	68	283
0	0	0	0	0	44
0	186	238	5	0	167
0	0	57	0	71	10
0	108	6	95	105	88
0	0	23	0	0	223
10	15	0	29	8	0
0	0	456	0	554	0
6755	0	0	0	0	0
0	0	0	19	13	8
0	300	44	56	386	0
0	50	24	11	32	145
0	0	0	0	0	24
4	0	3	0	0	0
0	0	35	0	0	13
0	0	0	0	0	0
0	7	11	45	62	112
0	16	0	5	0	9
4	0	0	0	0	0
0	58	0	0	8	0
0	7	0	12	13	32
0	130	38	0	72	78
2	0	8	10	0	18
0	0	0	0	0	0
0	0	61	218	186	0
0	0	0	0	0	0
6	26	15	0	21	37

0	0	0	0	0	0
0	0	0	0	0	36
0	0	72	0	0	0
0	0	79	0	0	0
0	0	0	0	0	0
0	11	0	12	26	8
0	0	0	0	0	0
15	33	30	15	14	12
0	0	4	0	0	0
0	0	0	4	0	9
0	0	0	0	0	0
10	13	11	0	0	0
0	0	0	0	0	0
0	0	3	0	0	130
0	0	13	0	0	699
0	0	0	0	0	6
0	20	0	0	0	0
0	0	0	0	0	0
0	0	0	0	0	0
0	0	0	0	0	0
0	5	0	0	0	0
0	0	0	0	0	0
0	0	128	0	0	0
0	0	0	0	0	23
0	6	0	0	0	0
0	0	13	0	0	226
8	56	13	17	9	0
0	0	0	0	0	0
0	0	0	0	0	0
0	0	0	0	0	0
0	0	0	0	0	0
0	0	0	0	0	8
0	0	0	0	10	0
0	12	0	0	0	0
0	0	0	0	0	0
0	0	0	0	0	15
0	0	0	19	0	7
0	0	0	0	0	0
0	0	0	0	0	0
0	0	0	0	0	0
691	0	0	0	0	0
0	0	0	0	19	26
0	0	0	0	0	0

0	0	0	0	73	0
0	0	0	0	17	14
0	0	0	0	0	23
0	0	0	0	0	0
0	0	0	0	0	101
0	0	0	0	0	0
0	0	0	0	0	10
0	0	0	0	0	0
0	6	0	0	7	25
0	0	0	0	0	0
0	0	0	26	0	19
0	9	13	0	0	3
0	0	0	0	0	0
0	0	0	0	0	0
0	0	0	0	0	0
0	0	0	0	0	0
0	0	0	0	0	0
0	27	0	0	0	0
0	0	17	0	0	0
0	0	0	0	6	0
0	5	0	5	0	12
0	3	0	0	4	0
0	5	23	23	16	0
0	0	0	0	0	0
0	0	0	0	0	0
0	0	0	0	0	0
0	0	0	0	3	55
0	0	0	0	0	0
0	0	0	0	0	0
244	4	0	0	0	0
0	0	0	0	0	0
0	0	0	0	0	0
0	0	0	0	0	0
0	0	0	0	0	0
0	0	0	0	0	0
0	0	0	0	0	0
0	0	0	0	0	0
0	0	0	0	0	0
0	0	0	0	0	0
0	0	0	0	0	34
0	0	0	0	0	0
0	0	0	0	0	0
0	0	0	0	0	0

0	0	0	0	0	0
0	0	0	0	0	0
0	0	0	0	0	0
0	21	0	0	0	0
0	0	0	0	0	0
0	0	0	0	0	0
0	0	0	0	0	0
0	0	0	0	0	0
0	0	0	0	0	0
0	0	0	0	0	15
0	0	0	0	0	0
0	0	0	0	0	0
0	0	0	0	3	9
0	0	0	0	0	0
0	0	0	0	0	0
0	0	0	0	0	3
0	0	0	0	0	0
0	16	0	0	0	0
0	0	0	0	0	0
0	0	0	0	5	0
0	0	0	0	0	0
0	0	0	0	0	14
0	0	0	0	0	0
0	0	0	0	0	0
0	0	0	0	0	0
0	0	0	0	0	0
0	0	0	0	0	0
0	0	3	0	0	0
0	0	0	0	0	0
0	0	0	0	0	0
0	0	0	0	0	0
0	0	0	0	6	2
0	10	0	0	0	0
0	0	0	0	0	0
0	0	0	0	0	0
0	0	0	0	0	0
0	0	0	0	0	0
0	0	0	0	0	0
0	0	0	0	17	0
0	0	0	0	0	0
0	0	0	0	0	0
0	0	0	0	0	0
0	0	0	0	0	11

0	0	0	0	0	0
0	0	0	0	0	0
0	0	0	0	0	0
0	0	0	0	0	23
0	0	0	0	0	0
0	0	0	0	0	0
0	0	0	0	0	0
0	0	0	0	0	0
0	0	0	0	0	0
0	0	0	0	0	0
0	0	0	0	0	0
0	0	0	0	0	0
0	0	0	0	0	0
0	0	0	0	0	0
0	0	0	0	0	0
0	0	0	0	0	0
0	0	0	0	0	0
0	0	0	0	0	2
0	0	0	0	0	0
0	0	0	0	0	0
0	4	0	0	0	0
0	0	0	0	0	0
0	0	0	0	0	4
0	0	0	0	0	0
0	0	0	0	0	0
0	0	0	0	0	0
0	0	0	0	0	0
0	0	0	0	0	0
0	0	0	0	0	0
0	0	0	0	0	0
0	5	0	0	0	0
0	0	0	0	0	0
0	0	0	0	0	0
0	0	0	0	0	0
0	0	0	0	0	0
0	0	0	0	0	0
0	0	0	2	0	0
0	0	0	0	0	0
0	0	0	0	0	0
0	0	0	0	0	0
0	0	0	0	0	0
0	0	0	0	0	0
0	0	0	0	0	0

0	0	0	0	0	0
0	0	0	0	0	0
0	0	0	0	5	0
0	0	0	0	0	0
0	0	0	0	0	0
0	0	0	0	0	0
0	0	0	0	0	0
0	0	0	0	0	0
0	0	0	0	0	0
0	0	0	0	0	0
0	0	0	0	0	0
0	0	0	0	0	0
0	0	0	0	0	0
0	0	0	0	0	0
0	0	0	0	0	0
0	0	0	0	0	0
0	0	0	0	0	0
0	0	0	0	0	0
0	0	0	0	0	0
0	0	0	0	0	0
0	0	0	0	0	0
0	0	3	0	0	0
0	0	0	0	0	0
0	0	0	0	0	0
0	0	0	0	0	0
0	0	0	0	0	0
0	0	0	0	0	0
0	0	0	0	0	0
0	0	0	0	0	0
0	0	0	0	0	0
0	0	0	0	0	0
0	0	0	0	0	0
0	0	0	0	0	0
0	0	0	0	0	0
0	0	0	0	0	0
0	0	0	0	0	0
0	0	0	0	0	6
0	0	0	0	0	0
0	0	0	0	0	0
0	0	0	0	0	0
0	0	0	0	0	0
0	0	0	0	0	0
0	0	0	0	0	0

0	0	0	0	0	0
0	0	0	0	0	0
0	0	0	0	0	0
0	0	0	0	0	0
0	0	0	0	0	0
0	0	0	0	0	0
0	0	0	0	0	0
0	0	0	0	0	0
0	0	0	0	0	0
0	0	0	0	0	0
0	0	0	0	0	0
0	4	0	0	0	0
0	0	0	0	0	0
0	0	0	0	0	0
0	0	0	0	0	0
0	0	0	0	0	0
0	0	0	0	0	0
0	0	0	0	0	0
0	0	0	0	0	0
0	0	0	0	0	0
0	0	0	0	0	0
0	0	0	0	0	0
0	0	0	4	0	0
0	0	0	0	0	0
0	0	0	0	0	0
0	0	0	0	0	0
0	0	0	0	0	0
0	0	0	0	0	0
0	0	0	0	0	0
0	0	0	0	0	0
0	0	0	0	0	0
0	0	0	0	0	0
0	0	0	0	0	0
0	0	0	0	0	0
0	0	0	0	0	0
0	0	0	0	0	0
0	0	0	0	0	0
0	0	0	0	0	0
0	0	0	0	0	0
0	0	0	0	0	0
0	0	0	0	0	0
0	0	0	0	0	0
0	0	0	0	0	0

0	0	0	0	0	0
0	0	0	0	0	0
0	2	0	0	0	0
0	0	0	0	0	0
0	0	0	0	0	0
0	2	0	0	0	0
0	0	0	0	0	0
0	0	0	0	0	0
0	0	0	0	0	0
0	0	0	0	0	0
0	0	0	0	0	0
0	0	0	0	0	0
0	0	0	0	0	0
0	0	0	0	0	0
0	0	0	0	0	0
0	0	0	0	0	0
0	0	0	0	0	0
0	0	0	0	0	0
0	2	0	0	0	0
0	0	0	0	0	0
0	0	0	0	0	0
0	0	0	0	0	0
0	0	0	0	0	0
0	0	0	0	0	0
0	0	0	0	0	0
